# Computational Mutagenesis at the SARS-CoV-2 Spike Protein/Angiotensin-Converting Enzyme 2 Binding Interface: Comparison with Experimental Evidence

**DOI:** 10.1021/acsnano.0c10833

**Published:** 2021-03-18

**Authors:** Erik Laurini, Domenico Marson, Suzana Aulic, Alice Fermeglia, Sabrina Pricl

**Affiliations:** † Molecular Biology and Nanotechnology Laboratory (MolBNL@UniTS), DEA, University of Trieste, 34127 Trieste, Italy; ‡ Department of General Biophysics, Faculty of Biology and Environmental Protection, University of Lodz, 90-136 Lodz, Poland

**Keywords:** SARS-CoV-2 spike protein, ACE2, receptor binding domain, molecular dynamics, computational mutagenesis, molecular mechanics/Poisson−Boltzmann surface area (MM/PBSA), free energy of binding

## Abstract

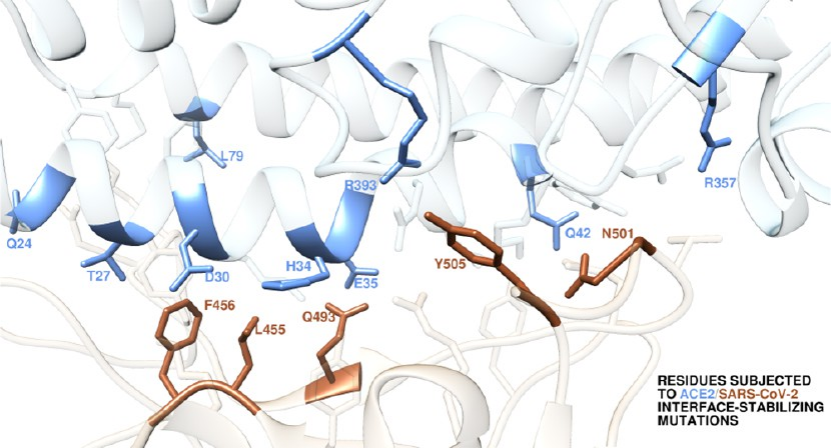

The coronavirus disease-2019 (COVID-19) pandemic, caused by the pathogen severe acute respiratory syndrome coronavirus 2 (SARS-CoV-2), started in China during late 2019 and swiftly spread worldwide. Since COVID-19 emergence, many therapeutic regimens have been relentlessly explored, and although two vaccines have just received emergency use authorization by different governmental agencies, antiviral therapeutics based neutralizing antibodies and small-drug inhibitors can still be vital viable options to prevent and treat SARS-CoV-2 infections. The viral spike glycoprotein (S-protein) is the key molecular player that promotes human host cellular invasion via recognition of and binding to the angiotensin-converting enzyme 2 gene (ACE2). In this work, we report the results obtained by mutating *in silico* the 18 ACE2 residues and the 14 S-protein receptor binding domain (S-RBD_CoV-2_) residues that contribute to the receptor/viral protein binding interface. Specifically, each wild-type protein–protein interface residue was replaced by a hydrophobic (isoleucine), polar (serine and threonine), charged (aspartic acid/glutamic acid and lysine/arginine), and bulky (tryptophan) residue, respectively, in order to study the different effects exerted by nature, shape, and dimensions of the mutant amino acids on the structure and strength of the resulting binding interface. The computational results were next validated *a posteriori* against the corresponding experimental data, yielding an overall agreement of 92%. Interestingly, a non-negligible number of mis-sense variations were predicted to enhance ACE2/S-RBD_CoV-2_ binding, including the variants Q24T, T27D/K/W, D30E, H34S7T/K, E35D, Q42K, L79I/W, R357K, and R393K on ACE2 and L455D/W, F456K/W, Q493K, N501T, and Y505W on S-RBD_CoV-2_, respectively.

The Coronavirus Disease 2019 (COVID-19) is a contagious infection caused by the severe acute respiratory syndrome (SARS) coronavirus (CoV) 2 (SARS-CoV-2).^1^ The first case was identified in Wuhan, China, in December 2019,^2^ and, since then, it has been spreading worldwide leading to the currently ongoing pandemic. COVID-19 symptoms are highly variable, although the most commonly reported include fever, cough, fatigue, breathing difficulties, and loss of smell and taste.^3^ While many individuals report mild symptoms 1 to 14 days after viral exposure, unfortunately a large portion of the affected population develops acute respiratory distress syndrome (ARDS).^4^ If not treated promptly and efficiently, ARDS can dramatically turn into cytokine storms,^5^ multiorgan failure,^6^ septic shock,^7^ and blood clots.^8^ Longer-term damage to organs (lungs and heart, in particular) has also been reported.^9^ Last but certainly not least, there is a growing concern about the number of patients who, after recovering from COVID-19 acute phase, continue to experience a range of disease effects—known as long COVID—for months afterward.^10^

After the adoption of strong measures to counteract the pandemic outbreak and the subsequent successful containment of viral spreading, unfortunately many countries around the world are now witnessing a resurgence in COVID-19. As accessed by December 21, 2020, the World Health Organization (WHO)^11^ reports 75,479,471 confirmed cases of COVID-19, including 1,686,267 deaths. The most afflicted continents are the Americas (U.S.A. and Brazil, in particular) and Europe, with 32,740,713 and 23,673,404 documented cases, respectively. On December 11, 2020, the U.S. Food and Drug Administration (FDA) issued the first emergency use authorization (EUA) for the vaccine produced by Pfizer-BioNTech for COVID-19 prevention in individuals 16 years of age and older; a week later, FDA further issued the EUA for the second vaccine against SARS-CoV-2 (Moderna) for use in individuals 18 years of age and older. At the same time, after the European Medicine Agency (EMA) recommended granting a conditional marketing authorization for the Pfizer/BioNTech vaccine, the final EUA was released during the extraordinary meeting of the European Committee for Medicinal Products for Human Use (CHMP) on December 21, 2020. Notwithstanding, under the pressure of the ever-growing number of infected people and the upcoming holiday seasons, many countries are again imposing full or partial lockdown conditions in the attempt to contain viral spreading, exacerbating negative economic consequences^12,13^ and psychological distress.^14^

Similarly to SARS-CoV—the pathogen responsible for the 2002–2003 SARS pandemic that also initially emerged in China (Guangdong province) and swiftly globally spread via air-travel routes infecting more than 8000 people with a 10% death toll^15^—SARS-CoV-2 exploits the homotrimeric transmembrane spike glycoprotein (S-protein) during host cell invasion.^16,17^ During this complex process,^18^ a well-characterized S-protein region—the receptor binding domain (S-RBD)—specifically engages the angiotensin-converting enzyme 2 (ACE2) as its cellular receptor.^19−23^ In addition, viral cell entry involves S-protein priming promoted by the cellular transmembrane serine protease 2 (TMPRSS2) or other proteases.^24^ So far, it is well-established that host susceptibility to SARS-COV-2 is primarily determined by the binding affinity of the viral S-RBD for ACE2 during the initial viral attachment step;^19−23^ moreover, computer-based simulations of the interaction between the S-RBD of SARS-CoV-2 (S-RBD_CoV-2_) and ACE2 performed by our group^25^ have identified some residues that play a major role across the human receptor/viral protein binding interface. In particular, the adopted computational alanine scanning (CAS) approach highlighted residues D38, K31, E37, K353, and Y41 on ACE2 and Q498, T500, and R403 on the SARS-CoV-2 S-protein receptor binding domain (Figure 1) as hot spots contributing to shaping and determining the stability of the relevant protein–protein interface.

**Figure 1:**
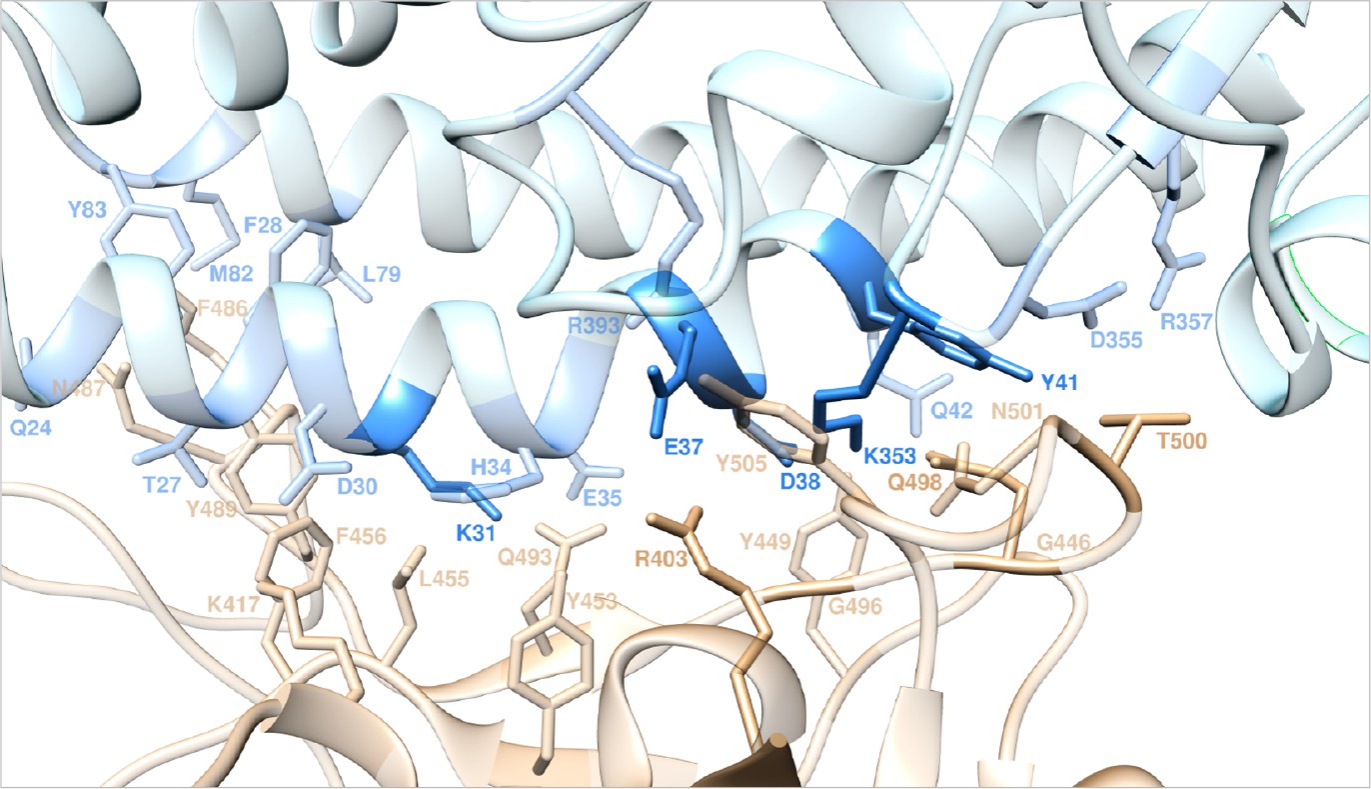
Structural details of the binding interface between ACE2 and the viral spike protein receptor binding domain of SARS-CoV-2 (S-RBD_CoV-2_) showing the hot-spot residues at the protein/protein binding interface as identified in our previous computational alanine scanning study.^25^ The secondary structures of ACE2 and S-RBD_CoV-2_ are portrayed as light blue and light tan ribbons, respectively. Each interacting protein residue is shown in light matching-colored sticks and labeled, with hot-spot residues highlighted in the corresponding dark colors.

During the peer review process of our previous work on the subject,^25^ one of the experts offered the suggestion to expand the computational approach beyond alanine scanning, and prompted us to challenge our *in silico* framework against non-alanine mutations that may arise at the ACE2/S-protein interface residues. Indeed, as SARS-CoV-2 utilizes ACE2 as host receptor and host proteases for cell surface binding and internalization, it is possible that genetic variability of this human receptor could constitute one of the elements modulating virion intake and, therefore, disease severity. In this respect, for instance, Cao and colleagues already reported and characterized 32 variants in different populations.^26^ Contextually, another large study—in which the whole-exome sequencing data of 6930 Italian control individuals from 5 different centers looking for ACE2 variants—also identified a number of ACE2 mutations with a potential impact on protein stability and/or S-protein binding.^27^ Accordingly, it is conceivable that ACE2 gene polymorphism, human ACE2 mRNA expression, and human ACE2 protein polymorphism all might exert an influence on SARS-CoV-2 susceptibility and COVID-19 disease outcome.^28^

From the viral standpoint, it is well-known that mutations in all RNA viruses (including SARS-CoV-2) can easily originate at least from three main processes. First of all, copying errors during viral replication are perhaps the first, intrinsically most common source of mutations.^29^ Interestingly, this process may be less relevant for SARS-CoV-2 with respect to other RNA viruses, since coronavirus polymerases are endowed with a proofreading mechanism.^30^ Next, genomic variability might arise as the result of recombination between two viral lineages coinfecting the same host.^31^ Finally, mutations can be induced by host RNA-editing systems, which form part of natural host immunity.^32,33^ In addition, the recent results published by Di Giorgio and co-workers^33^ suggest that both the apolipoprotein B mRNA editing catalytic polypeptide-like (APOBEC) and adenosine deaminases acting on RNA (ADAR) families of proteins are involved in coronavirus genome editing, a process that may shape the fate of both virus and patient. Population genetics and epidemiology of RNA viruses teach us that mutation is an inevitable consequence of being a virus.^34^ However, those mutations that adversely impact vital steps of virus function are quickly removed by natural selection; conversely, slight deletions might be retained (if only transiently) while neutral and in particular advantageous mutations can reach higher frequencies.

Under this perspective, the possibility to estimate the difference in binding affinity between different allelic variants of ACE2 or S-RBD_CoV-2_ could be additionally exploited in, *e.g.*, (i) understanding further the specificity of interaction between the viral protein and its receptor, (ii) effective structure-based design and development of neutralizing antibodies, vaccines, and protein/protein inhibitors, and, above all, (iii) assessing the role of a genetic diversity among population/individuals. As a consequence, we embarked on the effort of mutating each of the 18 ACE2 residues and the 14 residues of S-RBD_CoV-2_ at the relevant protein/protein binding interface. In detail, we selected to mutagenize every native protein–protein interface residue into a hydrophobic (isoleucine), polar (serine and threonine), charged (aspartic acid/glutamic acid and lysine/arginine), and bulky (tryptophan) residue, respectively, in order to study the different effects exerted by nature, shape, and dimensions of the mutant amino acids on the structure and strength of the resulting binding interface. Quite importantly, while undertaking the extensive campaign of fully atomistic molecular dynamics (MD) simulations (196 simulations), two experimental papers based on deep mutagenesis and fluorescence-activated cell sorting (FACS) techniques established the effects of mutations in ACE2^35^ and S-protein^36^ residues, respectively, across the binding interface. Although the majority of changes in both proteins were found to be deleterious for protein expression and mutual binding, a significant number of residue variations were reported to be well tolerated or even to enhance protein/protein binding in both cases.^35,36^ Accordingly, we used these experimental data to benchmark and validate the results of the current study. To this purpose, initially a color-coded criterion based on the predicted free energy difference range of values was independently adopted, as shown in Table 1.

**Table 1:** Color-Coded Criterion Based on the Predicted Free Energy Difference (ΔΔ*G*) Range of Values Adopted to Compare *in Silico* Results and Experimental Evidence^a^

mutation effect	ΔΔ*G* range (kcal/mol)	color code
Neutral mutations	–0.25 ≤ ΔΔ*G* ≤ +0.25	Gray
Mildly destabilizing mutations	–2.00 ≤ ΔΔ*G* < −0.25	Light Yellow
Destabilizing mutations	–4.00 ≤ ΔΔ*G* < −2.00	Light Red
Highly destabilizing mutations	–4.00 < ΔΔ*G*	Red
Stabilizing mutations	+0.25 < ΔΔ*G* ≤ +1.00	Light Green
Highly stabilizing mutations	ΔΔ*G* > +1.00	Green

a Negative/positive ΔΔ*G* values indicate unfavorable/favorable substitutions for the mutant residue in the relevant position, respectively.

The mutation ranking thus obtained was then compared to the results—expressed using an utterly analogous color-code (or heat map)—reported by Chan et al.^35^ for the ACE2 variants and by Starr and co-workers^36^ for the S-protein isoforms, respectively.

For the sake of brevity, in what follows only those ACE2 and S-RBD_CoV-2_ mutations expected to increase the stability of the corresponding protein/protein binding interface will be discussed in the main text, while the results for all interface disrupting/neutral mutations are given in full in the Supporting Information files. Of note, when not directly commented here, eventual disagreements between *in silico* and experimental data are also discussed in the corresponding sections of the Supporting Information.

## Results and Discussion

### Analysis of the ACE2 Residues at the Binding Interface with the S-RBD SARS-CoV-2

#### Q24 and T27

**Figure 2:**
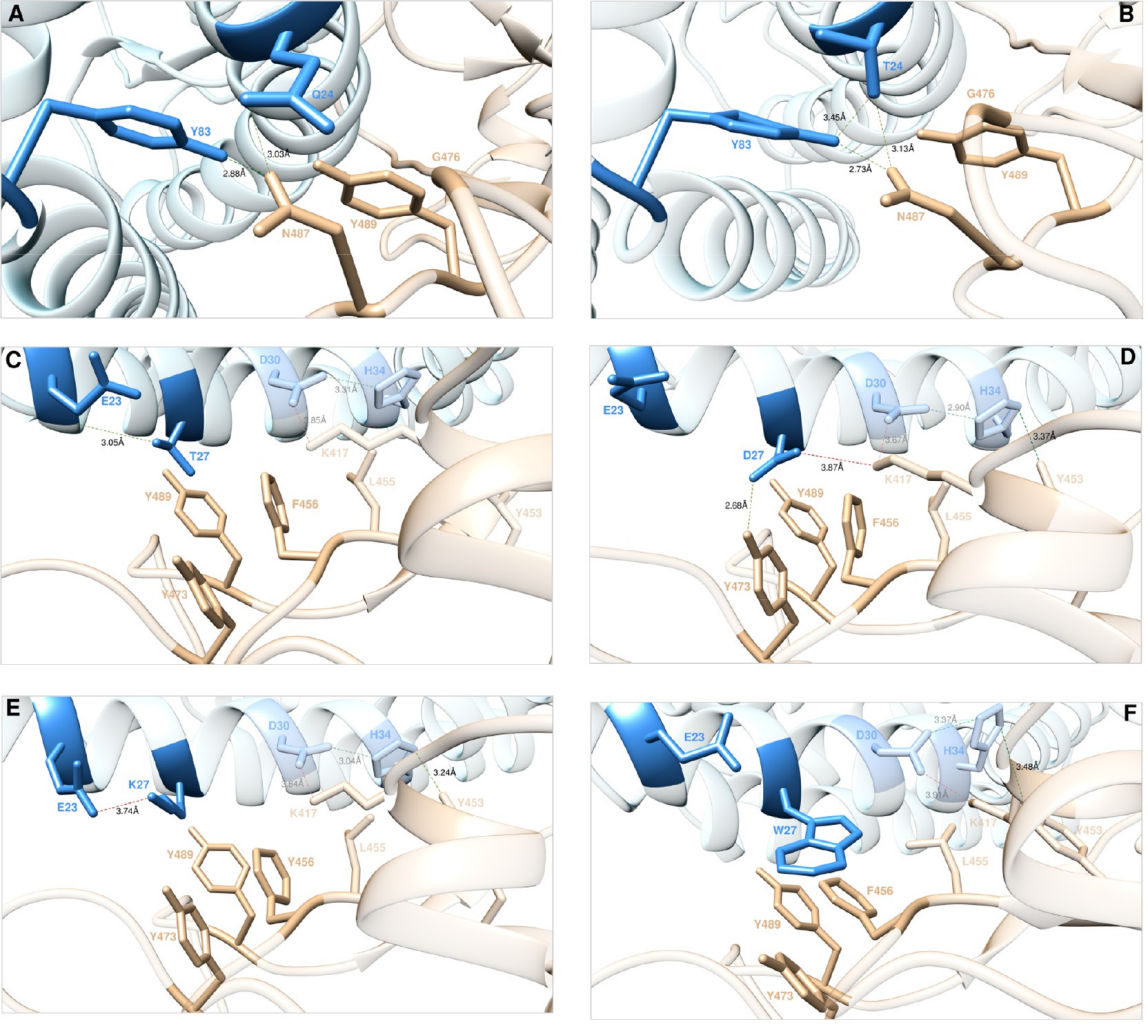
Main interactions involving the ACE2 wild-type residues Q24 (A) and the corresponding T24 mutant (B) at the interface with S-RBD_CoV-2_ as obtained from the relevant equilibrated MD simulations. Main interactions involving the ACE2 wild-type residue T27 (C) and the corresponding D27 (D), K27 (E), and W27 (F) mutants at the interface with S-RBD_CoV-2_ as obtained from the corresponding equilibrated MD simulations. Images for all other Q24 and T27 mutants are shown in Figures S1–S2, respectively. In these and all remaining figures, the secondary structures of ACE2 and S-RBD_CoV-2_ are portrayed as light blue and light tan ribbons, respectively. Each protein residue under discussion and all other residues directly interacting with them are highlighted in dark matching-colored sticks and labeled; further residues/interactions related to the residue under investigation are evidenced in light matching-colored sticks and labeled in light gray. Hydrogen bonds (HBs) and salt bridges (SBs) directly involving the mutated residues are represented as dark green and dark red broken lines, respectively, and the relevant average distances are reported (in black) accordingly; further important HBs and SBs detected in each complex are also indicated using light green/red broken lines and light gray labels (see Tables S3–S4 for details).

**Figure 3:**
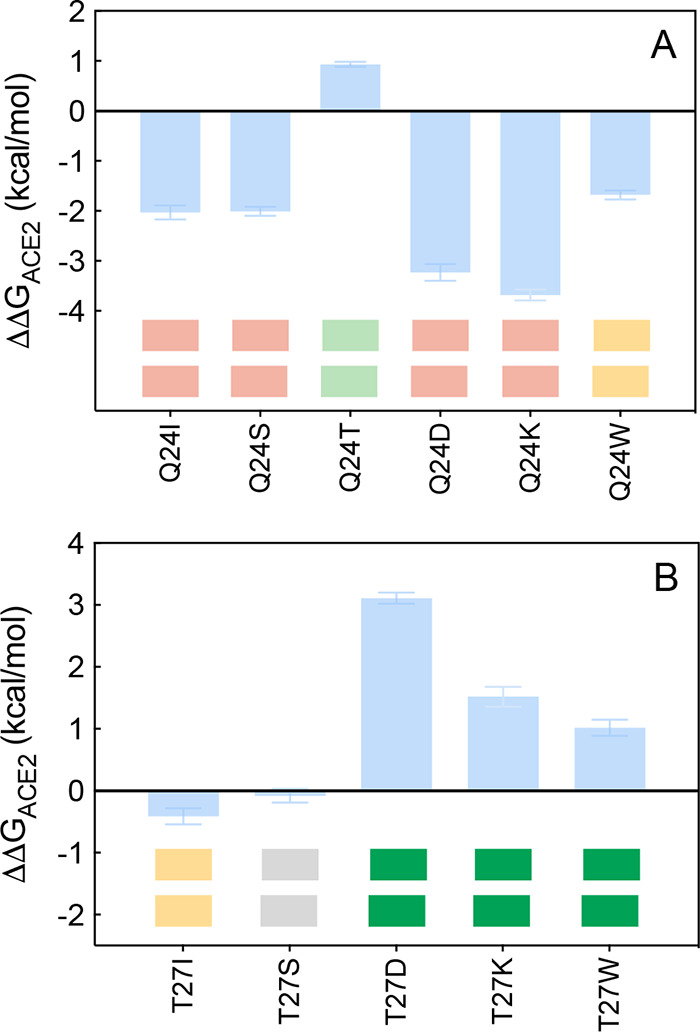
Change in binding free energy (ΔΔ*G* = Δ*G*_WILD-TYPE_ – Δ*G*_MUTANT_) predicted by computational mutagenesis of the ACE2 residues Q24 (A) and T27 (B) for the corresponding ACE2/S-RBD_CoV-2_ complexes. In each protein–protein assembly, the wild-type residue is replaced by I, S, T, D/E, K/R, and W. Negative/positive ΔΔ*G* values indicate unfavorable/favorable substitutions for the mutant residue in the relevant position, respectively. The numerical values of ΔΔ*G*, all related energy terms, and all underlying intermolecular/intramolecular interactions are reported in Table S1, Figures S1–S2, and Tables S3–S4. The colored boxes below each bar in the graphs show the qualitative comparison between *in silico* predicted (upper row) and experimental^35^ (lower row) destabilizing/stabilizing effects of the corresponding mutation on the ACE2/SARS-CoV-2 S-protein complex. Color legend: light yellow, mildly destabilizing mutations; light red, destabilizing mutations; red, highly destabilizing mutations; gray, neutral mutation; light green, stabilizing mutations; green, highly stabilizing mutations (see Table 1).

Our previous molecular dynamics (MD) simulations have revealed that the main role of the ACE2 wild-type residue Q24, located at the periphery of the receptor/S-RBD_CoV-2_ binding interface, is to anchor N487 on the S-RBD_CoV-2_*via* a stable H-bond (3.03 ± 0.18 Å), along with establishing a few weaker contact interactions (CIs) with G476 and Y489 on the viral protein.^25^ In addition, Q24 establishes an intramolecular van der Waals contact with Y83 (further discussed below), which in turn engages N487 in a complex-stabilizing H-bond (2.88 ± 0.17 Å) (Figure 2A). In line with this, our CAS of Q24 predicted an affinity decrease of the A24 mutant ACE2 for the viral protein (ΔΔ*G*_ACE2_(Q24A) = −2.61 ± 0.17 kcal/mol).^25^ In full agreement with the available experimental report,^35^ our current computational mutagenesis of Q24 into I, S, T, D, K, or W always results in a loss of the corresponding binding free energy with the exception of the T24 mutant, for which an increase in affinity for the viral protein is predicted/observed (ΔΔ*G*_ACE2_(Q24T) = +0.93 ± 0.05 kcal/mol) (Figure 3A, Table S1, Figure S1, and Table S3). Compared with the wild-type residue, T24 is able to maintain the intermolecular HB with N487 (3.13 ± 0.06 Å) and the CIs with Y489 and G476 (intermolecular) and with Y83 (intramolecular). Moreover, at variance with Q24 and all other mutants considered, ACE2 T24 directly engages Y83 in a stable intramolecular HB (3.45 ± 0.18 Å), thereby reinforcing the interaction of this ACE tyrosine with the viral N487 (2.73 ± 0.15 Å) (Figure 2B, Table S3).

In the receptor/viral protein complex, the ACE2 wild-type residue T27 affords an interface stabilizing internal HB with E23 (3.05 ± 0.16 Å), and three favorable CIs with S-RBD_CoV-2_ residues F456, Y473, and Y489 (Figure 2C). Depletion of all these interactions in the T27A mutant complex by CAS predicted a decrease of the corresponding binding free energy of −2.23 ± 0.19 kcal/mol.^25^ The actual computational mutagenesis of T27 into all residues considered reveals a variegated trend, again in complete agreement with the relevant experimental data^35^ (Figure 3B). Specifically, while only I27 induces a decrease in affinity of the human receptor for the viral protein, S27 exerts a neutral effect, whereas D, K, and W all establish extra-favorable interactions at the relevant protein/protein interfaces, ultimately increasing the affinity of the corresponding mutant isoforms for the viral S-RBD_CoV-2_ (Figure 3B, Table S1, Figure S2, and Table S4). If the analysis starts with D27, with respect to T27 this mutant residue is able to form two new, strong, and permanent intermolecular interactions with the S-protein binding domain—*i.e*., an HB with Y473 (2.68 ± 0.17 Å) and a salt bridge with K417 (3.87 ± 0.33 Å)—while preserving the CIs with F456, Y489, and Y473 (Figure 2D). Moreover, the related network of interactions seen in the wild-type complex (an intermolecular SB between D30 and K417 (3.67 ± 0.19 Å) and an internal HB between D30 and H34 (2.90 ± 0.24 Å)) not only remains unperturbed but is further reinforced *via* a new HB across the binding interface between H34 and Y453 (3.37 ± 0.21 Å) (Figure 2C,D). Accordingly, the detected loss of the wild-type internal HB between the side chain of D27 and the backbone −NH group of E23 is overcompensated by the new/preserved interactions (Table S4), in line with the experimental evidence^35^ and our predicted value of ΔΔ*G*_ACE2_(T27D) = +3.11 ± 0.09 kcal/mol (Figure 3B and Table S1). The analysis of the MD trajectory for the K27 mutant also yields a sensible explanation for the predicted/observed increase in affinity of this ACE2 variant for S-RBD_CoV-2_ (ΔΔ*G*_ACE2_(T27K) = +1.52 ± 0.16 kcal/mol (Figure 3B, Table S1, and Table S4). Indeed, as portrayed in the representative snapshot shown in Figure 2E: (i) K27 replaces the internal side chain-backbone HB between T27 and E23 with a charge-neutralizing SB at the interface (3.74 ± 0.29 Å), (ii) at variance with T27 and similarly to D27 the mutated K27 engages the viral residue Y453 in a direct intermolecular HB (3.24 ± 0.21 Å), and (iii) the presence of K27 does not alter all remaining inter/intramolecular interactions across the protein/protein interface (Figure 2C,E and Table S4). Finally, according to our prediction for the T27W mutagenesis, the loss of the internal side chain-backbone HB between W27 and E23 is more than compensated by the formation of the intermolecular HB between H34 and Y453 (3.48 ± 0.32 Å), while the network of all other interactions is fully preserved and further potentiated by increased van der Waals contacts of W27 with the side chains of Y473 and Y489 and, particularly, the π/π interaction with F456 (Figure 2F and Table S4). Therefore, in accord with the associated experimental data the estimated variation of the binding free energy for the W27 mutant ACE2/S-RBD_CoV-2_ complex is positive (Figure 3B) and equal to ΔΔ*G*_ACE2_(T27W) = +1.02 ± 0.14 kcal/mol (Table S1).

#### D30 and H34

**Figure 4:**
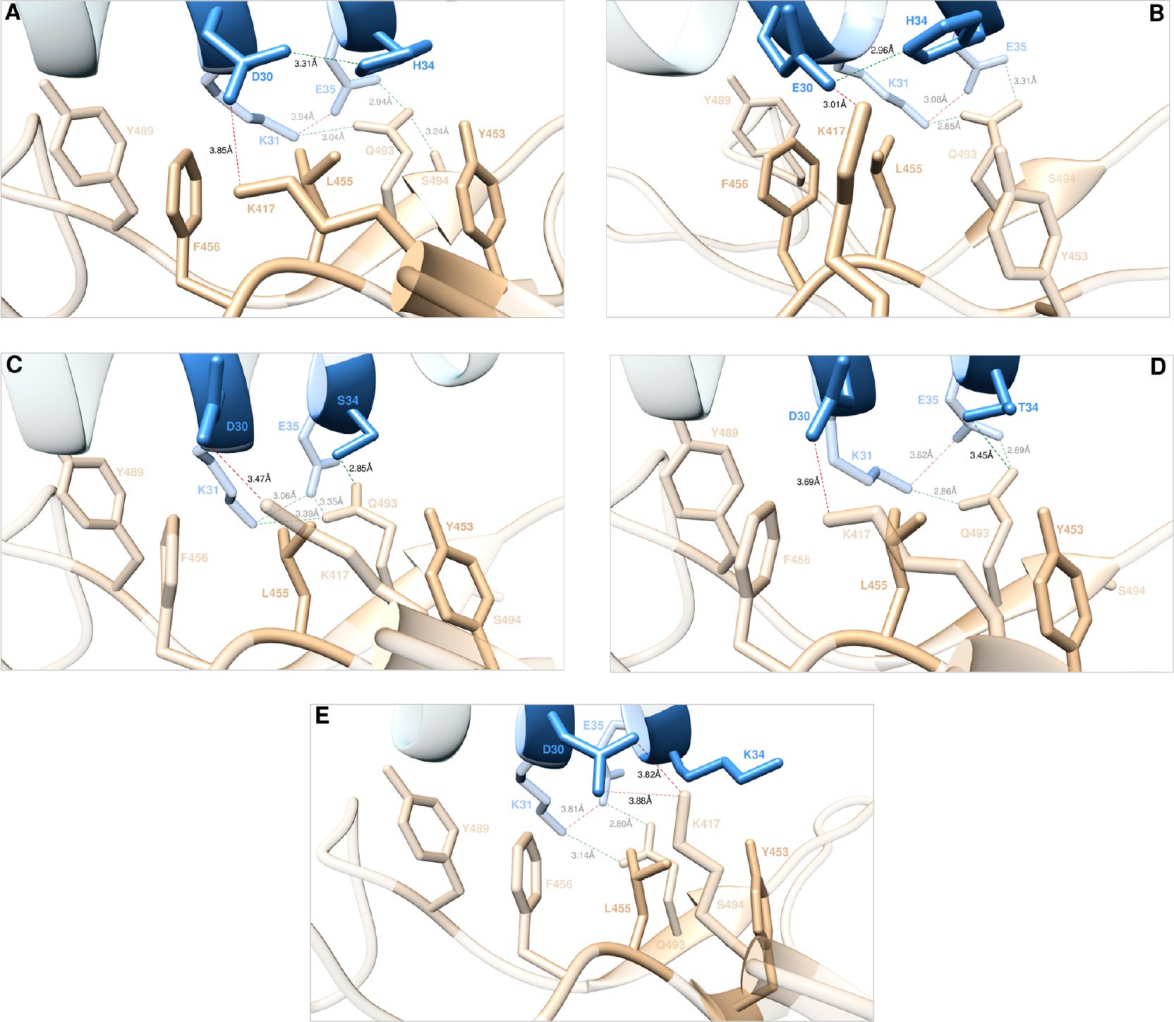
Main interactions involving the ACE2 wild-type residues D30 and H34 (A) and the corresponding E30 (B), S34 (C), T34 (D), and K34 (E) mutants at the interface with S-RBD_CoV-2_ as obtained from the relevant equilibrated MD simulations. Images for all other D30 and H34 mutants are shown in Figures S3–S4, respectively (see also Tables S5–S6 for details). Colors and other explanations are the same as in Figure 2.

**Figure 5:**
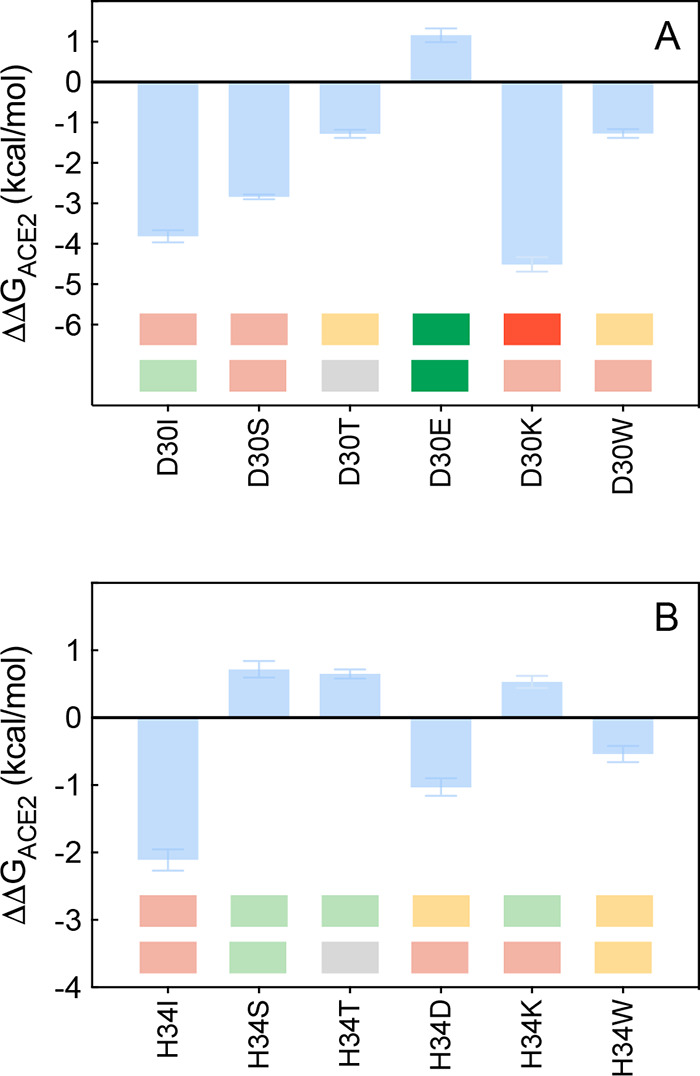
Change in binding free energy predicted by computational mutagenesis of the ACE2 residues D30 (A) and H34 (B) for the corresponding ACE2/S-RBD_CoV-2_ complexes. Colors and other explanations as in Figure 3. The numerical values of ΔΔ*G*, all related energy terms, and all underlying intermolecular/intramolecular interactions are reported in Table S1, Figures S3–S4, and Tables S5–S6.

Aspartic acid at the position 30 along the ACE wild-type sequence (D30) is another key player in the human receptor/viral protein interaction. Indeed, our CAS study and the related MD simulations^25^ confirmed that D30 establishes a critical intermolecular SB across the protein/protein interface with K417 (3.85 ± 0.41 Å) on the S-RBD_CoV-2_ (Figure 4A). In addition, D30 engages H34 of ACE2 in a stable intramolecular HB (3.31 ± 0.18 Å), contextually sharing CIs with the side chains of the protein residues F456 and L455 (Figure 4A). Accordingly, when mutagenized into alanine *in silico*, a substantial loss in affinity of the A30 mutant ACE2 isoform for S-RBD_CoV-2_ was predicted (ΔΔ*G*_ACE2_(D30A) = −3.89 ± 0.12 kcal/mol).^25^ The actual results of the computational mutagenesis of D30 into I, S, T, E, K, and W together with the experimental findings from Chan et al.^35^ are shown in Figure 5A (see also Table S1, Figure S3, and Table S5). While both data sets agree on the prevalently mild-to-strong interface destabilizing effects exerted by residues other than the wild-type D at this position, the most interesting evidence concerns the D30E mutated ACE2 receptor for which the corresponding affinity for the viral counterpart is predicted to increase, again in line with experiment (Figure 5A, Table S1; for a discussion on D30I data, see Supporting Information). The analysis of the MD trajectory for the E30 ACE2 mutant isoform in complex with S-RBD_CoV-2_ (Figure 4B) shows that, when compared with the corresponding wild-type protein/protein assembly (Figure 4A), the relevant binding interface region becomes decidedly more compact in the case of the mutant receptor, ultimately resulting in completely conserved yet shorter (and hence stronger) intermolecular interactions (*i.e*., the interfacial SB of E30 with K417 (3.01 ± 0.12 Å), the intramolecular HB between E30 and H34 (2.96 ± 0.10 Å), and the intermolecular CIs with F456 and L455 (Figure 4A,B and Table S5)). This interface tightening induced by the E30 mutation does not perturb the associated network of interactions seen in the wild-type ACE2/S-RBD_CoV-2_ complex, which include the topical ACE2 intramolecular SB between K31 and E35 (3.08 ± 0.30 Å), and the two intermolecular HBs involving the side chains again of K31 and E35 and the −CONH_2_ terminal group of the viral Q493 (2.85 ± 0.16 Å and 3.31 ± 0.24 Å, respectively; Figure 4A,B and Table S5). In summary, although the further intramolecular HB between the wild-type viral protein residues Q493 and S494 (3.24 ± 0.21 Å) is no longer detected in the corresponding E30 mutant ACE2/S-RBD_CoV-2_ complex MD trajectory (Figure 4A,B), the optimization of all remaining inter/intramolecular interactions at the related E34 mutant protein–protein interface produces a net positive increase in its affinity for the receptor binding domain of the SARS-CoV-2 spike protein (ΔΔ*G*_ACE2_(D30E) = +1.16 ± 0.17 kcal/mol, Figure 5A and Table S1).

As discussed in our previous effort,^25^ the ACE2 wild-type residue H34 plays a secondary role at the receptor/viral protein interface since besides the internal HB with D30 reviewed above, it does not involve any S-RBD_CoV-2_ residue in direct intermolecular SBs/HBs but just provides a few intermolecular CIs (i.e., dispersive and polar contacts with L455 and Y453, respectively) (Figure 4C). In line with this, mutating H34 into alanine leads to in moderate loss of ACE2 affinity for the viral spike protein (ΔΔ*G*_ACE2_(H34A) = −1.77 ± 0.09 kcal/mol).^25^ However, substituting H for I, S, T, D, K, and W at this position leads to different effects on the resultant protein–protein binding strength, with three mutant residues (S, T, and K) predicted to increase the relevant protein affinity for the S-RBD_CoV-2_, as shown in Figure 5B. In detail, when S34 is considered, the corresponding MD trajectory shows that the internal HB with D30 is replaced by an intermolecular HB across the binding interface with Q493 (2.85 ± 0.12 Å, Figure 4C). As the related network of interactions remains unaffected by the H34S mutation (Figure 4A,C, Table S6), the net result is a small but meaningful increase of the ACE2 S34 mutant affinity for the receptor binding domain of SARS-CoV-2 with respect to the wild-type (ΔΔ*G*_ACE2_(H34S) = +0.72 ± 0.12 kcal/mol, Table S1), in line with the relevant experimental data^35^ (Figure 5B). When ACE2 H34 is mutated into threonine (H34T), the MD-predicted interaction network at the corresponding protein/protein binding interface is almost identical with that described above for the S34 mutant (Figure 4C,D, Table S6); in line with this, the related value of ΔΔ*G*_ACE2_(H34T) is also positive and equal to +0.65 ± 0.07 kcal/mol (Figure 5B and Table S1). Finally, it is interesting to observe that, notwithstanding the large agreement between *in silico* and *in vitro* mutagenesis evidence for this ACE2 residue, simulation and experiment diverge when the H34K mutation is considered, since a slight interface stabilizing effect is predicted based on MD data, while the opposite behavior is reported experimentally^35^ (Figure 5B). In our case, the moderate increase in affinity of the K34 ACE2 mutant for the viral S-RBD_CoV-2_ (ΔΔ*G*_ACE2_(H34K) = +0.53 ± 0.09 kcal/mol, Table S1) is justified on the basis of the underlying inter/intramolecular interaction network illustrated in Figure 4E and Table S6. With respect to the wild-type residue H34, the isolated interfacial SB between D30 and K417 becomes bifurcated (3.82 ± 0.20 Å and 3.88 ± 0.12 Å, respectively; Figure 4E and Table S6), thereby acting as a type of molecular clip that locally tightens the protein/protein interface. Although K34 is not involved in any inter/intramolecular stabilizing SB, for the entire duration of the corresponding MD simulations its side chain protrudes into the solvent, where it engages the side chains of the viral residues Y453 and K417 in stable polar and van der Waals CI, respectively (Figure 4E, Table S6).

#### Y83, M82, L79, and F28

**Figure 6:**
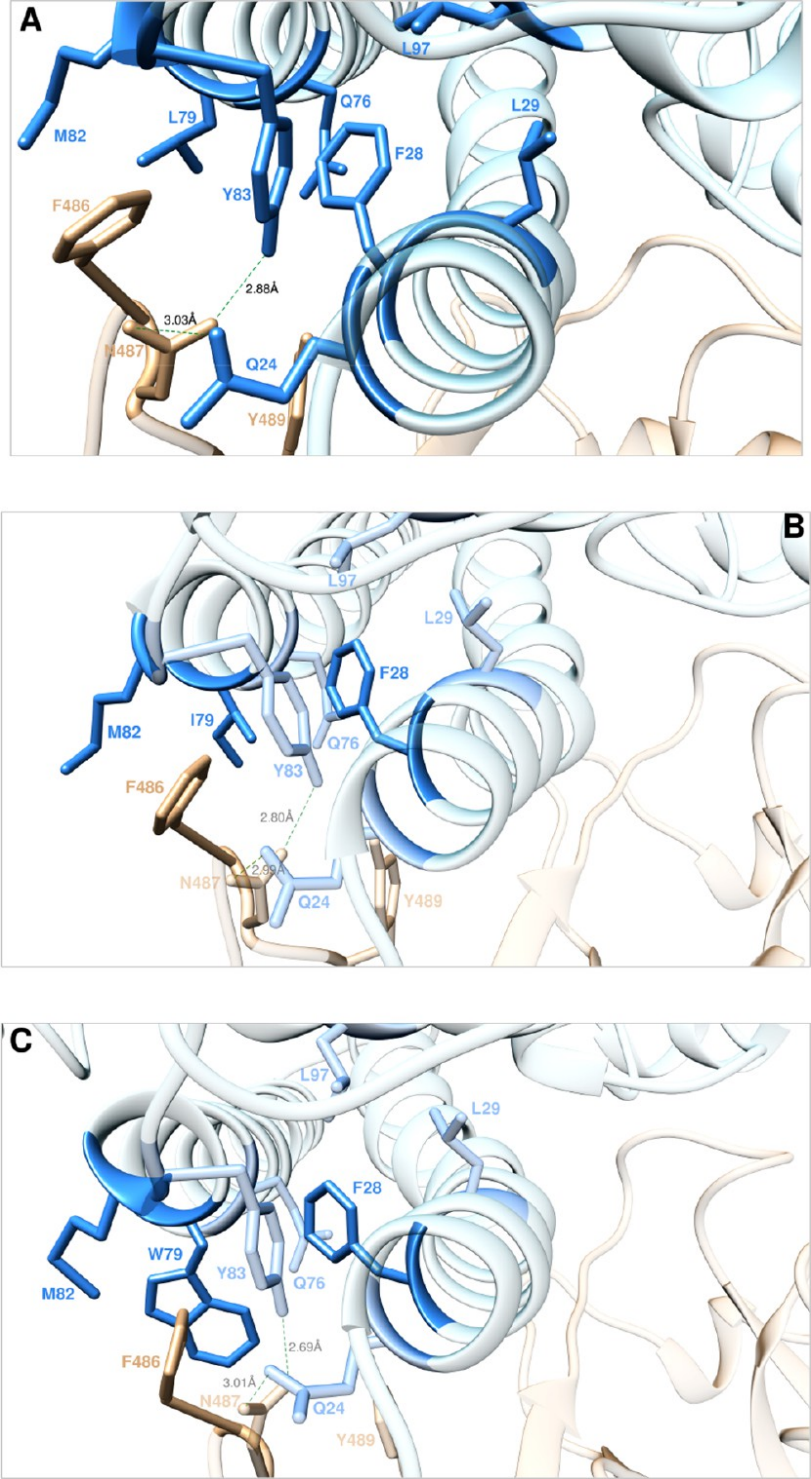
Main interactions involving the ACE2 wild-type residues Y83, M82, L79, and F28 (A) and the corresponding I79 (B) and W79 (C) mutants at the interface with S-RBD_CoV-2_ as obtained from the relevant equilibrated MD simulations. Images for all other Y83, M82, L79, and F28 mutants are shown in Figures S5–S8 (see also Tables S7–S10 for details). Colors and other explanations are as in Figure 2.

**Figure 7:**
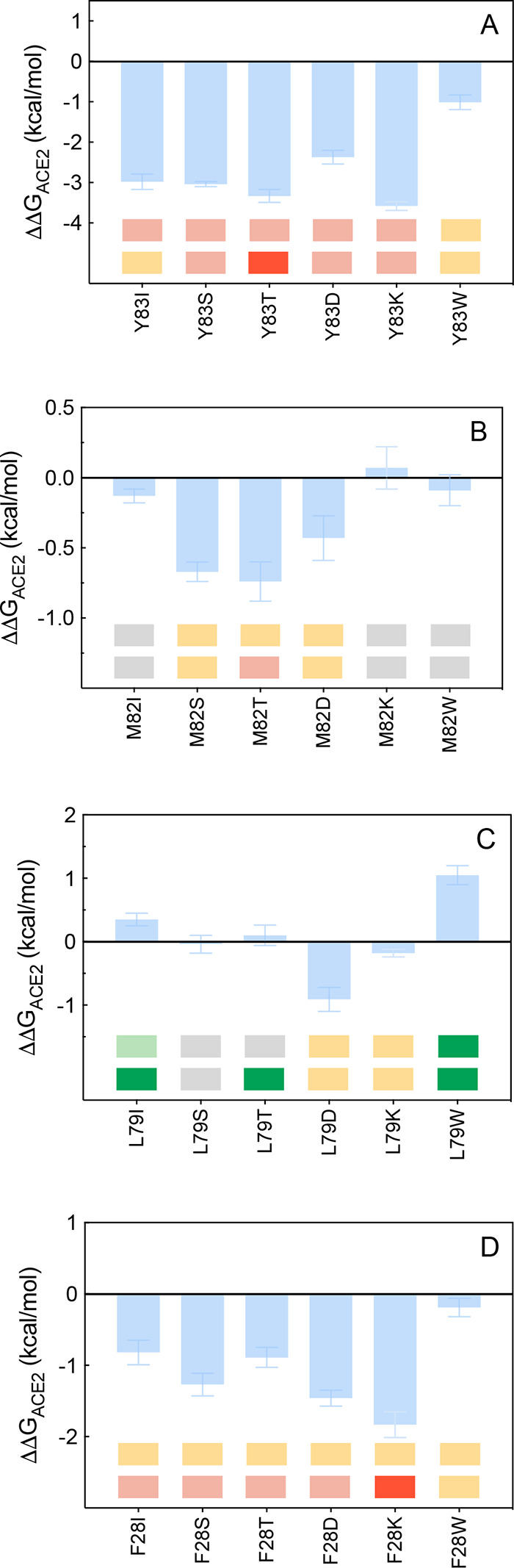
Change in binding free energy predicted by computational mutagenesis of the ACE2 residues Y83 (A), M82 (B), L79 (C), and F28 (D) for the corresponding ACE2/S-RBD_CoV-2_ complexes. Colors and other explanations as in Figure 3. The numerical values of ΔΔ*G*, all related energy terms, and all underlying intermolecular/intramolecular interactions are reported in Table S1, Figures S5–S8, and Tables S7–S10.

ACE2 Y83 is an amino acid that, as already mentioned above, plays an important role by engaging the viral N487 in a robust protein–protein HB, thereby locking this residue in place for further intermolecular H-bonding with Q24 (Figures 2A and 6A, Table S7). Y83 also engages polar and dispersive intermolecular CIs with Y489 and F486 and, intramolecularly, with the side chain of ACE2 F28 (Figure 6A, Table S6). Removing all these interactions by replacing the side chain of Y83 with alanine yielded a predicted loss of binding free energy equal to ΔΔ*G*_ACE2_(Y83A) = −3.18 ± 0.20 kcal/mol.^25^ Along the same line, mutating Y83 into I, S, T, D, K, and W always results in a destabilization of the relevant protein/protein interfaces, both computationally and experimentally^35^ (Figures 7A and S5, Table S1, and Table S7). In the same context, our previous CAS results revealed that ACE2M82 affords only weakly stabilizing intra/intermolecular CIs with L79 and F486, respectively (Figure 6A), supported by the corresponding ΔΔ*G* value predicted for the M82A mutant receptor/S-RBD_CoV-2_ complex (ΔΔ*G*_ACE2_(M82A) = −0.76 ± 0.12 kcal/mol).^25^ Consistently with this, both mutagenesis experiments^35^ and simulations entirely agree in showing neutral-to-moderately interface destabilizing effects for all alternative amino acids considered, as reported in Figures 7B and S6, Table S1, and Table S8.

The effects of mutating the ACE2 wild-type residue L79 into I, S, T, D, K, and W are more surprising, in that experimental^35^/computational mutagenesis results both predict receptor/S-RBD_CoV-2_ interface stabilization for the two substitution L79I and L79W, whereas replacement with polar (S, T) or charged residues (D, K) reflects into neutral/mildly protein/protein destabilizing effects (Figures 6B–C, S7 and 7C, Table S1, and Table S9) (for a discussion on L79T data, see Supporting Information). According to our previous CAS study, L79 is involved only in three CIs of van der Waals/hydrophobic nature, two with ACE2 residues F28 and M82 and one with the viral F486 (Figure 6B, Table S9). In keeping with this, the abrogation of these CIs within the ACE2 L79A mutant/S-RBD_CoV-2_ complex was predicted to marginally reduce the affinity of the A79 receptor for the viral S-protein (ΔΔ*G*_ACE2_(L79A) = −1.04 ± 0.16 kcal/mol).^25^ Interestingly, when L is replaced by I at the same position, the different geometry of I79 allows this amino acid to establish the same CIs of its wild-type isomer along with a slight compaction of the corresponding binding interface region (Figure 6A,B, Table S9); consequently, the predicted variation of binding free energy is slightly positive (ΔΔ*G*_ACE2_(L79I) = +0.35 ± 0.10 kcal/mol, Figure 7C, and Table S1). The effects produced by the L79W mutation are utterly similar to those just discussed for the L79I ACE2 variant, with the further engagement by W79 of F486 in a π/π interaction and enhanced dispersive interactions with the side chains of all residues shaping this hydrophobic patch of the ACE2/S-RBD_CoV-2_ binding interface (Figure 6C, Table S9). Eventually, this translates into a stabilization of the relevant protein/protein complex (ΔΔ*G*_ACE2_(L79W) = +1.05 ± 0.15 kcal/mol, Table S1), in agreement with experiment^35^ (Figure 7C). Finally, mutating the wild-type ACE2 F28—located in the center of this hydrophobic region (Figure 6A)—into all residues considered (including alanine)^25^ always results in interface destabilizing effects which, in agreement with experiment,^35^ are more significant in the case of hydrophobic-to-charged substitutions such as D and K (Figures 7D and S8, Tables S1 and S10).

#### K31 and E35

**Figure 8:**
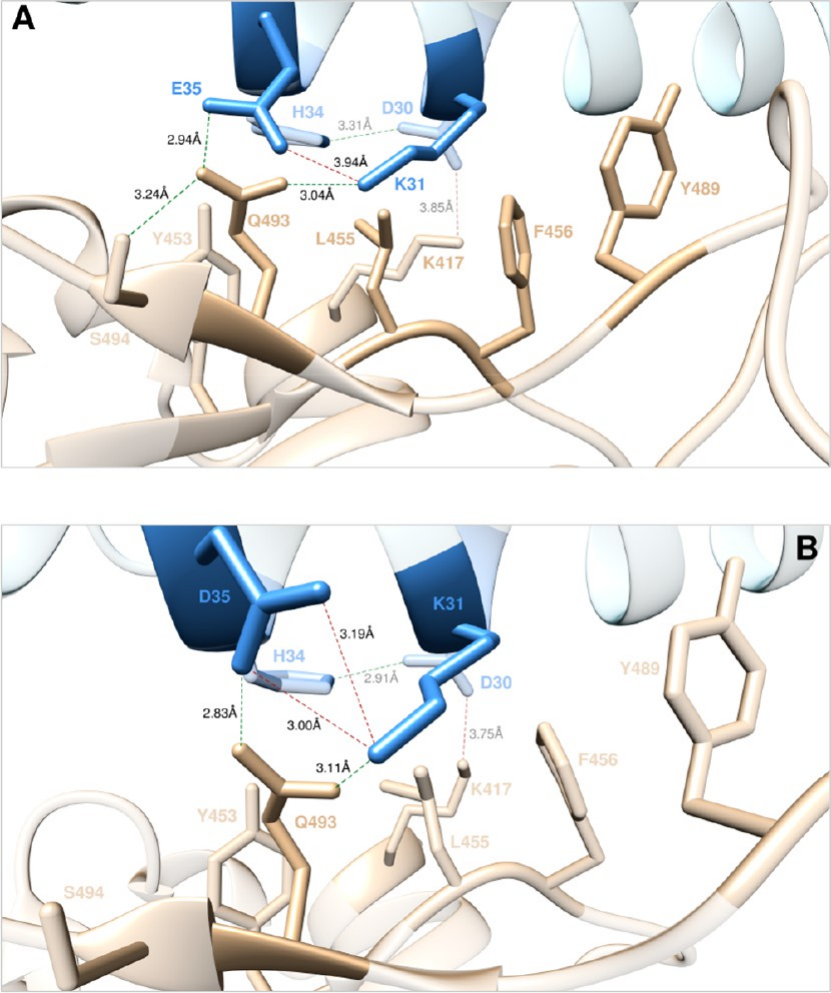
Main interactions involving the ACE2 wild-type residues K31 and E35 (A), and the corresponding D35 mutant (B) at the interface with S-RBD_CoV-2_ as obtained from the relevant equilibrated MD simulations. Images for all other K31 and E35 mutants are shown in Figures S9–S10 (see also Tables S11–S12 for details). Colors and other explanations are as in Figure 2.

**Figure 9:**
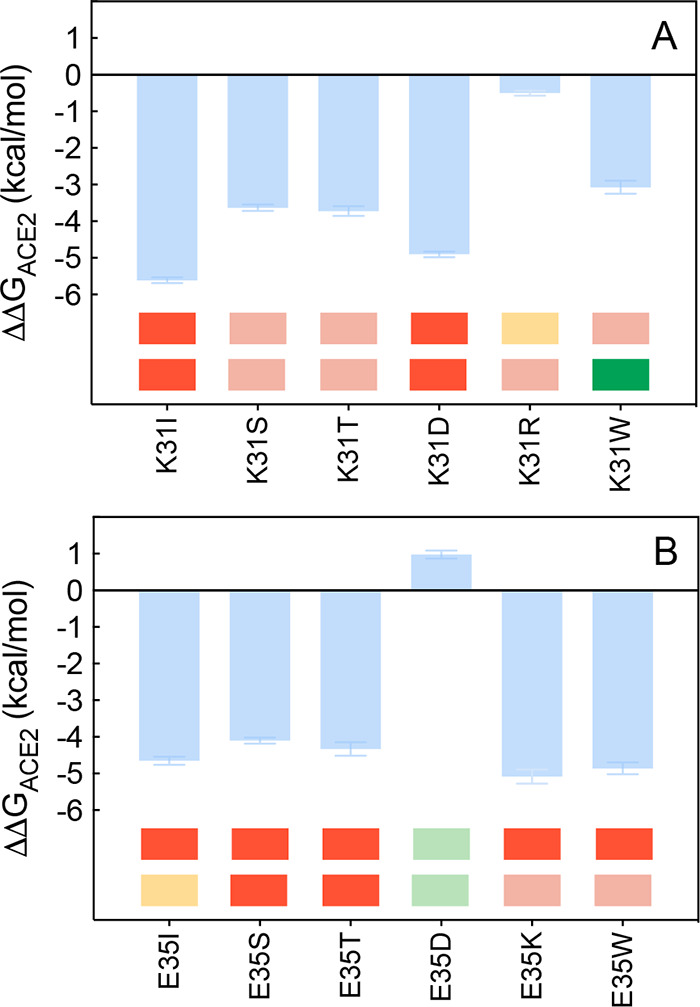
Change in binding free energy predicted by computational mutagenesis of the ACE2 residues K31 (A) and E35 (B) for the corresponding ACE2/S-RBD_CoV-2_ complexes. Colors and other explanations are as in Figure 3. The numerical values of ΔΔ*G*, all related energy terms, and all underlying intermolecular/intramolecular interactions are reported in Table S1, Figures S9–S10, and Tables S11–S12.

Our earlier CAS study highlighted the wild-type K31 as a hot-spot residue in the interaction between ACE2 and the receptor binding domain of the SARS-CoV-2 S-protein.^25^ Indeed, K31 establishes a topical internal SB with the side chain of E35 (3.94 ± 0.42 Å), which is further stabilized by (i) two permanent intermolecular HBs of both K31 and E35 with Q493 of S-RBD_CoV-2_ (3.04 ± 0.25 Å and 2.94 ± 0.19 Å, respectively), and (ii) extensive CIs with the side chains of the viral protein residues L455, F456, and Y489 (Figure 8A). As such, replacing ACE2 K31 with alanine in the corresponding protein/protein complex resulted in a substantial loss of binding free energy (ΔΔ*G*_ACE2_(K31A) = −4.85 ± 0.14 kcal/mol).^25^ In line with these previous findings, the current computational mutagenesis predicts robust destabilizing effects—involving not only the previously discussed interactions but also the related network of intra- and intermolecular contacts comprising ACE2 residues D30 and H34 and the S-RBD_CoV-2_ residues K417, Y453, and S494, Figures 8A–S9 and Table S11)—when K31 is replaced by I, S, T, D, R, and W, in almost complete agreement with the relevant experimental results^35^ (Figure 9A; for a discussion on K31W data, see Supporting Information).

As ACE2 residue E35 is strongly related to K31 (Figure 8A), it is not surprising that—as already predicted in the case of the E35A mutation, for which ΔΔ*G*_ACE2_(E35A) = −2.89 ± 0.15 kcal/mol^25^—replacing ACE2 E35 with each of the alternative residues considered also reflects in a moderate-to-strong interface disrupting behavior (Figure 9B, Table S1, Figure S10 and Table S12), with the exception of the substitution E35D, for which both *in silico* and *in vitro*(35) data anticipate an interface stabilizing contribution (Figure 9B). As seen in Figure 8B, in the presence of the shorter yet charge-preserving aspartic acid, the entire set of intra- and intermolecular interactions is preserved across the binding interface; moreover, similarly to what was already described above for the H34K mutant, D35 is further able to clip K31 *via* a stronger, bifurcated SB (3.00 ± 0.16 Å and 3.19 ± 0.20 Å, respectively; Figure 8B and Table S12), thereby increasing the affinity of the D35 ACE2 mutant for the viral protein (ΔΔ*G*_ACE2_(E35D) = +0.98 ± 0.11 kcal/mol, Figure 9B, and Table S1).

#### K353, D38, and Q42

**Figure 10:**
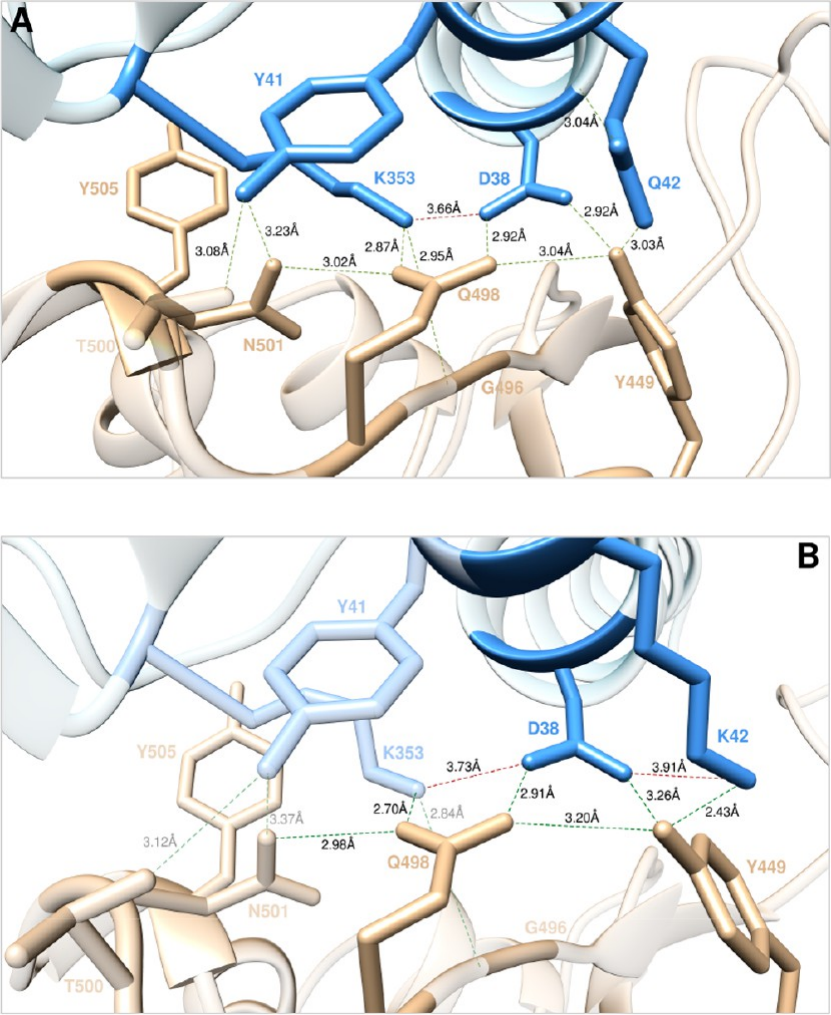
Main interactions involving the ACE2 wild-type residues K353, D38, and Q42 (A), and the corresponding K42 mutant (B) at the interface with S-RBD_CoV-2_ as obtained from the relevant equilibrated MD simulations. Images for all other K353, D38, and Q42 mutants are shown in Figures S11–S13 (see also Tables S13–S15 for details). Colors and other explanations as in Figure 2.

**Figure 11:**
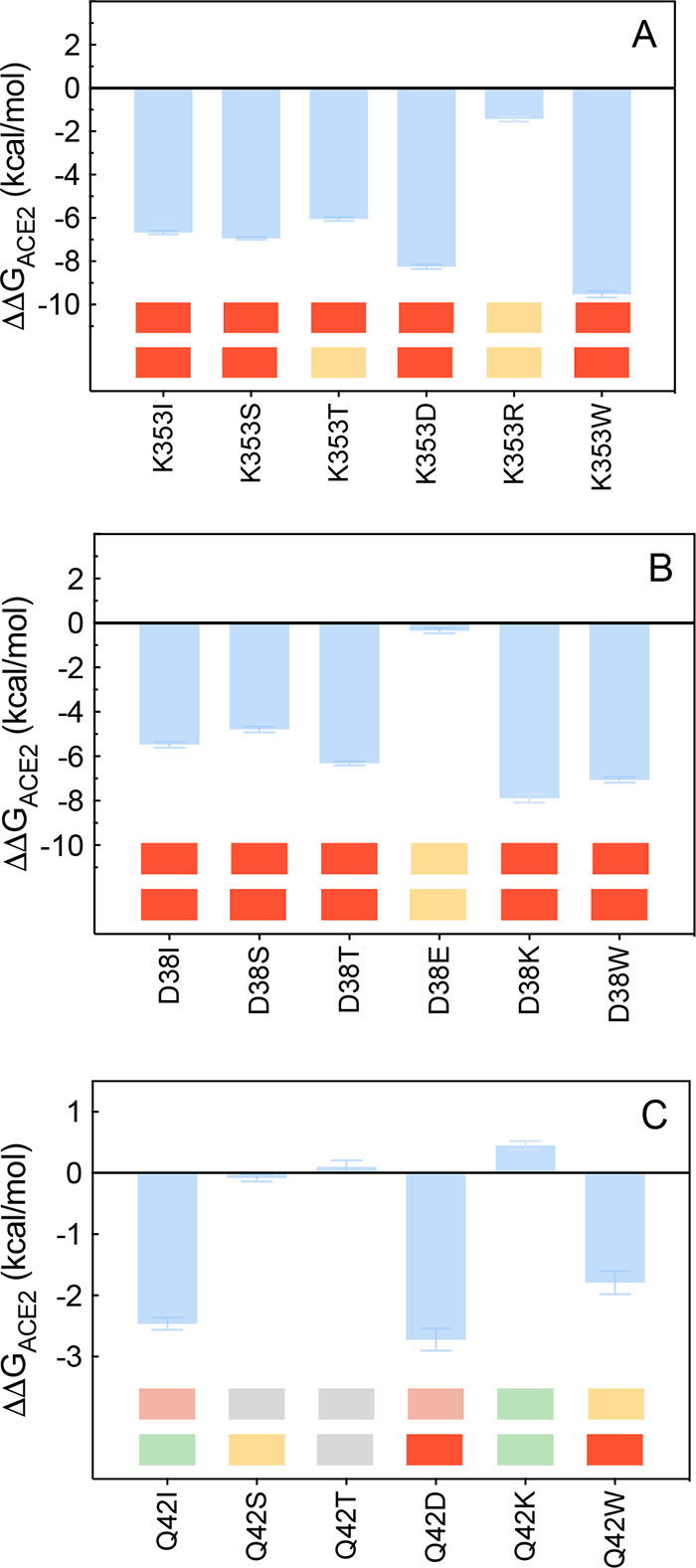
Change in binding free energy predicted by computational mutagenesis of the ACE2 residues K353 (A), D38 (B), and Q42 (C) for the corresponding ACE2/S-RBD_CoV-2_ complexes. Colors and other explanations are as in Figure 3. The numerical values of ΔΔ*G*, all related energy terms, and all underlying intermolecular/intramolecular interactions are reported in Table S1, Figures S11–13, and Tables S13–S15.

According to our original CAS study, another ACE lysine at the interface between the human receptor and the SARS-CoV-2 spike protein—K353—plays the role of hot spot residue in the formation of this protein/protein complex.^25^ Indeed, mutating K353 into alanine resulted in great affinity loss of the ACE2 K353A mutant for the viral RBD (ΔΔ*G*_ACE2_(K353A) = −7.19 ± 0.74 kcal/mol),^25^ which accounted for the abrogation of the underlying extensive system of interactions: the fundamental intermolecular charge-neutralizing SB with D38 (3.66 ± 0.39 Å), two permanent intermolecular HBs with the side chain/backbone of Q498 (2.87 ± 0.13 Å) and G496 (2.95 ± 0.21 Å), respectively, and CIs of polar and dispersive nature with the side chains of N501 and Y505 (on S-RBD_CoV-2_) and Y41 (on ACE2) (Figure 10A). Data from the actual computational and experimental^35^ mutagenesis confirm the relevance of this residue in the formation of the receptor/S-protein complex (Figure 11A, Table S1), with all considered mutant residues resulting in a strong interface destabilization caused by the drastic decrease of the relevant protein/protein contacts (Figure S11, Table S13). In the same way, the experimental^35^/computational mutation of ACE2 D38—the intramolecular SB partner of K353 further involved in three HBs (with Q498 (2.92 ± 0.19 Å) and Y449 (2.92 ± 0.20 Å) of S-RBD_CoV-2_ and with ACE2 Q42 (3.04 ± 0.18 Å)), and two CIs (with Y449 and G496) (Figure 10A)—into I, S, T, D, K or W also produces ACE2 isoforms with drastically lower affinity for its viral counterpart (Figure 11B, Table S1, Figure S12, and Table S14), confirming the hot-spot role for this residue predicted in our previous CAS study (ΔΔ*G*_ACE2_(D38A) = −5.11 ± 0.21 kcal/mol).^25^

Mutating Q42 on ACE2 into alanine *in silico* resulted in the moderated loss of binding affinity of the mutant receptor for the S-protein RBD (ΔΔ*G*_ACE2_(Q42A) = −2.19 ± 0.11 kcal/mol),^25^ mainly ascribable to the abrogation of the direct intermolecular HB of Q42 and the viral Y449 (3.03 ± 0.11) Å across the binding interface (Figure 10A). In line with this, both experiment^35^ and the present computational analysis support moderate interface destabilizing effects upon substitution of the wild-type asparagine with all other residues considered apart from K42, for which a stabilization of the relative protein/protein interface is reported (Figure 11C, Table S1) (for a discussion on Q42I and Q42S data, see Supporting Information). As shown in Figure 10B, according to our MD simulations the K42 ACE mutant isoform is able to fully preserve the strong interaction network characterizing the wild-type receptor/S-protein binding interface (Figure 10A, Table S15); in addition, the long and positively charged lysine chain of K42 established a further intramolecular SB with D38 (3.91 ± 0.27 Å, Figure 10B), ultimately resulting in a small gain of affinity of the K42 mutant receptor for the S-RBD_CoV-2_ (ΔΔ*G*_ACE2_(Q42K) = +0.45 ± 0.07 kcal/mol, Figure 11C, Table S1).

#### Y41, D355, and R357

**Figure 12:**
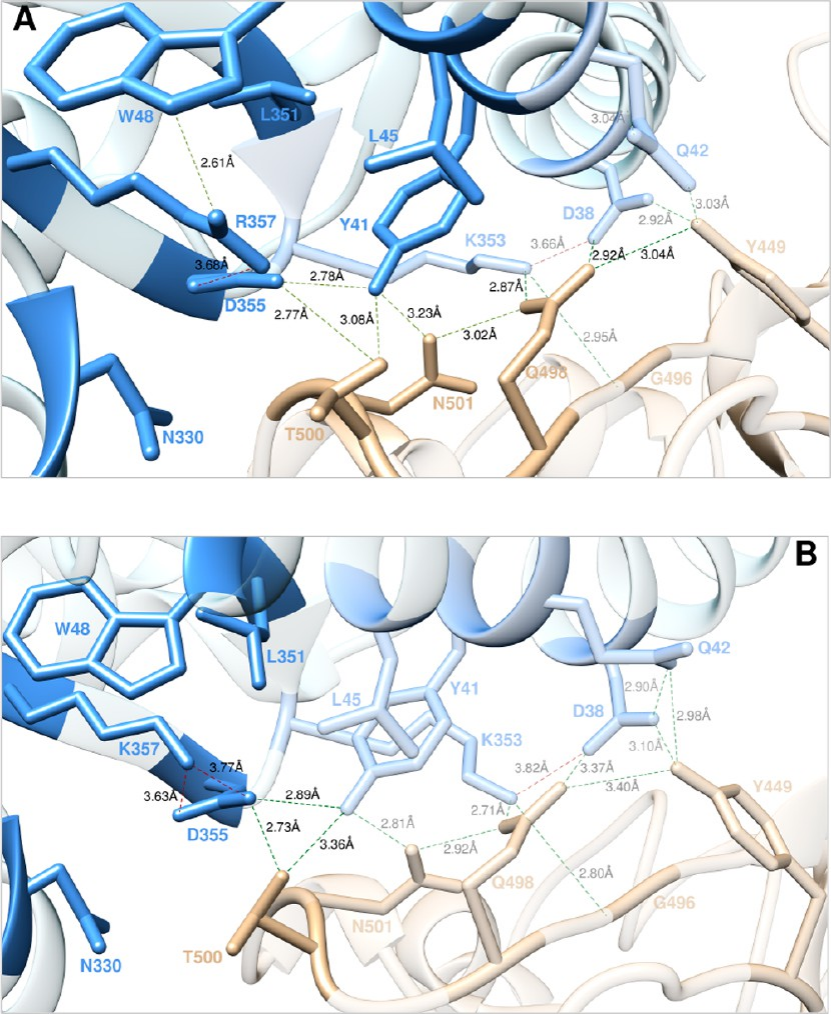
Main interactions involving the ACE2 wild-type residues Y41, D355, and R357 (A), and the corresponding R357 K mutant (B) at the interface with S-RBD_CoV-2_ as obtained from the relevant equilibrated MD simulations. Images for all other Y41, D355, and R357 mutants are shown in Figures S14–S16 (see also Tables S16–S18 for details). Colors and other explanations as in Figure 2.

**Figure 13:**
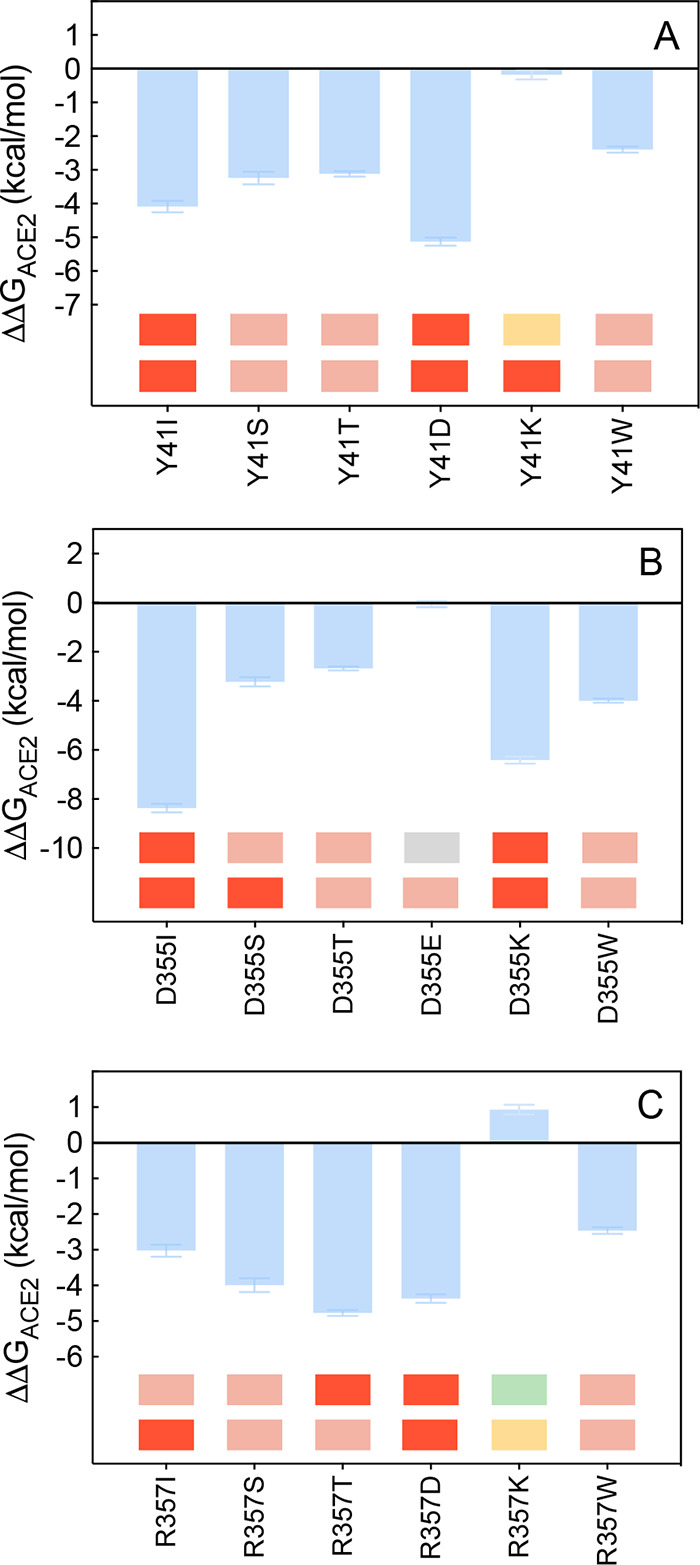
Change in binding free energy predicted by computational mutagenesis of the ACE2 residues Y41 (A), D355 (B), and R357 (C) for the corresponding ACE2/S-RBD_CoV-2_ complexes. Colors and other explanations as in Figure 3. The numerical values of ΔΔ*G*, all related energy terms, and all underlying intermolecular/intramolecular interactions are reported in Table S1, Figures S14–16, and Tables S16–S18.

Y41 on ACE2 α-helix 1 is another predicted hot spot at the binding interface between the human receptor and the viral S-protein RBD, since mutating this tyrosine into alanine yielded a substantial loss in the relevant binding free energy (ΔΔ*G*_ACE2_(Y41A) = −4.43 ± 0.33 kcal/mol).^25^ As shown in Figure 12A, ACE2 Y41 is instrumental in the establishment of a complex network of inter- and intramolecular interactions across the protein–protein binding interface which includes, besides the two direct HBs with the viral protein residues T500 (3.08 ± 0.23 Å) and N501 (3.23 ± 0.22 Å) and the CIs with the side chains of Q498 (intermolecular) and K353 (intramolecular) described above, an important HB with ACE D355 (2.78 ± 0.15 Å) and further favorable CIs with the side chains of L45 and L351 (Figure 12A). All these interactions concur in shaping the binding interface for other, fundamental sets of binding contacts involving ACE2 K31, D38, and Q42 with the side chains of N501, Q498, and Y449 on S-RBD_CoV-2_ (Figures 10 and 11). As could be anticipated, computational and experimental^35^ mutagenesis of Y41 into the selected residues both provoke interface destabilizing effects in all cases (Figures 13A and S14, Tables S1 and S16). With regard to the wild-type D355 residue on ACE2, the main role of this aspartic acid is to naturalize the positive charge of the adjacent R357 *via* an internal SB (3.68 ± 0.19 Å), while concomitantly H-bonding the viral threonine 500 (2.77 ± 0.16 Å) and ACE2 Y41, as discussed a few lines previously (Figure 12A). Abrogation of these interactions when D355 was replaced by alanine *via* CAS resulted in the important loss of affinity of the A355 mutant receptor for the viral protein, as quantified by ΔΔ*G*_ACE2_(D355A) = −3.18 ± 0.20 kcal/mol.^25^ In analogy with Y41, also for this residue both *in silico* and *in vitro*(35) data agree on interface destabilization in the presence of I, S, T, K, and W, again with a small deviation between experiment and prediction concerning the homologous substitution D355E (mildly destabilizing vs neutral effect, respectively) (Figures 13B and S15, Table S1 and S17; for a discussion on D355E data see Supporting Information).

Previous CAS data anticipated a negative effect in replacing the ACE2 R357 with alanine (ΔΔ*G*_ACE2_(R357A) = −3.32 ± 0.25 kcal/mol).^25^ In line with this prediction, the current computational and experimental^35^ mutagenesis data confirm human receptor/S-RBD_CoV-2_ interface destabilizing effects when replacing the wild-type arginine with all alternative residues considered except for the R357 K mutation, for which *in silico* data support a weak protein/protein stabilizing action (ΔΔ*G*_ACE2_(R357 K) = +0.93 ± 0.14 kcal/mol, Figure 13C, Table S1, Figures S16, and Table S18). As seen by comparing panels A and B in Figure 12, in the presence of the mutant K357 all major inter- and intramolecular interactions detected in the wild-type complex are preserved; moreover, at variance with the native R357, the shorter K393 side chain engages D355 in a bifurcated intramolecular salt bridge (3.63 ± 0.23 Å and 3.77 ± 0.18 Å, respectively), while ACE2 D38 is stabilized by a more effective side chain-side chain intramolecular HB with Q42 (3.63 ± 0.26 Å) (Figure 12B, Table S18).

#### E37 and R393

**Figure 14:**
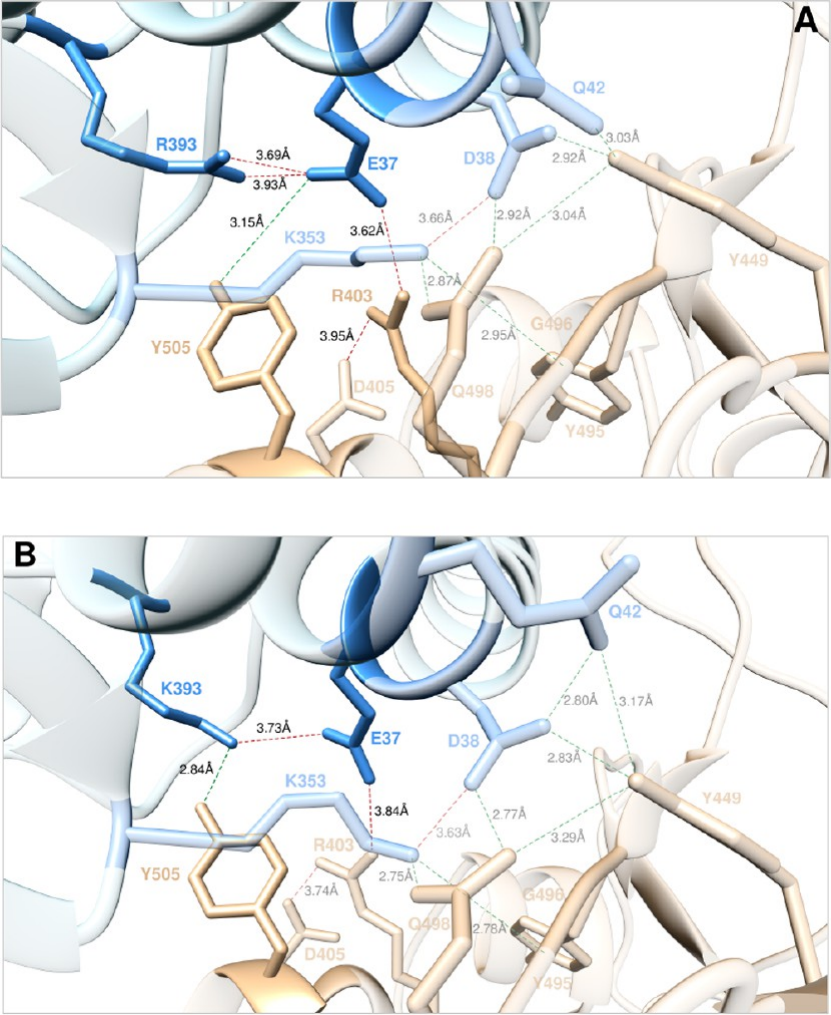
Main interactions involving the ACE2 wild-type residues E37 and R393 (A) and the corresponding R393 K mutant (B) at the interface with S-RBD_CoV-2_ as obtained from the relevant equilibrated MD simulations. Images for all other E37 and R393 mutants are shown in Figures S17–S18 (see also Tables S19–S20 for details). Colors and other explanations are as in Figure 2.

**Figure 15:**
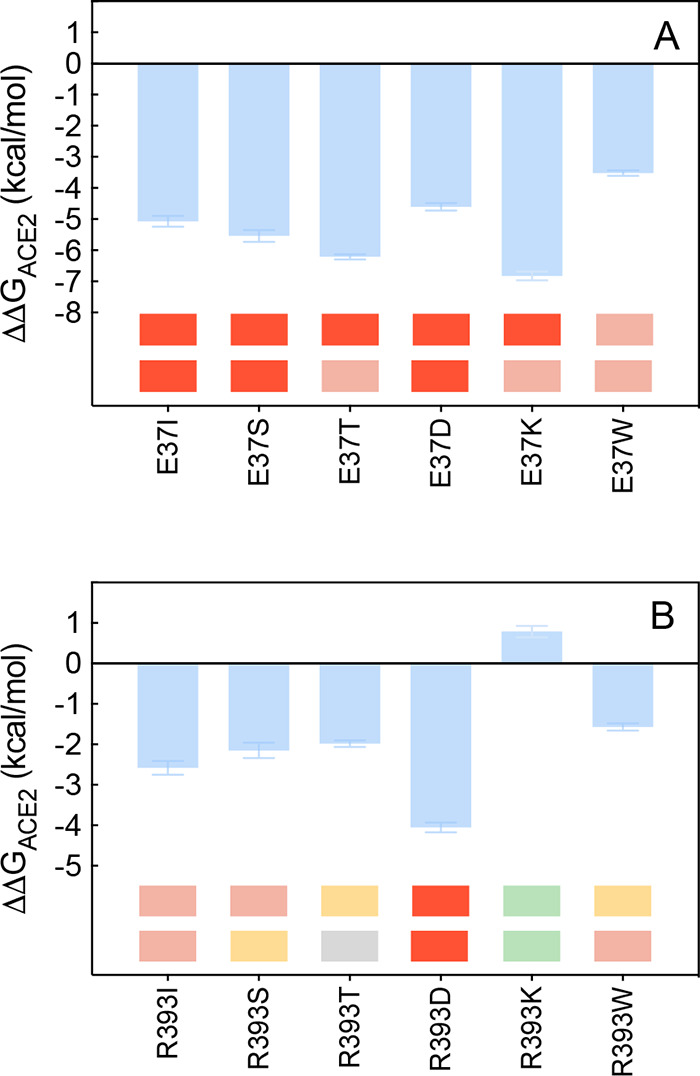
Change in binding free energy predicted by computational mutagenesis of the ACE2 residues E37 (A) and R393 (B) for the corresponding ACE2/S-RBD_CoV-2_ complexes. Colors and other explanations as in Figure 3. The numerical values of ΔΔ*G*, all related energy terms, and all underlying intermolecular/intramolecular interactions are reported in Table S1, Figures S17–S18, and Tables S19–S20.

According to the results of our previous study,^25^ the ACE2 wild-type residue E37 is another hot-spot at the S-RBD_CoV-2_/ACE2 complex, where it is involved in two interface stabilizing contacts: a SB with R403 (3.62 ± 0.39 Å) and an HB with the side chain of Y505 (3.15 ± 0.24 Å); moreover, it plays an important structural role by anchoring ACE2 R393 *via* an internal bifurcated SB (3.93 ± 0.38 Å and 3.69 ± 0.27 Å, respectively) (Figure 14A). As such, the corresponding variation in binding free energy between the wild-type and mutant receptor carrying an alanine residue at the same position (E37A) in complex with the viral S-protein was estimated to be quite significant (ΔΔ*G*_ACE2_(E37A) = −5.12 ± 0.22 kcal/mol).^25^ The predicted importance of ACE2 E37 at this protein/protein binding region is consistently confirmed by the present computational mutagenesis results and the relevant experimental data:^35^ both techniques indeed reveal strong-to-very strong interface destabilizing effects when this glutamic acid is change into each of the alternative residues considered (Figure 15A, Table S1, Figures S17–S18, and Tables S19–S20).

Finally, as anticipated by the relevant CAS data (ΔΔ*G*_ACE2_(R393A) = −2.33 ± 0.19 kcal/mol),^25^ replacing either *in silico* or *in vitro*(35) the ACE2 wild-type arginine 393 with any of the selected residues reflects into mild-to-strong destabilizing effects of the relevant receptor/S-protein binding interface, with the somewhat expected opposite outcome for the homologous substitution R393 K (Figure 15B, Tables S1 and S20; for a discussion on R393T data, see Supporting Information). From the equilibrated MD snapshots portrayed in Figure 14, it is apparent that the R393 K mutant ACE2 isoform (Figure 14B) is able to engage the SARS-CoV-2 RBD in the same interaction network established by the wild-type receptor (Figure 14A, Table S20) yet with an overall compaction of the protein/protein interface; this, in turn, ultimately reflects in a moderate increase of the K393 ACE2 mutant affinity for the viral protein (ΔΔ*G*_ACE2_(R393 K) = +0.79 ± 0.14 kcal/mol, Figure 15B, and Table S1).

### Analysis of the SARS-CoV-2 S-RBD Residues at the Binding Interface with ACE2

#### Y449, Y453, and T500

**Figure 16:**
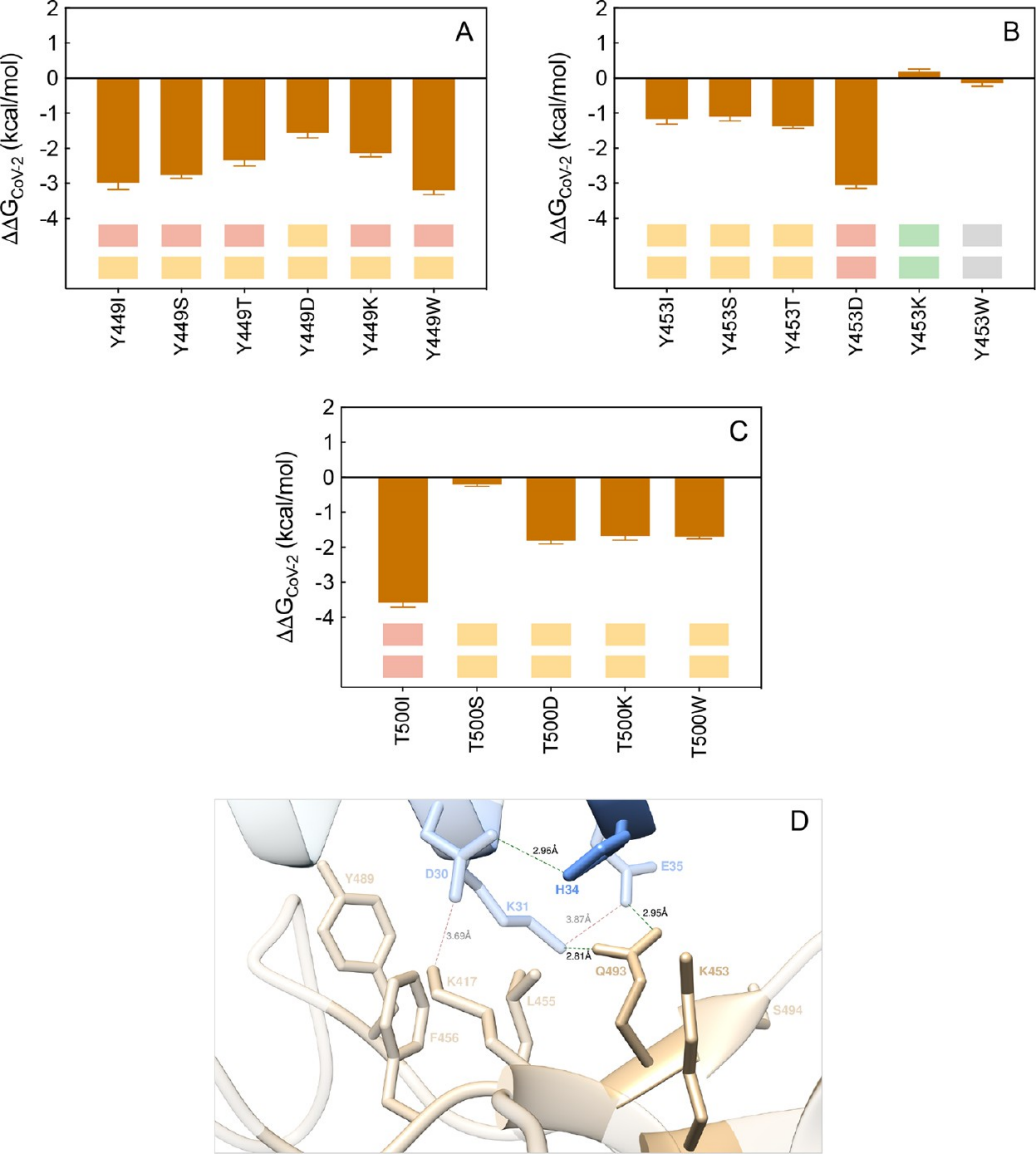
Change in binding free energy (ΔΔ*G* = Δ*G*_WILD-TYPE_ – Δ*G*_MUTANT_) predicted by computational mutagenesis of the S-RBD_CoV-2_ residues Y449 (A), Y453 (B), and T500 (C) for the corresponding ACE2/S-RBD_CoV-2_ complexes. In each protein–protein assembly, the wild-type residue is replaced by I, S, T, D/E, K/R, and W. Negative/positive ΔΔ*G* values indicate unfavorable/favorable substitutions for the mutant residue in the relevant position, respectively. The numerical values of ΔΔ*G*, all related energy terms, and all underlying intermolecular/intramolecular interactions are reported in Table S2, Figures S19–S21, and Tables S21–S23. The colored boxes below each bar in the graphs show the qualitative comparison between *in silico* predicted (upper row) and experimental^36^ (lower row) destabilizing/stabilizing effects of the corresponding mutation on the ACE2/SARS-CoV-2 S-protein complex. Color legend: light yellow, mildly destabilizing mutations; light red, destabilizing mutations; red, highly destabilizing mutations; gray, neutral mutation; light green, stabilizing mutations; green, highly stabilizing mutations. (D) Main interactions involving the S-RBD_CoV-2_ Y453K mutant at the interface with ACE2 as obtained from the relevant equilibrated MD simulations. Colors and other explanations as in Figure 2. Images for all other Y449, Y453, and T500 mutants are shown in Figures S19–S21 (see also Tables S21–23 for details).

According to our previous CAS investigation,^25^ the wild-type S-RBD_CoV-2_ residue Y449 engages the side chains of ACE2 residues Q42 and D38 in two HBs and CIs across the binding interface (3.03 ± 0.11 Å and 2.92 ± 0.20 Å, respectively) (Figure 10A). *Via* an internal HB Y449 anchoring the side chain of Q498 (3.04 ± 0.18 Å), a viral protein residue fundamental in shaping all other intra- and intermolecular interactions of this protein/protein binding region (Figure 10A). In line with the value predicted for the Y449A mutant (ΔΔ*G*_CoV-2_(Y449A) = −3.21 ± 0.31 kcal/mol),^25^ the actual computational and experimental^36^ results both report interface destabilizing effects in all ACE2/mutant S-RBD_CoV-2_ complexes considered (Figure 16A, Table S2, Figure S19, and Table S21).

For the proximal Y453 S-protein residue, the original CAS study predicted only a small change in affinity of the Y453A S-RBD_CoV-2_ mutant for ACE2 (ΔΔ*G*_CoV-2_(Y453A) = −0.79 ± 0.30 kcal/mol),^25^ in line with the limited role played by this residue in the wild-type complex (a polar intermolecular interaction with ACE2 H34 and one intramolecular CI with Q493, Figure 4A). The actual *in silico*/experimental^36^ data also commonly describe neutral-to-mildly destabilizing effects for the W453, I453, S453, and T453 mutants, respectively, a stronger negative influence on the protein/protein complex stability in the presence of Y453D substitution and, quite interestingly, a small increase in affinity of the Y453K mutant S-RBD_CoV-2_ for the human receptor (Figure 16 panels B and D, Table S2, Figure S20, and Table S22). The Y453K variant of the S-protein RBD indeed engages the same CIs of its wild-type isomer yet in a somewhat more efficient manner, resulting in a slight compaction of the corresponding binding interface region (Figure 16D, Table S22). In addition, the related network of interactions seen in the wild-type complex is practically unchanged (see Figure 4A, Figure 16D, and Table S22), ultimately resulting in slightly positive predicted variation of the corresponding protein/protein binding free energy (ΔΔ*G*_CoV-2_(Y453K) = +0.19 ± 0.07 kcal/mol, Figure 16B and Table S2).

T500 of S-RBD_CoV-2_ is located at a tip of a flexible loop in the RBD of the SARS-CoV-2 S-protein, which includes two other residues—Q498 and N501—involved in receptor binding (Figure 12A). In the wild-type ACE2/S-RBD_CoV-2_ complex, this threonine establishes two strong intermolecular HBs with the side chains of ACE2 Y41 and D355 (3.08 ± 0.23 Å and 2.77 ± 0.16 Å, respectively), and CIs of polar and dispersive nature with the side chains of R357 and N330 across the protein/protein interface^25^ (Figures 12A). Thus, when this polar amino acid is replaced by an hydrophobic residue such as alanine or isoleucine, a significant loss in binding free energy is predicted (ΔΔ*G*_CoV-2_(T500A) = −4.17 ± 0.36 kcal/mol^25^ and ΔΔ*G*_CoV-2_(T500I) = −3.58 ± 0.13 kcal/mol, respectively) and experimentally observed^36^ (Figure 16C and Table S2). In both virtual and real experiments,^36^ all other substitutions considered (S/D/K/W) also reflect in a loss of affinity of the corresponding mutated viral protein for the receptor, although to a minor extent (Figure 16C, Table S2). The analysis of the corresponding MD trajectories (Figure S21 and Table S23) reveals that the combination of loop mobility and the presence of polar/charged groups in the replaced amino acids compensates—at least in part—for the loss of the native interactions and contributes in partially preserving the shape of the relevant protein/protein binding interface.

#### N487, Y489, and Y505

**Figure 17:**
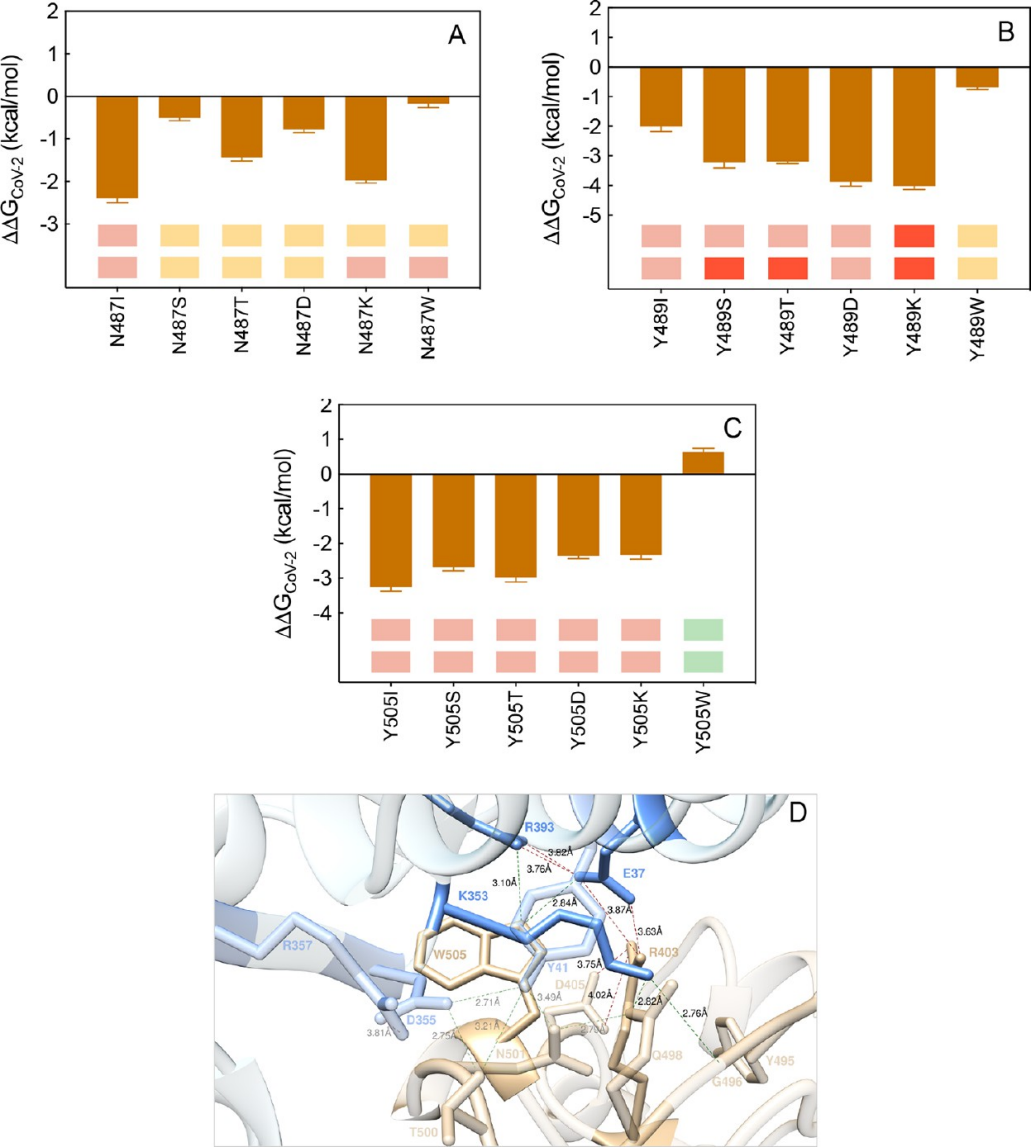
Change in binding free energy predicted by computational mutagenesis of the S-RBD_CoV-2_ residues N487 (A), Y489 (B), and Y505 (C) for the corresponding ACE2/S-RBD_CoV-2_ complexes. The numerical values of ΔΔ*G*, all related energy terms, and all underlying intermolecular/intramolecular interactions are reported in Table S2, Figures S22–S24, and Tables S24–S26. (D) Main interactions involving the S-RBD_CoV-2_ Y505W mutant at the interface with ACE2 as obtained from the relevant equilibrated MD simulations. Images for all other N487, Y489, and Y505 mutants are shown in Figures S22–S24 (see also Tables S24–S26 for details). Colors and other explanations are as in Figure 2.

N487 is a S-RBD_CoV-2_ residue that directly engages only two stabilizing intermolecular HBs with the side chains of ACE2 Q24 and Y83 (3.03 ± 0.18 Å and 2.88 ± 0.17 Å, respectively, Figure 2A). Both of these HBs contribute in cementing the set of CIs developed within the hydrophobic patch lined by the side chains of F28, L29, Q76, L79, M82, Y83, and L97 on ACE2, and of F486 and Y489 on the viral protein (Figure 6A). Consistent with this, the absence of these two HBs in the N487A or N487I S-RBD_CoV-2_ mutants reflects in a non-negligible interface destabilization, quantified by the corresponding variation of the binding free energies (ΔΔ*G*_CoV-2_(N487A) = −2.25 ± 0.35 kcal/mol,^25^ and ΔΔ*G*_CoV-2_(N487I) = −2.39 ± 0.11 kcal/mol, respectively, Table S2), in agreement with experiment^36^ (Figure 17A). However, these HBs (one or both) are detected again in all alternative mutated protein/protein complex MD trajectories, *via* the relevant HB donor/acceptor (S487, T487, and W487) or charged moieties (D487 and K487) (Figure S22 and Table S24). The current mutagenesis results, in agreement with *in vitro* data,^36^ confirm the mildly perturbing effects of all other S-RBD_CoV-2_ mutant isoforms on the relative protein/protein binding interface (Figure 17A, Table S2, Figure S22, and Table S24). Y489 in S-RBD_CoV-2_ contributes to human receptor binding by contacting the side chains of ACE2 residues Q24, Y83, T27, and K31 *via* van der Waals/hydrophobic and polar intermolecular interactions (Figure 2C). Accordingly, mutating Y489 into alanine and isoleucine quantified the strength of this network as ΔΔ*G*_CoV-2_(Y489A) = −2.96 ± 0.33 kcal/mol^25^ and ΔΔ*G*_CoV-2_(Y489I) = −2.01 ± 0.17 kcal/mol (Table S2), respectively. However, when changing the native tyrosine into S/T/D or K, stronger interface-destabilizing effects are predicted/observed^36^ in all cases (Figure 17B), as a consequence of a more intensive perturbation of this CI network (Table S2, Figure S23, and Table S25).

Tyrosine at position 505 of the S-RBD_CoV-2_ is a residue that—besides directly interacting with ACE2 (through a HB with E37 (3.15 ± 0.24 Å) and two CIs with R393 and K353) and establishing an intramolecular π-cation (π/c) with the guanidinium group of R403 (Figure 14A)—plays an important role in reinforcing the binding interface contacts involving topical residues such as Y41 and D355 on the human protein and T500, N501, and Q498 on the viral counterpart. In agreement with this, all considered Y505 mutants (including the previously investigated Y505A, for which ΔΔ*G*_CoV-2_(Y505A) = −3.27 ± 0.31 kcal/mol^25^) considerably reduce the binding affinity of S-RBD_CoV-2_ for ACE2 with the exception of the Y505K variant, for which an interface-stabilizing effect is both predicted and observed^36^ (Figure 17C and Table S2). As shown in panel D of Figure 17, the S-RBD_CoV-2_ Y505 variant preserves the full network of intra- and intramolecular interactions at the corresponding protein/protein interface, with the addition of a new, stabilizing intermolecular HB across the binding interface between the side chains of W505 and ACE2 R393 (3.10 ± 0.26 Å) (Figure 17D and Table S26). In energetic terms, this translates to a slightly more favorable ΔΔ*G* value for the W505 mutant with respect to the wild-type S-RBD_CoV-2_ Y505 (ΔΔ*G*_CoV-2_(Y505W) = +0.64 ± 0.19 kcal/mol, Figure 17C, and Table S2).

#### L455, F456, and F486

**Figure 18:**
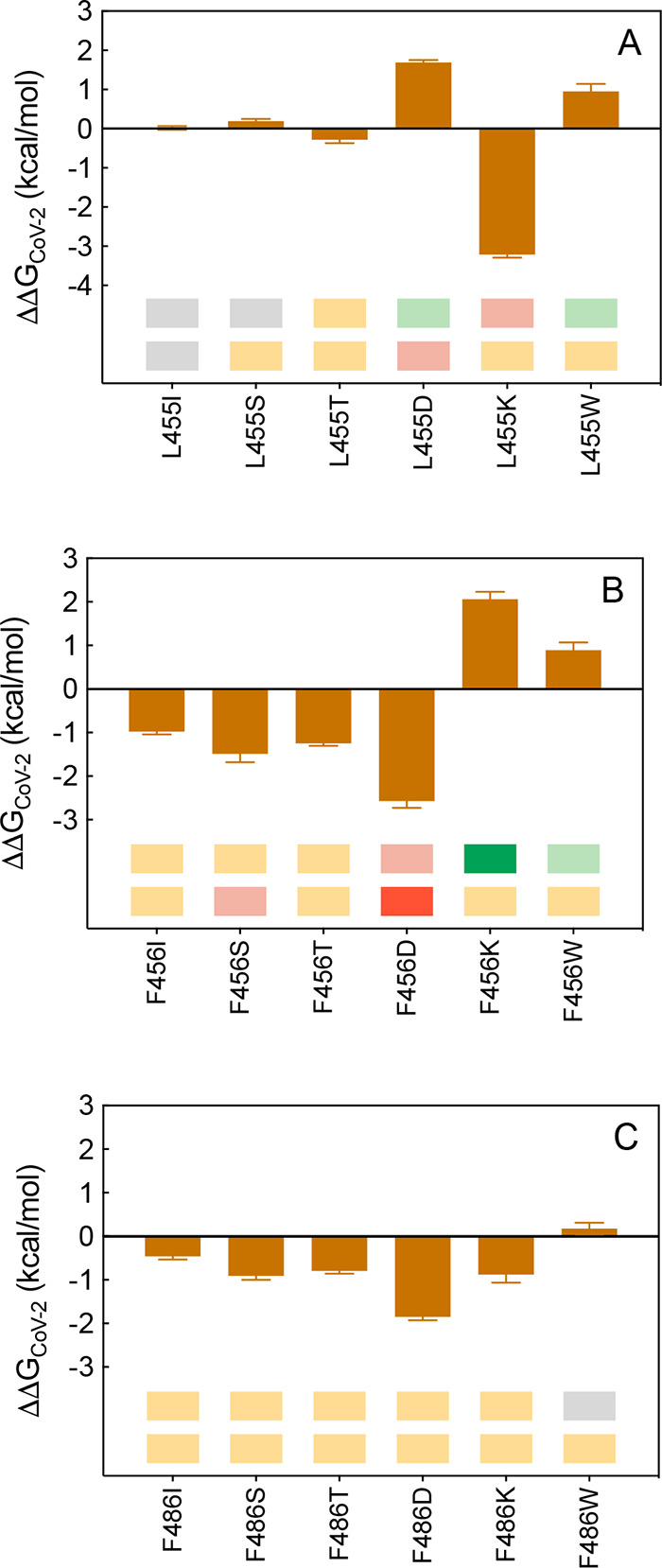
Change in binding free energy predicted by computational mutagenesis of the S-RBD_CoV-2_ residues L455 (A), F456 (B), and F486 (C) for the corresponding ACE2/S-RBD_CoV-2_ complexes. The numerical values of ΔΔ*G*, all related energy terms, and all underlying intermolecular/intramolecular interactions are reported in Table S2, Figures S25–S27, and Tables S27–S29.

S-RBD_CoV-2_ L455 occupies a cavity lined by the side chains of ACE2 D30, K31, and H34, with which it engages moderately stabilizing CIs (Figures 4A and 8A). As such, abrogating these interactions by replacing L455 with alanine resulted in a limited decrement of the corresponding free energy variation (ΔΔ*G*_CoV-2_(L455A) = −1.21 ± 0.32 kcal/mol).^25^ While both experimental^36^ and the present computational mutagenesis data report neutral-to-mild interface destabilizing effects for the L455I/S/T/K mutants, *in vitro*/*in silico* data diverge when the two remaining mutations (*i.e*., L455D and L455W) are concerned (Figure 18A and Table S2; for a discussion on L455S data, see Supporting Information).

**Figure 19:**
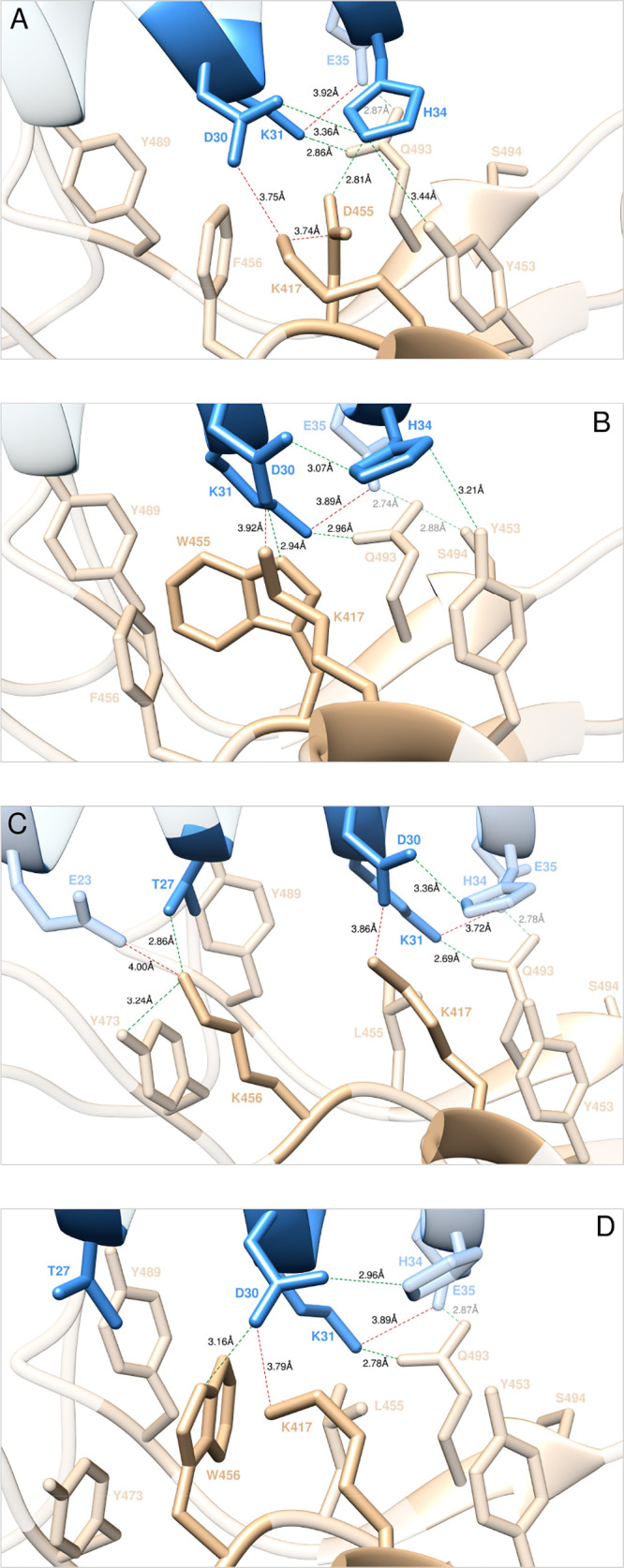
Main interactions involving the S-RBD_CoV-2_ D455 (A), W455 (B), K456 (C), and W456 (D) mutants at the interface with ACE2 as obtained from the relevant equilibrated MD simulations. Images for all other L455, F456, and F486 mutants are shown in Figures S25–S27 (see also Tables S27–S29 for details). Colors and other explanations as in Figure 2.

In particular, in the ACE2/(L455D)S-RBD_CoV-2_ complex, besides all wild-type inter- and intramolecular interaction, MD simulations reveal the formation of three further stabilizing contacts (Figure 19A, Table S27). Specifically, the viral mutant residue D455 engages ACE H34 in a persistent HB (2.81 ± 0.15 Å), thereby allowing H34 to interact with the viral tyrosine at position 453 *via* another HB across the binding interface (3.44 ± 0.22 Å) (Figure 19A, Table S27). In addition, the negatively charged side chain of D455 is able to form a stable intramolecular salt bridge with K417 (3.74 ± 0.29 Å), so that the overall affinity of the D455 isoform of the S-RBD_CoV-2_ for the human receptor is higher than that of the wild-type S-protein (ΔΔ*G*_CoV-2_(L455D) = +1.69 ± 0.06 kcal/mol, Figure 18A, and Table S2). In the case of the W455 mutant, again all wild-type interactions are preserved according to the present modeling (Figure 19B, Table S27); moreover, two extra HBs are formed across the corresponding protein/protein binding region, involving the side chains of W455 and D30 (2.94 ± 0.14 Å) and of Y453 and H34 (3.21 ± 0.26 Å), respectively (Figure 19B, Table S27). In line, an increase in affinity of the W455 viral mutant RBD for ACE2 is predicted (ΔΔ*G*_CoV-2_(L455W) = +0.95 ± 0.19 kcal/mol, Figure 18, Table S2).

F456 of S-RBD_CoV-2_ does not form direct intermolecular HBs or SBs with any ACE2 residue; however, it provides three CIs across the binding interface with the side chains of ACE2 T27, D30, and K31. In addition, F456 engages an important stabilizing intramolecular π/*c* interaction with K417 (thereby assisting this lysine in salt-bridging ACE2 D30), and an internal CI with the side chain of the viral Y473 (Figures 4A and 8A). The previous CAS study yielded a value of ΔΔ*G*_CoV-2_(F456A) = −1.99 ± 0.28 kcal/mol, in line with the abrogation of the underlying network of interactions.^25^ In the present study, the replacement of the wild-type S-RBD_CoV-2_ F456 with I, S, T, and D results in a negative effect on the binding between ACE2 and the SARS-CoV-2 RBD, in agreement with the relevant experimental findings^36^ (Figure 18B, Table S2). On the other hand, the computational data for the two remaining mutants (K456 and W456) predict interface stabilization, while *in vitro* data support mildly negative interfering effects (Figure 18B, Table S2). In the former case, K456 provides further inter- and intramolecular stabilizing interactions to the relative protein/protein complex, as this mutant lysine engages ACE2 T27 and E23 in an HB (2.86 ± 0.10 Å) and a SB (4.00 ± 0.24 Å), respectively, across the binding interface and is further stabilized *via* an internal HB (3.24 ± 0.19 Å) with the side chain of Y473 (Figure 19C, Table S28). Thus, the predicted relative variation of the binding free energy is positive and equal to ΔΔ*G*_CoV-2_(F456K) = +2.06 ± 0.17 kcal/mol (Figure 18B, Table S2). For the W456 mutant, the preservation of the wild-type interaction network coupled with the additional, stable intermolecular HB between the mutant residue and the side chain of ACE2 D30 (3.16 ± 0.13 Å) leads to a slight stabilization of the human receptor/viral protein, as quantified by the relevant value of ΔΔ*G*_CoV-2_(F456W) = +0.89 ± 0.18 kcal/mol (Figure 18B, Table S2).

S-RBD_CoV-2_ F486 plays a role utterly analogous to that described above for F456 in stabilizing the protein/protein binding interface by exchanging 3 intermolecular CIs with ACE2 residues L79, M82, and Y83 itself (Figure 6A). When this aromatic residue was mutated into alanine, the related variation of the binding free energy was predicted to be ΔΔ*G*_CoV-2_(F486A) = −2.13 ± 0.32 kcal/mol.^25^ The present calculations and the corresponding experimental data^36^ both predict a weak interface destabilizing effect when this residue is mutated into all residues considered, as shown in Figure 18C (see also Table 2, Figure S27, and Table S29; for a discussion on F486W data, see Supporting Information).

#### Q493 and Q498

**Figure 20:**
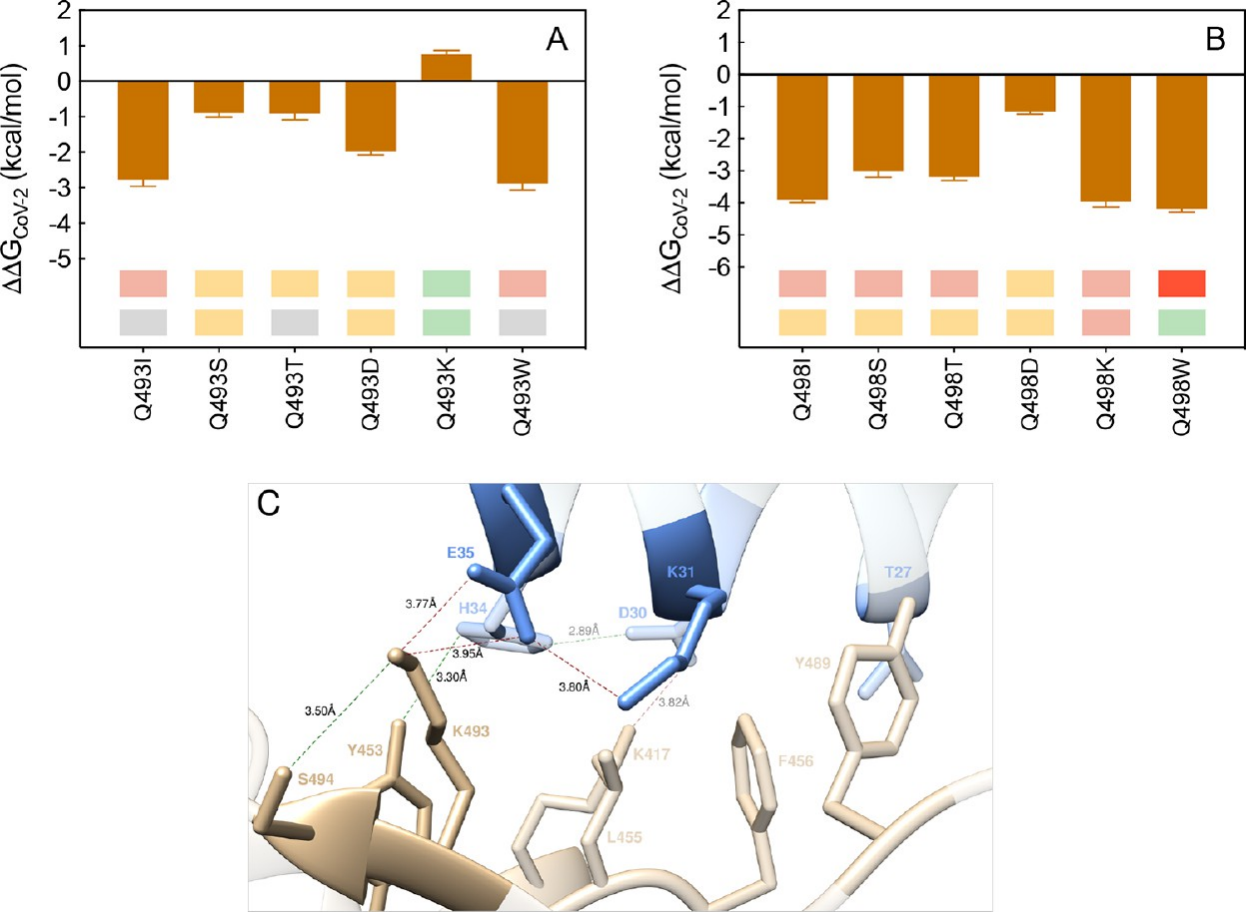
Change in binding free energy predicted by computational mutagenesis of the S-RBD_CoV-2_ residues Q493 (A) and Q498 (B) for the corresponding ACE2/S-RBD_CoV-2_ complexes. The numerical values of ΔΔ*G*, all related energy terms, and all underlying intermolecular/intramolecular interactions are reported in Table S2, Figures S27–S28, and Tables S30–S31. (C) Main interactions involving the S-RBD_CoV-2_ Q493K mutant at the interface with ACE2 as obtained from the relevant equilibrated MD simulations. Images for all other Q493 and Q498 mutants are shown in Figures S27–S28 (see also Tables S29–S30 for details). Colors and other explanations as in Figure 2.

The polar glutamine residue located at position 493 position along the SARS-CoV-2 S-protein sequence engages the side chains of ACE2 K31 and E35 in two intermolecular SBs (3.04 ± 0.25 Å and 2.94 ± 0.19 Å, respectively), the −OH moiety of S494 in an internal HB (3.24 ± 0.21 Å), and the side chain of the viral Y453 in van der Waals contacts (Figures 4A and 8A). Abrogation of these interactions in the Q493A mutant by CAS predicted a ΔΔ*G*_CoV-2_(Q493A) = −3.15 ± 0.29 kcal/mol.^25^ On the other hand, exchanging Q493 for I/S/T/D/K/W both computationally and experimentally^36^ leads to a more variegated free energy difference pattern (Figure 20A, Table S2). In particular, both techniques mostly report interface neutral-to-disrupting effects for all residues except for the Q493K variant, for which an interface stabilizing effect is jointly determined (Figure 20A, Table S2, Figure S28, and Table S30; for a discussion on Q493I, Q493T, and Q493W data, see Supporting Information). In detail, the analysis of the MD simulation of the K493 S-RBD_CoV-2_ mutant bound to ACE2 shows that all interactions seen in the wild-type complex are preserved (Figure 20C, Table S30); in addition, in the presence of K493 the single SB with E35 become bifurcated (3.77 ± 0.21 Å and 3.95 ± 0.17 Å, respectively), and an intermolecular HB between Y453 on the S-protein and H34 on ACE2 replaces the wild-type polar interaction (Figure 20C, Table S30). Thus, the variation in binding free energy is slight favorable to the mutant isoform (ΔΔ*G*_CoV-2_(Q493K) = +0.76 ± 0.11 kcal/mol, Figure 20A, and Table S2), in agreement with the experimental evidence.^36^

Our previous computational efforts have highlighted Q498 as a viral protein/receptor binding hot spot,^25^ since it was seen to play a major role in shaping the relevant region of the protein/protein binding interface. Precisely, Q498 has anchored ACE2 D38 and K353 in two topical intermolecular HBs (2.92 ± 0.19 Å and 2.87 ± 0.13 Å, respectively) along with favorable intermolecular CIs with the side chains of Q42 and Y41; moreover, the same glutamine internally H-binds N501 (3.02 ± 0.18 Å) and Y449 (3.04 ± 0.18 Å), as shown in Figure 12A. The CAS study also reported that the substitution of Q498 with alanine was accompanied by a substantial loss in binding free energy (ΔΔ*G*_CoV-2_(Q498A) = −5.36 ± 0.37 kcal/mol).^25^ Consistent with these data, the current *in silico* mutagenesis data and the relative experimental evidence^36^ agree on the interface-disrupting role for all mutants considered, with the interesting exception of the Q498W substitution for which the experiment reports a weak positive effect, while the present MD simulations report a substantial decrease in receptor affinity for this mutant (ΔΔ*G*_CoV-2_(Q498W) = −4.18 ± 0.11 kcal/mol, Figure 20B, Table S2, Figure S29, and Table S31; for a discussion on Q498W data, see Supporting Information).

#### N501, R403, and K417

**Figure 21:**
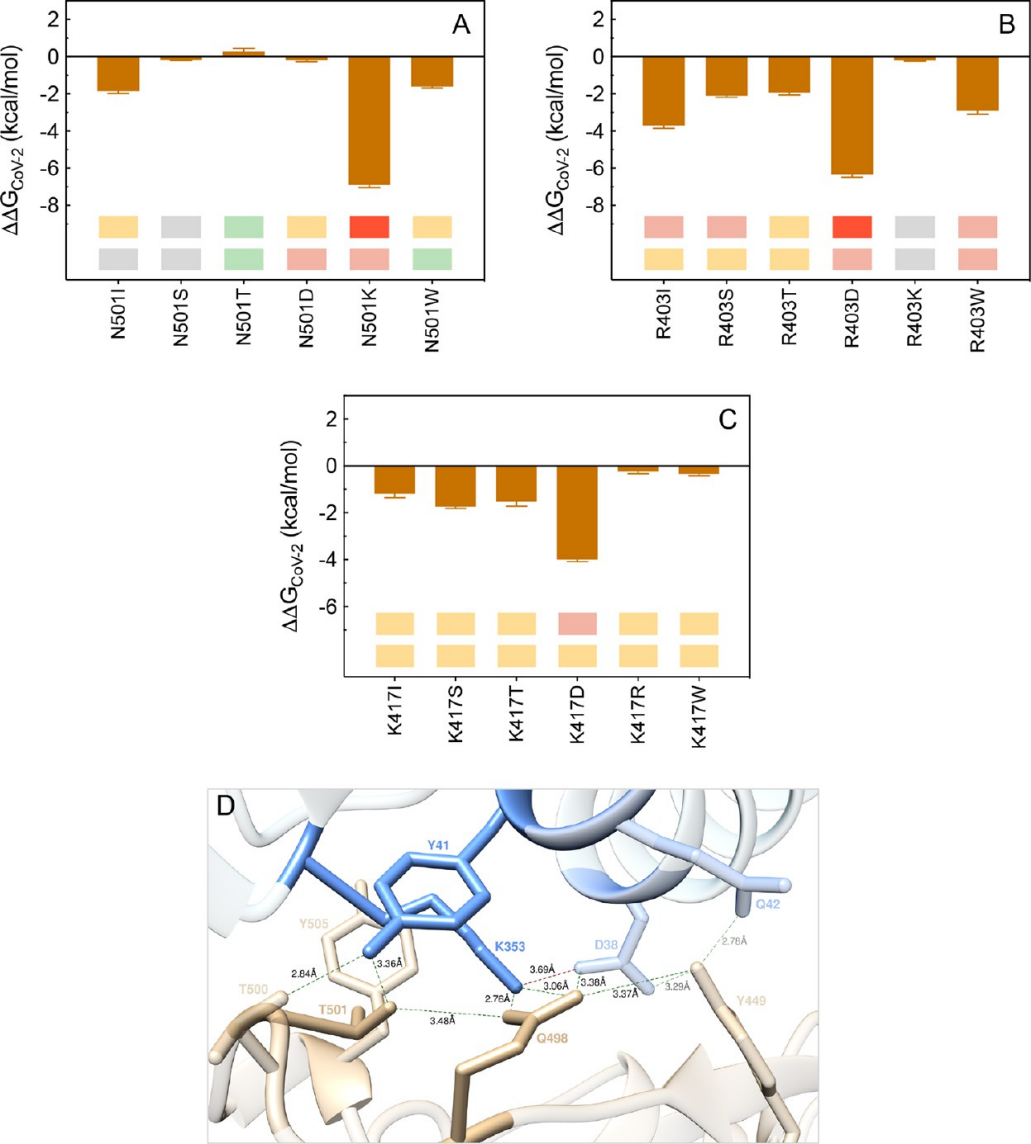
Change in binding free energy predicted by computational mutagenesis of the S-RBD_CoV-2_ residues N501 (A), R403 (B), and K417 (C) for the corresponding ACE2/S-RBD_CoV-2_ complexes. The numerical values of ΔΔ*G*, all related energy terms, and all underlying intermolecular/intramolecular interactions are reported in Table S2, Figures S30–S32, and Tables S32–S34. (D) Main interactions involving the S-RBD_CoV-2_ N501T mutant at the interface with ACE2 as obtained from the relevant equilibrated MD simulations. Images for all other N501, R403, and K417 mutants are shown in Figures S30–S32 (see also Tables S32–S34 for details). Colors and other explanations are as in Figure 2.

Previous modeling^25^ and experimental X-ray/TEM^20−23^ data showed that N501 on S-RBD_CoV-2_ is only involved in one intermolecular HB with ACE2 Y41 (3.23 ± 0.22 Å), a polar interaction with ACE2 K353 across the binding interface, and an internal HB with Q498, as just discussed a few lines earlier (Figure 12A). As such, the total free energy change for mutating N501 in alanine in our CAS study was reported to be small, and equal to ΔΔ*G*_CoV-2_(N501A) = −2.40 ± 0.28 kcal/mol.^25^ Both *in silico* and experimental data^36^ presently suggest mostly neutral-to-disrupting protein/protein binding effects, with an exception made for the T501 mutant, for which computational mutagenesis predicts a weak interface-stabilizing outcome, in agreement with experiment (Figure 21A, Table S2, Figure S30, and Table S32; for a discussion on N501W data, see Supporting Information). As shown in panel D of Figure 21, the T501 mutation is able to establish the same interaction network that characterizes the wild-type N501 S-RBD_CoV-2_/ACE2 complex, with additional stabilization provided by a double (instead of single) intermolecular HB between Q498 on the viral protein and the side chain of K353 on the human receptor (2.76 ± 0.11 Å and 3.06 ± 0.16 Å, respectively) (see also Table S31). Based on these data, the predicted relevant variation of the binding affinity of the T501 variant for ACE2 is equal to ΔΔ*G*_CoV-2_(N501T) = +0.28 ± 0.11 kcal/mol (Figure 21A, Table S2).

R403 is a S-RBD_CoV-2_ residue that salt-bridges ACE2 E37 (3.62 ± 0.39 Å), internally anchors the side chain of D405 in another SB (3.95 ± 0.37 Å), and engages two intramolecular CIs with Y505 (π/c) and Y505 (van der Waals/hydrophobic interactions) (Figure 14A). Mutating this positively charged residue into alanine provided a ΔΔ*G*_CoV-2_(R403A) of −4.25 ± 0.39 kcal/mol,^25^ flagging R403 as another viral protein hot spot for receptor binding. In support of the importance of this residue, mutating R403 into all residues considered both *in silico* and *in vitro*(36) results in detrimental effects on these viral protein/human protein complexes, with a neutral effect for the conservative mutation R403K (Figure 21B, Table S2, Figure S31, and Table S33).

Finally, the viral residue K147 affords one interface stabilizing SB with the side chain of ACE2 D30 (3.85 ± 0.41 Å), along with two intramolecular CIs with F456 (π/c) and L455 (van der Waals/hydrophobic), respectively (Figures 4A and 8A). The abrogation of these interactions resulting from the replacement of this lysine with alanine yielded a moderately unfavorable value of the corresponding binding free energy (ΔΔ*G*_CoV-2_(K417A) = −2.72 ± 0.34 kcal/mol).^25^ The present results, in tandem with the corresponding experimental data,^36^ also support relative interface destabilizing effects in the presence of all mutants considered (Figure 21C, Table S2, Figure S32, and Table S34).

## Conclusions

While preparing this work, the major government entities around the world are acting to address COVID-19 and are issuing emergency use authorization for some of the available COVID-19 vaccines^37^ to face the still-spreading pandemic; the quest for alternative prevention or treatment regimens directed against the SARS-CoV-2 is progressively and dynamically ongoing worldwide. While vaccines directed against this deadly pathogens are becoming available, the repurposing of clinically approved drugs might offer a further, fast lane to anti-COVID-19 effective therapies,^38−41^ whereas alternative approaches to target ACE2 and possibly other host cellular and/or pathogen proteins could ultimately result in active therapeutic and/or prophylactic agents against viral infection.^42−45^ All these alternative strategies might further provide early protection against viral infection by blocking host cell–viral interaction, and therefore reduce the associated severe pathological symptoms; however, extensive *in vitro* and *in vivo* experimental campaigns are unconditionally mandatory before such treatments can be translated into the clinics. From this perspective, the purpose of this work was to provide an atomistic-based, *in silico* view of the role eventually played by possible mutations on both the S-RBD_CoV-2_ and its host cellular receptor ACE2 at the relevant protein/protein interface, with the ultimate goal to suggest precise targets for the structure-based design and development of neutralizing antibodies, vaccines, and protein/protein inhibitors so urgently needed in the current fight against this terrible pandemic. Accordingly, we have simulated different mutation types (hydrophobic, polar, charged, and bulky) at all different residues of ACE2 and S-RBD_CoV-2_ that form most of the protein–protein interface and estimated the variation in the corresponding free energy of binding. Moreover, the computer-based predicted results were challenged against available experimental data.^35,36^ The achievement of a 92% reliability not only validated the adopted *in silico* approach but also allowed a clear-cut molecular rationale for the relevant *in vitro* data. As could be anticipated, most of the studied mutations act as protein–protein interface destabilizers; however, a non-negligible number of mis-sense variations are predicted to enhance ACE2/S-RBD_CoV-2_ binding; in particular, the variants Q24T, T27D/K/W, D30E, H34S/T/K, E35D, Q42K, L79I/W, R357K, and R393K on ACE2 and L455D/W, F456 K/W, Q493K, N501T, and Y505W on the S-protein receptor binding domain, respectively, are expected to increase the affinity of each mutant isoforms for the corresponding protein counterpart. Such mutagenesis methodology and the relevant results could be further adopted/exploited to investigate allelic variants (AVs) of the ACE2 receptor and/or the S-RBD_CoV-2_ progressively discovered in COVID-19 patients, with the ultimate goal of verifying if any of such AVs could be eventually associated with different degrees of clinically observed viral pathogenicity.

## Methods

All calculations reported in this work were performed in AMBER19^46^ starting from the structure of the ACE2/S-RBD_CoV-2_ complex (PDB ID 6M0J)^22^ and optimized in our previous work.^25^ The role of the selected mutations at each protein/protein interface key position was studied by performing a combination of Molecular Mechanics/Poisson–Boltzmann Surface Area (MM/PBSA),^47^ our consolidated computational mutagenesis,^48−56^ and Interaction Entropy methods.^57^ All details are reported in the extended Methods section of the Supporting Information. The coordinate files and force field parameters for the ACE2 Zn^2+^ binding site and the input coordinate file (PDB) were already released as Supporting Information of our previous work.^25^

## Supporting Information Available

Part 1: Tables S1, S3–S20, Figures S1–S18. Further discussion of data for ACE2 mutants D30I, L79S/T, K31W, Q42I/S/T, D355E, and R393T (PDF)
Part 2: Tables S2, S21–S34, Figures S19–S32. Further discussion of data for S-RBD_CoV-2_ mutants L455S, F486W, Q493I/S/T/W, and Q498W (PDF)
Part 3: Extended Methods section (PDF)
