# Supplementary material for: Computational Mutagenesis at the SARS-CoV-2 Spike Protein/Angiotensin-Converting Enzyme 2 Binding Interface: Comparison with Experimental Evidence
Source: ACS Nano. 2021 Mar 18;15(4):6929–48. doi: 10.1021/acsnano.0c10833 (PMC8009103; doi:10.1021/acsnano.0c10833)
Supplement: Supplementary file 1 — nn0c10833_si_001.pdf [file nn0c10833_si_001.pdf]

Supporting information – Part 1 for the paper:

Computational Mutagenesis at the SARS-CoV-2 Spike Protein/Angiotensin-Converting Enzyme 2 Binding Interface: Comparison with Experimental Evidences

Erik Laurini[1,‡], Domenico Marson[1,‡], Suzana Aulic[1], Alice Fermeglia[1], Sabrina Pricl[1,2*]

[1]Molecular Biology and Nanotechnology Laboratory (MolBNL@UniTS), DEA, University of Trieste, 34127 Trieste, Italy
[2]Department of General Biophysics, Faculty of Biology and Environmental Protection, University of Lodz, 90-136 Lodz, Poland

Table S1. Relative binding free energy and its components calculated by computational mutagenesis for the ACE2 residues effectively involved in the binding interface with the S-RBD of SARS-CoV-2 (see the SI Materials and Methods section for details). IE = interaction entropy. $\Delta\Delta G = \Delta G_{WT} - \Delta G_{MUT}$ (see text for details).

	**Q24I**	**Q24S**	**Q24T**	**Q24D**	**Q24K**	**Q24W**		**T27I**	**T27S**	**T27T**	**T27D**	**T27K**	**T27W**
$\Delta\Delta E_{DISP}$	-1.73	-1.81	0.57	-2.03	-2.19	-0.43	$\Delta\Delta E_{vdW}$	0.15	-0.11	-	0.62	0.04	0.47
$\Delta\Delta E_{ELE}$	-0.28	0.09	0.29	-0.92	-1.10	-1.21	$\Delta\Delta E_{ELE}$	-0.38	0.01	-	2.27	1.39	0.51
$\Delta\Delta H$	-2.01	-1.72	0.86	-2.95	-3.29	-1.64	$\Delta\Delta H$	-0.23	-0.10	-	2.89	1.43	0.98
$\Delta\Delta IE$	-0.02	-0.29	0.07	-0.28	-0.39	-0.04	$\Delta\Delta IE$	-0.18	0.02	-	0.22	0.09	0.04
$\Delta\Delta G_{ACE2}$	**-2.03**	**-2.01**	**+0.93**	**-3.23**	**-3.68**	**-1.68**	$\Delta\Delta G_{ACE2}$	**-0.41**	**-0.08**	-	**3.11**	**1.52**	**1.02**
	**(0.14)**	**(0.09)**	**(0.05)**	**(0.17)**	**(0.11)**	**(0.09)**		**(0.13)**	**(0.11)**	-	**(0.09)**	**(0.16)**	**(0.14)**

	**F28I**	**F28S**	**F28T**	**F28D**	**F28K**	**F28W**		**D30I**	**D30S**	**D30T**	**D30E**	**D30K**	**D30W**
$\Delta\Delta E_{DISP}$	-0.79	-1.12	-0.86	-0.99	-0.67	0.15	$\Delta\Delta E_{DISP}$	-0.81	-1.12	-0.84	0.65	-0.21	0.18
$\Delta\Delta E_{ELE}$	-0.02	-0.06	-0.05	-0.65	-1.01	-0.33	$\Delta\Delta E_{ELE}$	-2.22	-1.07	0.15	0.45	-3.40	-0.90
$\Delta\Delta H$	-0.81	-1.18	-0.91	-1.64	-1.68	-0.18	$\Delta\Delta H$	-3.03	-2.19	-0.69	1.10	-3.61	-0.72
$\Delta\Delta IE$	-0.01	-0.09	0.02	0.08	-0.15	-0.01	$\Delta\Delta IE$	-0.78	-0.65	-0.59	0.06	-0.9	-0.55
$\Delta\Delta G_{ACE2}$	**-0.82**	**-1.27**	**-0.89**	**-1.56**	**-1.83**	**-0.19**	$\Delta\Delta G_{ACE2}$	**-3.81**	**-2.84**	**-1.28**	**1.16**	**-4.51**	**-1.27**
	**(0.17)**	**(0.16)**	**(0.14)**	**(0.11)**	**(0.18)**	**(0.13)**		**(0.15)**	**(0.06)**	**(0.10)**	**(0.17)**	**(0.18)**	**(0.11)**

	**K31I**	**K31S**	**K31T**	**K31D**	**K31R**	**K31W**		**H34I**	**H34S**	**H34T**	**H34D**	**H34K**	**H34W**
$\Delta\Delta E_{DISP}$	-2.76	-2.52	-2.48	-2.83	0.22	0.76	$\Delta\Delta E_{DISP}$	-1.24	-0.19	-0.12	-0.30	1.02	-0.32
$\Delta\Delta E_{ELE}$	-2.70	-0.93	-1.02	-1.51	-0.84	-3.78	$\Delta\Delta E_{ELE}$	-0.55	0.79	0.66	-0.62	-0.58	-0.03
$\Delta\Delta H$	-5.46	-3.45	-3.50	-4.34	-0.62	-3.02	$\Delta\Delta H$	-1.79	0.60	0.54	-0.91	0.44	-0.35
$\Delta\Delta IE$	-0.15	-0.18	-0.22	-0.56	0.12	-0.05	$\Delta\Delta IE$	-0.32	0.12	0.11	-0.12	0.09	-0.19
$\Delta\Delta G_{ACE2}$	**-5.61**	**-3.63**	**-3.72**	**-4.90**	**-0.50**	**-3.07**	$\Delta\Delta G_{ACE2}$	**-2.11**	**0.72**	**0.65**	**-1.03**	**0.53**	**-0.54**
	**(0.08)**	**(0.09)**	**(0.13)**	**(0.08)**	**(0.07)**	**(0.18)**		**(0.16)**	**(0.12)**	**(0.07)**	**(0.13)**	**(0.09)**	**(0.12)**

	**E35I**	**E35S**	**E35T**	**E35D**	**E35K**	**E35W**		**E37I**	**E37S**	**E37T**	**E37D**	**E37K**	**E37W**
$\Delta\Delta E_{DISP}$	-1.01	-0.87	-1.07	0.03	0.15	0.79	$\Delta\Delta E_{DISP}$	-1.02	-1.97	-1.61	-0.82	-0.46	0.78
$\Delta\Delta E_{ELE}$	-3.17	-2.86	-2.85	0.83	-4.95	-5.20	$\Delta\Delta E_{ELE}$	-3.06	-2.73	-3.73	-2.99	-5.65	-3.55
$\Delta\Delta H$	-4.18	-3.73	-3.92	0.86	-4.80	-4.41	$\Delta\Delta H$	-4.08	-4.70	-5.34	-3.82	-6.11	-2.77
$\Delta\Delta IE$	-0.47	-0.37	-0.41	0.12	-0.28	-0.45	$\Delta\Delta IE$	-0.99	-0.84	-0.87	-0.78	-0.71	-0.75
$\Delta\Delta G_{ACE2}$	**-4.65**	**-4.10**	**-4.33**	**0.98**	**-5.08**	**-4.86**	$\Delta\Delta G_{ACE2}$	**-5.07**	**-5.54**	**-6.21**	**-4.60**	**-6.82**	**-3.52**
	**(0.11)**	**(0.08)**	**(0.18)**	**(0.11)**	**(0.19)**	**(0.16)**		**(0.17)**	**(0.19)**	**(0.07)**	**(0.19)**	**(0.14)**	**(0.06)**

	**D38I**	**D38S**	**D38T**	**D38E**	**D38K**	**D38W**		**Y41I**	**Y41S**	**Y41T**	**Y41D**	**Y41K**	**Y41W**
$\Delta\Delta E_{DISP}$	-1.78	-0.87	-1.01	-0.20	-1.98	-1.45	$\Delta\Delta E_{DISP}$	-1.10	-1.86	-1.16	-2.19	0.47	-0.78
$\Delta\Delta E_{ELE}$	-2.61	-2.94	-4.05	-0.07	-4.67	-4.40	$\Delta\Delta E_{ELE}$	-2.54	-0.90	-1.64	-2.63	-0.69	-1.32
$\Delta\Delta H$	-4.39	-3.81	-5.06	-0.27	-6.65	-5.85	$\Delta\Delta H$	-3.64	-2.76	-2.80	-4.82	-0.22	-2.10
$\Delta\Delta IE$	-1.09	-0.98	-1.26	-0.08	-1.24	-1.21	$\Delta\Delta IE$	-0.45	-0.48	-0.32	-0.31	0.04	-0.29
$\Delta\Delta G_{ACE2}$	**-5.48**	**-4.79**	**-6.32**	**-0.35**	**-7.89**	**-7.06**	$\Delta\Delta G_{ACE2}$	**-4.09**	**-3.24**	**-3.12**	**-5.13**	**-0.18**	**-2.39**
	**(0.12)**	**(0.13)**	**(0.09)**	**(0.12)**	**(0.19)**	**(0.13)**		**(0.19)**	**(0.12)**	**(0.16)**	**(0.08)**	**(0.11)**	**(0.18)**

	**Q42I**	**Q42S**	**Q42T**	**Q42D**	**Q42K**	**Q42W**		**L79I**	**L79S**	**L79T**	**L79D**	**L79K**	**L79W**
$\Delta\Delta E_{DISP}$	-0.78	-0.19	-0.12	-0.94	0.38	-0.36	$\Delta\Delta E_{DISP}$	0.32	-0.46	-0.28	-0.76	-0.26	0.67
$\Delta\Delta E_{ELE}$	-1.40	0.16	0.19	-1.32	-0.04	-1.01	$\Delta\Delta E_{ELE}$	0.04	0.45	0.36	0.12	0.30	0.33
$\Delta\Delta H$	-2.18	-0.03	0.07	-2.27	0.34	-1.37	$\Delta\Delta H$	0.36	-0.01	0.08	-0.64	0.04	1.00
$\Delta\Delta IE$	-0.28	-0.05	0.03	-0.45	0.11	-0.42	$\Delta\Delta IE$	-0.01	-0.03	0.02	-0.27	-0.22	0.05
$\Delta\Delta G_{ACE2}$	**-2.46**	**-0.08**	**0.10**	**-2.72**	**0.45**	**-1.79**	$\Delta\Delta G_{ACE2}$	**0.35**	**-0.04**	**0.10**	**-0.91**	**-0.18**	**1.05**
	**(0.10)**	**(0.06)**	**(0.11)**	**(0.18)**	**(0.07)**	**(0.19)**		**(0.10)**	**(0.14)**	**(0.16)**	**(0.19)**	**(0.06)**	**(0.15)**

	**M82I**	**M82S**	**M82T**	**M82D**	**M82K**	**M82W**		**Y83I**	**Y83S**	**Y83T**	**Y83D**	**Y83K**	**Y83W**
$\Delta\Delta E_{DISP}$	0.03	-0.59	-0.47	-0.86	0.15	0.26	$\Delta\Delta E_{DISP}$	-1.52	-2.35	-2.39	-1.89	-2.65	-0.37
$\Delta\Delta E_{ELE}$	-0.14	-0.03	-0.23	0.57	-0.12	-0.36	$\Delta\Delta E_{ELE}$	-1.18	-0.51	-0.78	-0.30	-0.67	-0.53
$\Delta\Delta H$	-0.11	-0.62	-0.70	-0.29	0.03	-0.10	$\Delta\Delta H$	-1.70	-2.86	-3.17	-2.19	-3.32	-0.90
$\Delta\Delta IE$	-0.02	-0.05	-0.04	-0.14	0.04	0.01	$\Delta\Delta IE$	-0.28	-0.18	-0.16	-0.18	-0.26	-0.11
$\Delta\Delta G_{ACE2}$	**-0.13**	**-0.67**	**-0.74**	**-0.43**	**0.07**	**-0.09**	$\Delta\Delta G_{ACE2}$	**-2.98**	**-3.04**	**-3.33**	**-2.37**	**-3.58**	**-1.01**
	**(0.05)**	**(0.07)**	**(0.14)**	**(0.16)**	**(0.15)**	**(0.11)**		**(0.19)**	**(0.06)**	**(0.16)**	**(0.17)**	**(0.11)**	**(0.18)**

	K353I	K353S	K353T	K353D	K353R	K353W
$\Delta\Delta E_{DISP}$	-0.72	-2.45	-1.98	-4.31	-1.05	-2.98
$\Delta\Delta E_{ELE}$	-4.75	-3.47	-3.11	-2.07	-0.31	-4.56
$\Delta\Delta H$	-5.47	-5.92	-5.09	-6.38	-1.36	-7.54
$\Delta\Delta IE$	-1.2	-1.02	-0.95	-1.87	-0.07	-1.99
**$\Delta\Delta G_{ACE2}$**	**-6.67 (0.08)**	**-6.94 (0.06)**	**-6.04 (0.09)**	**-8.25 (0.10)**	**-1.43 (0.12)**	**-9.53 (0.15)**
	**R357I**	**R357S**	**R357T**	**R357D**	**R357K**	**R357W**
$\Delta\Delta E_{DISP}$	-1.47	-2.03	-1.52	-1.16	-0.05	-0.74
$\Delta\Delta E_{ELE}$	-1.96	-2.38	-2.68	-2.83	0.95	-1.56
$\Delta\Delta H$	-3.43	-4.41	-4.20	-3.99	0.90	-2.30
$\Delta\Delta IE$	-0.59	-0.67	-0.57	-0.38	0.03	-0.16
**$\Delta\Delta G_{ACE2}$**	**-4.02 (0.15)**	**-5.08 (0.10)**	**-4.77 (0.19)**	**-4.37 (0.05)**	**0.93 (0.11)**	**-2.46 (0.09)**

	D355I	D355S	D355T	D355E	D355K	D355W
$\Delta\Delta E_{DISP}$	-3.29	-2.71	-2.54	0.02	-1.75	-1.01
$\Delta\Delta E_{ELE}$	-4.21	0.05	0.35	-0.06	-3.47	-2.25
$\Delta\Delta H$	-7.50	-2.66	-2.19	-0.04	-5.22	-3.26
$\Delta\Delta IE$	-0.87	-0.56	-0.48	-0.02	-1.19	-0.73
**$\Delta\Delta G_{ACE2}$**	**-8.37 (0.15)**	**-3.22 (0.16)**	**-2.67 (0.10)**	**-0.06 (0.11)**	**-6.41 (0.08)**	**-3.99 (0.07)**
	**R393I**	**R393S**	**R393T**	**R393D**	**R393K**	**R393W**
$\Delta\Delta E_{DISP}$	-0.94	-0.43	-0.36	-0.39	0.30	-0.35
$\Delta\Delta E_{ELE}$	-1.25	-1.45	-1.34	-3.19	0.45	-0.81
$\Delta\Delta H$	-2.19	-1.88	-1.70	-3.58	0.75	-1.16
$\Delta\Delta IE$	-0.39	-0.27	-0.28	-0.47	0.04	-0.41
**$\Delta\Delta G_{ACE2}$**	**-2.58 (0.17)**	**-2.15 (0.19)**	**-1.98 (0.08)**	**-4.05 (0.12)**	**0.79 (0.14)**	**-1.57 (0.09)**

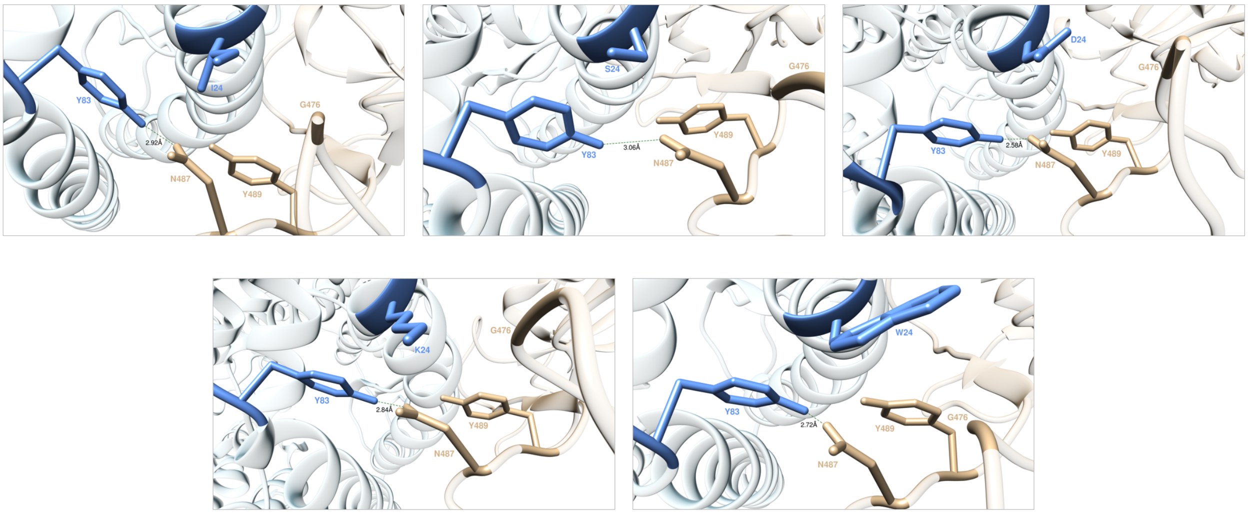

Figure S1. Main interactions involving the ACE2 I24 (top left), S24 (top middle), D24 (top right), K24 (bottom left), and W24 (bottom right) at the interface with S-RBD$_{CoV-2}$ as obtained from the corresponding equilibrated MD simulations. The wild-type Q24 and the T24 mutant are presented and discussed in the main text (Figure 2A-B). In this and all remaining Figures, the secondary structures of ACE2 and S-RBD$_{CoV-2}$ are portrayed as light blue and light sienna ribbons, respectively. Each protein residue under discussion and all other residues directly interacting with them are highlighted in dark matching-colored sticks and labelled; further residues/interactions related to the residue under investigation are evidenced in light matching-colored sticks and labelled in light gray. Hydrogen bonds and salt bridges are represented as dark green and dark red broken lines, respectively, and the relevant average distances are reported (in black) accordingly; new HBs and SBs detected in each mutant complex are also indicated using dark green/red broken lines and black labels. For further details see Tables S1 and S3.

Table S3. Main intermolecular and intramolecular interactions between the wild-type ACE2 residue Q24 and all considered mutants* at the protein-protein interface detected during MD simulations of ACE2 in complex with the RBD of SARS-CoV-2 (COV-2). HB = hydrogen bond; SB = salt bridge; CI = contact interactions, including van der Waals/hydrophobic (vdW/h), polar (p), π/π and π/cation (π/c) interactions. In the HB column, s-s indicates side chain-side chain interactions while s-b (or b-s) and b-b indicate side chain-backbone and backbone-backbone interactions, respectively. Preserved/new or lost interactions are marked with the symbols ✓ and ✗, respectively. Relevant changes in the type/nature of the interactions are indicated in parenthesis. For HBs and SBs, the relevant average lengths (in Å) are also reported (their standard deviations, all within 10%, are not shown for clarity). Charges not involved in SBs at the protein/protein interface are also indicated. *Mutant T24 is discussed in detail in main text.

**HB**	**COV-2**	**ACE2**	**Q24**	**I24**	**S24**	**T24**	**D24**	**K24**	**W24**
s-s	N487	X24	✓(3.03)	✗	✗	✓(3.13)	✗	✗(p)	✗
s-s	N487	Y83	✓(2.88)	✓(2.92)	✓(3.06)	✓(2.73)	✓(2.58)	✓(2.84)	✓(2.72)
**HB**	**ACE2**	**ACE2**	**Q24**	**I24**	**S24**	**T24**	**D24**	**K24**	**W24**
s-s	Y83	X24	✗	✗	✗	✓(3.45)	✗	✗	✗
**CI**	**COV-2**	**ACE2**	**Q24**	**I24**	**S24**	**T24**	**D24**	**K24**	**W24**
vdW/h	G476	X24	✓	✗	✗	✓	✓	✗	✗
vdW/h	Y489	X24	✓	✓	✓(p)	✓(p)	✗	✗	✓
vdW/h	Y83	X24	✓	✗	✗	✓	✗	✓(p)	✗

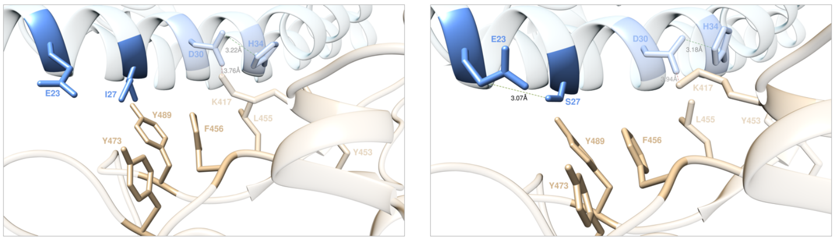

Figure S2. Main interactions involving the ACE2 I27 (left) and S27 (right) at the interface with S-RBD$_{CoV-2}$ as obtained from the corresponding equilibrated MD simulations. The wild-type residue T27 and the D27, K27, and W27 mutants are presented and discussed in the main text (Figure 2C-F). Colors and other explanations as in Figure S1. For further details see Tables S1 and S4.

Table S4. Main intermolecular and intramolecular interactions between the wild-type ACE2 residue T27 and all considered mutants* at the protein-protein interface detected during MD simulations of ACE2 in complex with the RBD of SARS-CoV-2 (COV-2). Acronyms and other explanations as in Table S3. *Mutants D27, K27 and W27 are discussed in detail in main text.

**HB**	**COV-2**	**ACE2**	**T27**	**I27**	**S27**	**D27**	**K27**	**W27**
s-s	Y473	X27	✗	✗	✗	✓(2.68)	✗	✗
s-s	Y453	H34	✗(p)	✗(p)	✗(p)	✓(3.37)	✓(3.24)	✓(3.48)
**HB**	**ACE2**	**ACE2**	**T27**	**I27**	**S27**	**D27**	**K27**	**W27**
b-s	E23	X27	✓(3.05)	✗	✓(3.07)	✗	✓(SB,3.74)	✗
s-s	D30	H34	✓(3.31)	✓(3.22)	✓(3.18)	✓(2.90)	✓(3.04)	✓(3.37)
**SB**	**COV-2**	**ACE2**	**T27**	**I27**	**S27**	**D27**	**K27**	**W27**
	K417	X27	✗	✗	✗	✓(3.87)	✗	✗
	K417	D30	✓(3.85)	✓(3.76)	✓(3.94)	✓(3.67)	✓(3.84)	✓(3.91)
**CI**	**COV-2**	**ACE2**	**T27**	**I27**	**S27**	**D27**	**K27**	**W27**
vdW/h	F456	X27	✓	✓	✓	✓	✓	✓(π/π)
vdW/h	Y489	X27	✓	✓	✓	✓	✓	✓
vdW/h	Y473	X27	✓	✓	✓	✓	✓	✓

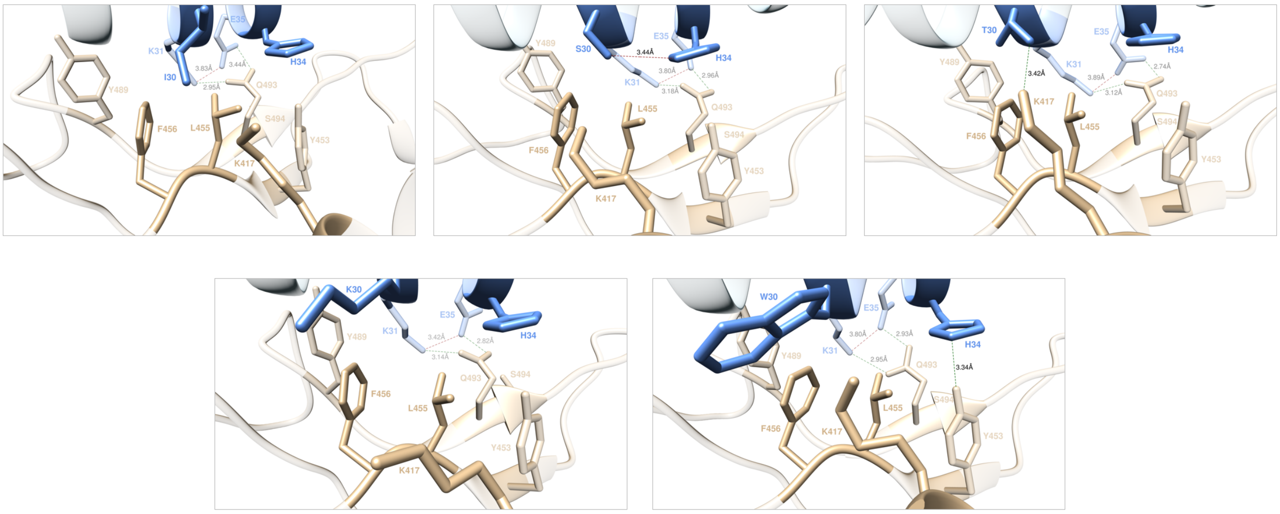

Figure S3. Main interactions involving the ACE2 I30 (top left), S30 (top middle), T30 (top right), K30 (bottom left) and W30 (bottom right) at the interface with S-RBD_{CoV-2} as obtained from the corresponding equilibrated MD simulations. The wild-type D30 and the E30 mutant are presented and discussed in the main text (Figure 4A-B). Colors and other explanations as in Figure S1. For further details see Tables S1 and S5.

Table S5. Main intermolecular and intramolecular interactions between the wild-type ACE2 residue D30 and all considered mutants* at the protein-protein interface detected during MD simulations of ACE2 in complex with the RBD of SARS-CoV-2 (COV-2). Acronyms and other explanations as in Table S3. *Mutant E30 is discussed in detail in main text.

**HB**	**COV-2**	**ACE2**	**D30**	**I30**	**S30**	**T30**	**E30**	**K30**	**W30**
s-s	Q493	K31	✓(3.04)	✓(2.95)	✓(3.18)	✓(3.12)	✓(2.85)	✓(3.14)	✓(2.95)
s-s	Q493	E35	✓(2.94)	✓(3.44)	✓(2.96)	✓(2.74)	✓(3.31)	✓(2.82)	✓(2.93)
**HB**	**ACE2**	**ACE2**	**D30**	**I30**	**S30**	**T30**	**E30**	**K30**	**W30**
s-s	H34	X30	✓(3.31)	✗	✓(3.44)	✗	✓(2.96)	✗	✗
**HB**	**COV2**	**COV2**	**D30**	**I30**	**S30**	**T30**	**E30**	**K30**	**W30**
s-s	Q493	S494	✓(3.24)	✗	✗	✗	✗	✗	✗
**SB**	**COV-2**	**ACE2**	**D30**	**I30**	**S30**	**T30**	**E30**	**K30**	**W30**
	K417	X30	✓(3.85)	✗	✗	✓(HB,3.42)	✓(3.01)	✗	✗
**SB**	**ACE2**	**ACE2**	**D30**	**I30**	**S30**	**T30**	**E30**	**K30**	**W30**
	K31	E35	✓(3.94)	✓(3.83)	✓(3.80)	✓(3.89)	✓(3.08)	✓(3.42)	✓(3.80)
**CI**	**COV-2**	**ACE2**	**D30**	**I30**	**S30**	**T30**	**E30**	**K30**	**W30**
vdW/h	F456	X30	✓	✓	✓	✓	✓	✓	✗
vdW/h	L455	X30	✓	✓	✓	✓	✓	✗	✗
p	Y453	H34	✓	✓	✓	✓	✓	✓	✓(HB,3.34)
vdw/h	Y489	K31	✓	✓	✓	✓	✓	✓	✓

The qualitative *in silico*/experimental data comparison shown in Figure 5A (main text) for the mutagenesis of ACE D30 into the 6 residues considered it appears that the only different trend concerns mutation I30, for which a slightly beneficial effect is reported by experiment while an interface disrupting effect is predicted ($\Delta\Delta G_{ACE2}$(D30I) = -3.81 ± 0.15 kcal/mol, Table S1). The analysis of the corresponding MD trajectory for the ACE2 I30 mutant in complex with the S-RBD_{CoV-2} (Figure S3 (top left) and Table S5) reveals that, with respect to the wild-type assembly, three important interface interactions are no longer detected in the presence of the hydrophobic mutant residue: i) the intramolecular HB between the side chains of D30 and H34 on ACE2; ii) the intramolecular Q493-S494 HB on S-RBD_{CoV-2}, and iii) the intermolecular, charge-neutralizing SB between D30 on ACE2 and K417 on the viral protein. According to Shang *et al.*[1],

neutralizing the charges of the lysines is a key factor in the binding of coronavirus RBDs to ACE2. Moreover, as reported by Lan *et al*.[2] and verified in our previous work,[3] K417 is the only residue on the S-RBD_{CoV-2} able to form an intermolecular SB with ACE D30 (Figure 4A, main text). Interestingly, in the receptor binding site of SARS-CoV K417 is replaced by V404, and this residue fails to participate in ACE2 binding.[2] Finally, in the experimental ACE deep mutagenesis study reported by Chan *et al*.[4] similar substitutions at ACE position 30 – *i.e.*, D30M, D30L and D30A – are also reported to be interface disrupting mutations. Accordingly, we retain that a charged-to-hydrophobic substitution at this receptor position ultimately exerts destabilizing effects on the ACE2/S-RBD_{CoV-2} complex formation.

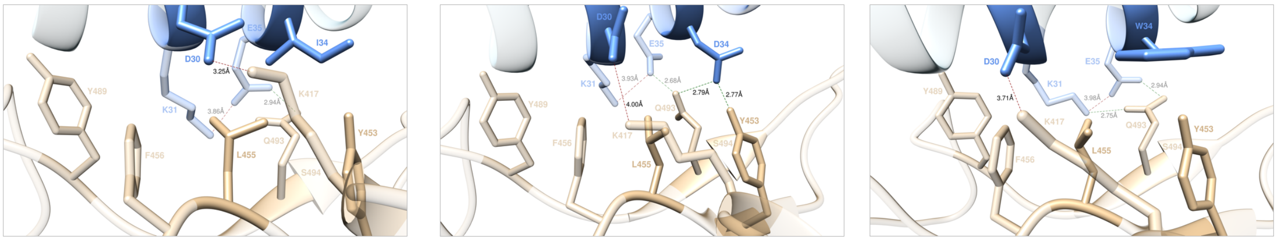

Figure S4. Main interactions involving the ACE2 I34 (left), D34 (middle), and W34 (right) at the interface with S-RBD$_{CoV\text{-}2}$ as obtained from the corresponding equilibrated MD simulations. The wild-type H34 and the S34, T34, and K34 mutants are presented and discussed in the main text (Figure 4A,C-E). Colors and other explanations as in Figure S1. For further details see Tables S1 and S6.

Table S6. Main intermolecular and intramolecular interactions between the wild-type ACE2 residue H34 and all considered mutants* at the protein-protein interface detected during MD simulations of ACE2 in complex with the RBD of SARS-CoV-2 (COV-2). Acronyms and other explanations as in Table S3. *Mutants S34, T34, and K34 are discussed in detail in main text.

**HB**	**COV-2**	**ACE2**	**H34**	**I34**	**S34**	**T34**	**D34**	**K34**	**W34**
s-s	Q493	K31	✓(3.04)	✗	✓(3.39)	✓(2.86)	✗	✓(3.14)	✓(2.75)
s-s	Q493	E35	✓(2.94)	✓(2.94)	✓(3.35)	✓(2.69)	✓(2.68)	✓(2.80)	✓(2.94)
s-s	Q493	X34	✗	✗	✓(2.85)	✓(3.45)	✓(2.79)	✗	✗
**HB**	**ACE2**	**ACE2**	**H34**	**I34**	**S34**	**T34**	**D34**	**K34**	**W34**
s-s	D30	X34	✓(3.31)	✗	✗	✗	✗	✗	✗
**HB**	**COV2**	**COV2**	**H34**	**I34**	**S34**	**T34**	**D34**	**K34**	**W34**
s-s	Q493	S494	✓(3.24)	✗	✗	✗	✗	✗	✗
**SB**	**COV-2**	**ACE2**	**H34**	**I34**	**S34**	**T34**	**D34**	**K34**	**W34**
	K417	D30	✓(3.85)	✓(3.25)	✓(3.47)	✓(3.69)	✓(4.00)	✓(3.82,3.88)	✓(3.71)
**SB**	**ACE2**	**ACE2**	**H34**	**I34**	**S34**	**T34**	**D34**	**K34**	**W34**
	K31	E35	✓(3.94)	✓(3.86)	✓(3.06)	✓(3.62)	✓(3.93)	✓(3.81)	✓(3.98)
**CI**	**COV-2**	**ACE2**	**H34**	**I34**	**S34**	**T34**	**D34**	**K34**	**W34**
vdW/h	F456	D30	✓	✗	✓	✓	✓	✓	✓
vdW/h	L455	D30	✓	✓	✓	✓	✓	✓	✓
p	Y453	X34	✓	✗	✗	✗	✓(HB,2.77)	✓	✓
vdw/h	Y489	K31	✓	✓	✓	✓	✓	✓	✓

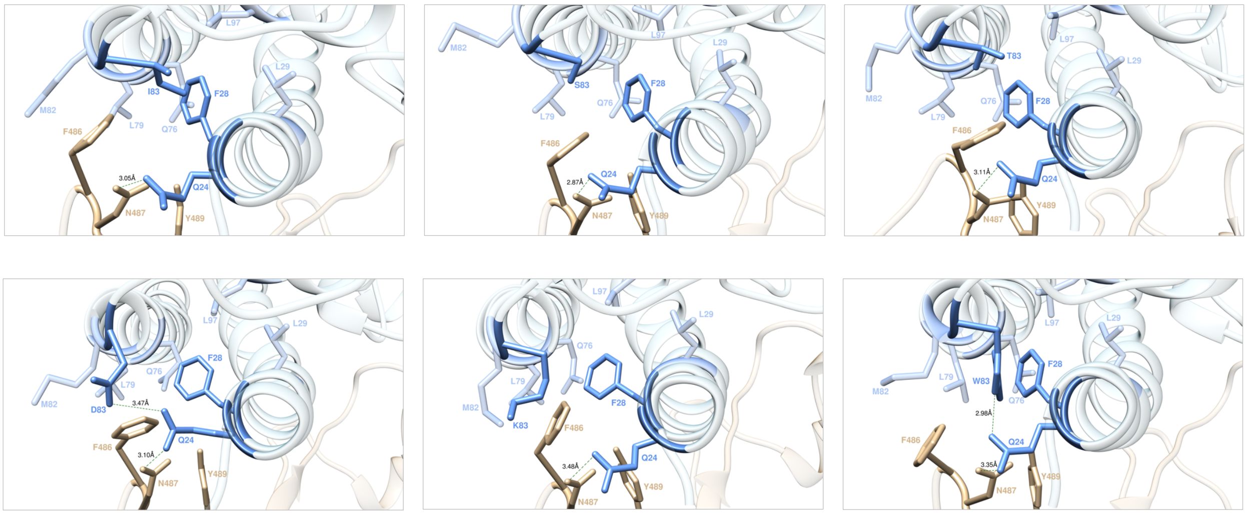

Figure S5. Main interactions involving the ACE2 I83 (top left), S83 (top middle), T83 (top right), D83 (bottom left), K83 (bottom middle) and W83 (bottom right) at the interface with S-RBD$_{CoV\text{-}2}$ as obtained from the corresponding equilibrated MD simulations. The wild-type Y83 is presented and discussed in the main text (Figure 6A). Colors and other explanations as in Figure S1. For further details see Tables S1 and S7.

Table S7. Main intermolecular and intramolecular interactions between the wild-type ACE2 residue Y83 and all considered mutants at the protein-protein interface detected during MD simulations of ACE2 in complex with the RBD of SARS-CoV-2 (COV-2). Acronyms and other explanations as in Table S3.

**HB**	**COV-2**	**ACE2**	**Y83**	**I83**	**S83**	**T83**	**D83**	**K83**	**W83**
s-s	N487	X83	✓(2.88)	✗	✗	✗	✗	✗	✗
s-s	N487	Q24	✓(3.03)	✓(3.05)	✓(2.87)	✓(3.11)	✓(3.10)	✓(3.48)	✓(3.35)
**HB**	**ACE2**	**ACE2**	**Y83**	**I83**	**S83**	**T83**	**D83**	**K83**	**W83**
s-s	Q24	X83	✗	✗	✗	✗	✓(3.47)	✗	✓(2.98)
**CI**	**COV-2**	**ACE2**	**Y83**	**I83**	**S83**	**T83**	**D83**	**K83**	**W83**
vdW/h	F486	X83	✓	✗	✗	✗	✓	✓	✗
p	Y489	X83	✓	✗	✗	✗	✗	✗	✗
**CI**	**ACE2**	**ACE2**	**Y83**	**I83**	**S83**	**T83**	**D83**	**K83**	**W83**
vdW/h	F28	X83	✓	✓	✓	✓	✗	✓	✓

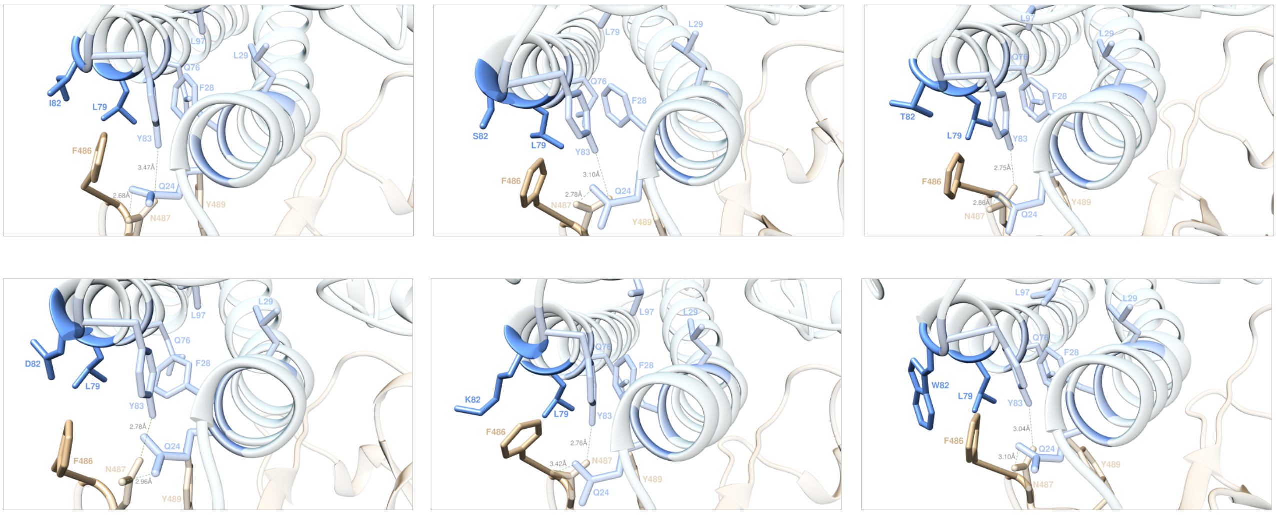

Figure S6. Main interactions involving the ACE2 I82 (top left), S82 (top middle), T82 (top right), D82 (bottom left), K82 (bottom middle) and W82 (bottom right) at the interface with S-RBD$_{CoV-2}$ as obtained from the corresponding equilibrated MD simulations. The wild-type M82 is presented and discussed in the main text (Figure 6A). Colors and other explanations as in Figure S1. For further details see Tables S1 and S8.

Table S8. Main intermolecular and intramolecular interactions between the wild-type ACE2 residue M82 and all considered mutants at the protein-protein interface detected during MD simulations of ACE2 in complex with the RBD of SARS-CoV-2 (COV-2). Acronyms and other explanations as in Table S3.

**HB**	**COV-2**	**ACE2**	**M82**	**I82**	**S82**	**T82**	**D82**	**K82**	**W82**
s-s	N487	Y83	✓(2.88)	✓(3.47)	✓(3.10)	✓(2.75)	✓(2.78)	✓(2.76)	✓(3.04)
s-s	N487	Q24	✓(3.03)	✓(2.68)	✓(2.78)	✓(2.86)	✓(2.96)	✓(3.42)	✓(3.10)
**CI**	**COV-2**	**ACE2**	**M82**	**I82**	**S82**	**T82**	**D82**	**K82**	**W82**
vdW/h	F486	X82	✓	✓	✗	✗	✓	✓	✓
vdw/h	F486	L79	✓	✓	✓	✓	✗	✓	✓
vdW/h	F486	Y83	✓	✓	✓	✗	✗	✓	✓
p	Y489	Y83	✓	✓	✓	✓	✓	✓	✓
**CI**	**ACE2**	**ACE2**	**M82**	**I82**	**S82**	**T82**	**D82**	**K82**	**W82**
vdW/h	L79	X82	✓	✓	✗	✗	✓	✓	✓
vdW/h	F28	L79	✓	✓	✗	✗	✓	✓	✓

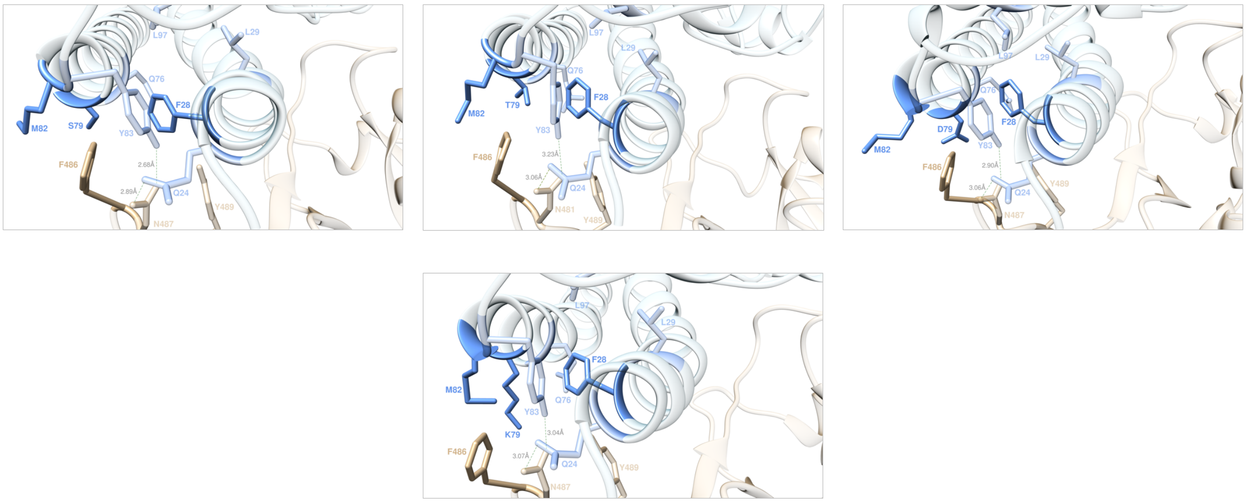

Figure S7. Main interactions involving the ACE2 S79 (top left), T79 (top middle), D79 (top right), and K79 (bottom), at the interface with S-RBD$_{CoV-2}$ as obtained from the corresponding equilibrated MD simulations. The wild-type L79 and the mutants I79 and W79 are presented and discussed in the main text (Figure 6A-C). Colors and other explanations as in Figure S1. For further details see Tables S1 and S9.

Table S9. Main intermolecular and intramolecular interactions between the wild-type ACE2 residue L79 and all considered mutants* at the protein-protein interface detected during MD simulations of ACE2 in complex with the RBD of SARS-CoV-2 (COV-2). Acronyms and other explanations as in Table S3. *Mutants I79 and W79 are discussed in detail in main text.

**HB**	**COV-2**	**ACE2**	**L79**	**I79**	**S79**	**T79**	**D79**	**K79**	**W79**
s-s	N487	Y83	✓(2.88)	✓(2.80)	✓(2.68)	✓(3.23)	✓(2.90)	✓(3.04)	✓(2.69)
s-s	N487	Q24	✓(3.03)	✓(2.99)	✓(2.89)	✓(3.06)	✓(3.06)	✓(3.07)	✓(3.01)
**CI**	**COV-2**	**ACE2**	**L79**	**I79**	**S79**	**T79**	**D79**	**K79**	**W79**
vdw/h	F486	X79	✓	✓	✓	✓	✓	✓	✓(π/π)
vdW/h	F486	M82	✓	✓	✓	✓	✗	✓	✓
p	Y489	Y83	✓	✓	✓	✓	✓	✓	✓
**CI**	**ACE2**	**ACE2**	**L79**	**I79**	**S79**	**T79**	**D79**	**K79**	**W79**
vdW/h	M82	X79	✓	✓	✓	✓	✗	✓	✓
vdW/h	F28	X79	✓	✓	✓	✓	✗	✓	✓
vdW/h	F28	Y83	✓	✓	✓	✓	✓	✓	✓

As it can be inferred by comparing the top left and middle panels in Figure S7 and the data listed in Table S9, according to our MD simulations the two ACE2 mutants S79 and T79 both establish the same interaction network seen from the wild-type protein (Figure 6A in main text). The small difference resides only in the somewhat longer length of the two intermolecular HBs involving Y83 and Q24 on ACE2 and N487 on the S-protein in the case of the L79T mutant complex. In agreement with this, the relevant $\Delta\Delta G$ values are therefore very small and comparable ($\Delta\Delta G_{ACE2}$(L79S)= -0.04 ± 0.14 kcal/mol and $\Delta\Delta G_{ACE2}$(L79T)= -0.10 ± 0.16 kcal/mol, respectively, Figure 7C and Table S1), supporting a neutral effect for both these ACE2 substitutions on the affinity for the viral protein.

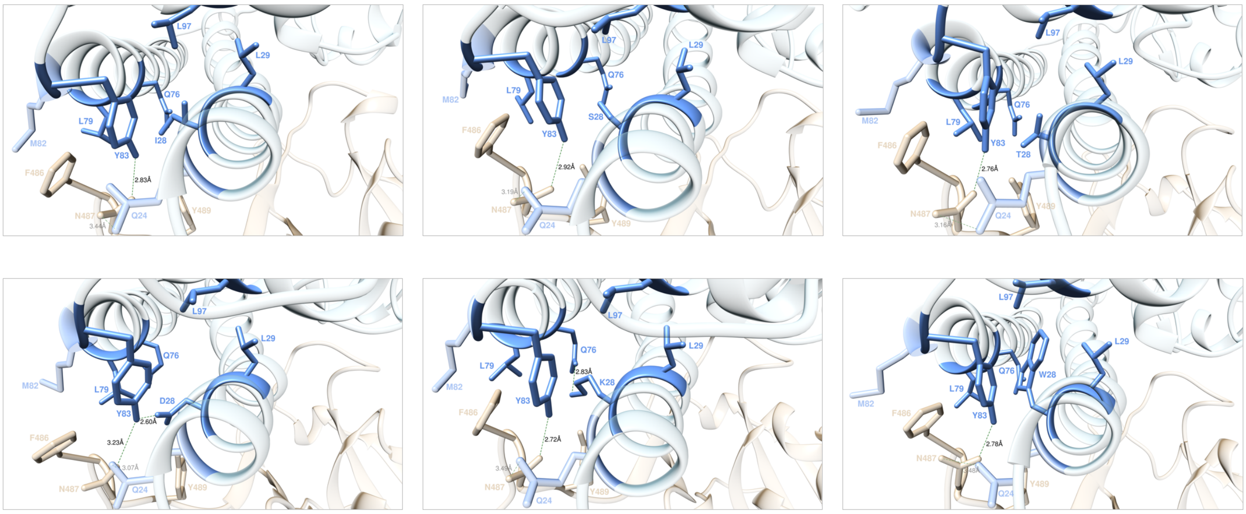

Figure S8. Main interactions involving the ACE2 I28 (top left), S28 (top middle), T28 (top right), D28 (bottom left), K28 (bottom middle) and W28 (bottom right) at the interface with S-RBD$_{CoV-2}$ as obtained from the corresponding equilibrated MD simulations. The wild-type F82 is presented and discussed in the main text (Figure 6A). Colors and other explanations as in Figure S1. For further details see Tables S1 and S10.

Table S10. Main intermolecular and intramolecular interactions between the wild-type ACE2 residue F28 and all considered mutants at the protein-protein interface detected during MD simulations of ACE2 in complex with the RBD of SARS-CoV-2 (COV-2). Acronyms and other explanations as in Table S3.

**HB**	**COV-2**	**ACE2**	**F28**	**I28**	**S28**	**T28**	**D28**	**K28**	**W28**
s-s	N487	Y83	✓(2.88)	✓(2.83)	✓(2.92)	✓(2.76)	✓(3.23)	✓(2.72)	✓(2.78)
s-s	N487	Q24	✓(3.03)	✓(3.44)	✓(3.19)	✓(3.16)	✓(3.07)	✓(3.49)	✓(3.48)
**CI**	**COV-2**	**ACE2**	**F28**	**I28**	**S28**	**T28**	**D28**	**K28**	**W28**
vdw/h	F486	L79	✓	✗	✗	✓	✗	✓	✓
p	Y489	Y83	✓	✓	✓	✓	✗	✗	✗
**CI**	**ACE2**	**ACE2**	**F28**	**I28**	**S28**	**T28**	**D28**	**K28**	**W28**
vdW/h	L29	X28	✓	✗	✗	✗	✗	✗	✓
vdW/h	Q76	X28	✓	✓	✓	✓	✗	✓(HB,2.83)	✓
vdW/h	Y83	X28	✓	✓	✓	✓	✓(HB,2.60)	✗	✓
vdW/h	L79	X28	✓	✗	✗	✗	✓	✗	✗
vdW/h	L97	X28	✓	✗	✗	✗	✗	✗	✓

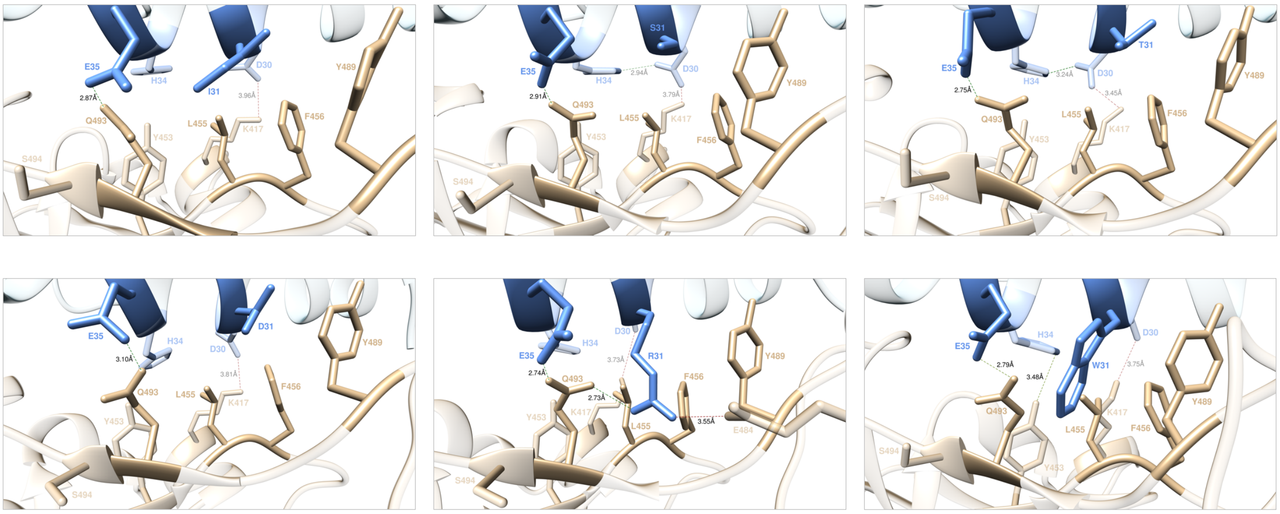

Figure S9. Main interactions involving the ACE2 I31 (top left), S31 (top middle), T31 (top right), D31 (bottom left), R31 (bottom middle) and W31 (bottom right) at the interface with S-RBD$_{CoV-2}$ as obtained from the corresponding equilibrated MD simulations. The wild-type K31 is presented and discussed in the main text (Figure 8A). Colors and other explanations as in Figure S1. For further details see Tables S1 and S11.

Table S11. Main intermolecular and intramolecular interactions between the wild-type ACE2 residue K31 and all considered mutants at the protein-protein interface detected during MD simulations of ACE2 in complex with the RBD of SARS-CoV-2 (COV-2). Acronyms and other explanations as in Table S3.

**HB**	**COV-2**	**ACE2**	**K31**	**I31**	**S31**	**T31**	**D31**	**R31**	**W31**
s-s	Q493	X31	✓(3.04)	✗	✗	✗	✗	✓(2.73)	✗
s-s	Q493	E35	✓(2.94)	✓(2.87)	✓(2.91)	✓(2.75)	✓(3.10)	✓(2.74)	✓(2.79)
**SB**	**ACE2**	**ACE2**	**K31**	**I31**	**S31**	**T31**	**D31**	**R31**	**W31**
	E35	X31	✓(3.94)	✗	✗	✗	✗	✗	✗
**HB**	**COV-2**	**COV-2**	**K31**	**I31**	**S31**	**T31**	**D31**	**R31**	**W31**
s-s	Q493	S494	✓(3.24)	✗	✗	✗	✗	✗	✗
**SB**	**COV-2**	**ACE2**	**K31**	**I31**	**S31**	**T31**	**D31**	**R31**	**W31**
	E484	X31	✗	✗	✗	✗	✗	✓(3.55)	✗
	K417	D30	✓(3.85)	✓(3.96)	✓(3.79)	✓(3.45)	✓(3.81)	✓(3.73)	✓(3.75)
**HB**	**ACE2**	**ACE2**	**K31**	**I31**	**S31**	**T31**	**D31**	**R31**	**W31**
s-s	H34	D30	✓(3.31)	✗	✓(2.94)	✓(3.24)	✗	✗	✗
**CI**	**COV-2**	**ACE2**	**K31**	**I31**	**S31**	**T31**	**D31**	**R31**	**W31**
vdw/h	Y489	X31	✓	✓	✗	✓	✓	✓	✓
vdW/h	L455	X31	✓	✓	✓	✓	✓	✓	✓
vdW/h	F456	X31	✓	✓	✓	✓	✓	✓	✓
vdW/h	F456	D30	✓	✓	✓	✓	✓	✗	✓
vdW/h	L455	D30	✓	✗	✓	✗	✗	✓	✗
p	Y453	H34	✓	✓	✓	✓	✓	✓	✓(HB,3.48)

According to the experimental study by Chan *et al.*,[4] the substitution of the long, positively-charged K31 residue with the bulky and hydrophobic tryptophan results in a positive (*i.e.*, stabilizing) effect the corresponding protein/protein interface. On the other hand, our simulations predict that, when W replaces the native K at the same position, the affinity of the mutant receptor for the viral S-protein decreases ($\Delta\Delta G_{ACE2}$(K31W) = -3.07 ± 0.18 kcal/mol, Figure 9A and

Table S1). The main reasons for this loss in binding free energy can be rationalized on the basis of the following major missing interactions at the relative W31 mutant ACE2/S-RBD_{CoV-2} interface: i) the charge-neutralizing ACE2 intramolecular SB between K31 and E35, ii) the intermolecular HB between K31 and the viral residue Q493; iii) the S-protein RBD internal HB between Q493 and S494, and iv) the structurally important intramolecular ACE2 HB between D30 and H34 (Table S11). These MD-based evidences along with the importance of lysine charge switch-off in the formation of the ACE2/S-RBD_{CoV-2} complex[1] in our opinion support the negative effect of the K31W ACE2 mutation at the human receptor/viral protein interface.

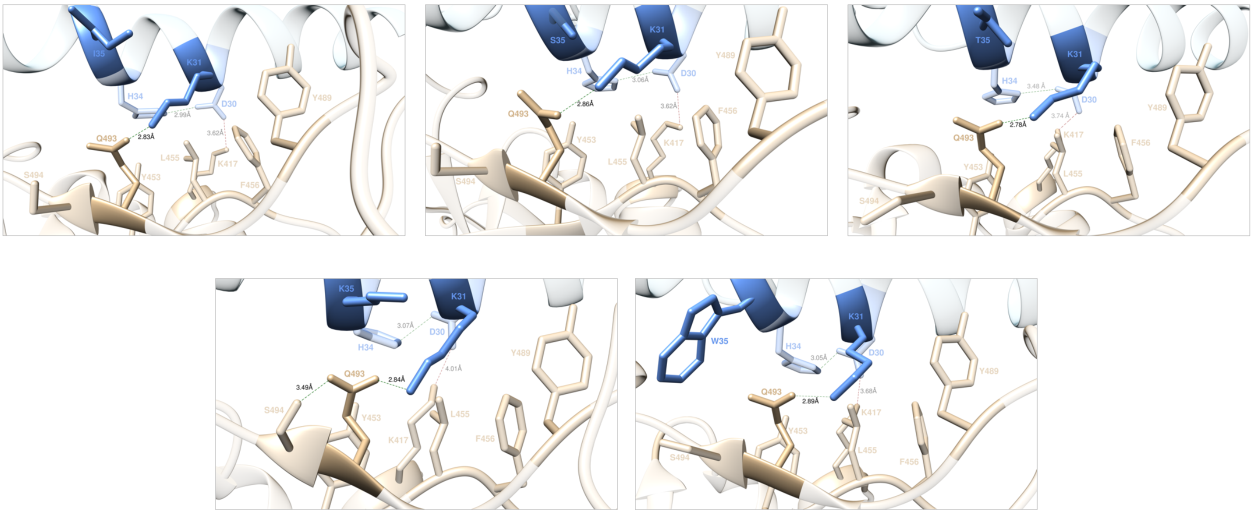

Figure S10. Main interactions involving the ACE2 I35 (top left), S35 (top middle), T35 (top right), K35 (bottom left) and W35 (bottom right) at the interface with S-RBD$_{CoV-2}$ as obtained from the corresponding equilibrated MD simulations. The wild-type E35 and the D35 mutants are presented and discussed in the main text (Figure 8A-B). Colors and other explanations as in Figure S1. For further details see Tables S1 and S12.

Table S12. Main intermolecular and intramolecular interactions between the wild-type ACE2 residue E35 and all considered mutants* at the protein-protein interface detected during MD simulations of ACE2 in complex with the RBD of SARS-CoV-2 (COV-2). Acronyms and other explanations as in Table S3. *Mutant D35 is discussed in detail in main text.

**HB**	**COV-2**	**ACE2**	**E35**	**I35**	**S35**	**T35**	**D35**	**K35**	**W35**
s-s	Q493	X35	✓(2.94)	✗	✗	✗	✓(2.83)	✗	✗
s-s	Q493	K31	✓(3.04)	✓(2.83)	✓(2.86)	✓(2.78)	✓(3.11)	✓(2.84)	✓(2.89)
**SB**	**ACE2**	**ACE2**	**E35**	**I35**	**S35**	**T35**	**D35**	**K35**	**W35**
	K31	X35	✓(3.94)	✗	✗	✗	✓(3.00,3.19)	✗	✗
**HB**	**COV-2**	**COV-2**	**E35**	**I35**	**S35**	**T35**	**D35**	**K35**	**W35**
s-s	Q493	S494	✓(3.24)	✗	✗	✗	✗	✓(3.49)	✗
**SB**	**COV-2**	**ACE2**	**E35**	**I35**	**S35**	**T35**	**D35**	**K35**	**W35**
	K417	D30	✓(3.85)	✓(3.62)	✓(3.62)	✓(3.74)	✓(3.75)	✓(4.01)	✓(3.68)
**HB**	**ACE2**	**ACE2**	**E35**	**I35**	**S35**	**T35**	**D35**	**K35**	**W35**
s-s	H34	D30	✓(3.31)	✓(2.99)	✓(3.06)	✓(3.48)	✓(2.91)	✓(3.07)	✓(3.05)
**CI**	**COV-2**	**ACE2**	**E35**	**I35**	**S35**	**T35**	**D35**	**K35**	**W35**
vdw/h	Y489	K31	✓	✗	✗	✗	✓	✗	✓
vdw/h	L455	K31	✓	✓	✓	✓	✓	✓	✓
vdw/h	F456	K31	✓	✗	✗	✓	✓	✗	✗
vdW/h	F456	D30	✓	✗	✓	✗	✓	✗	✗
vdW/h	L455	D30	✓	✗	✗	✗	✓	✗	✗
p	Y453	H34	✓	✓	✓	✓	✓	✓	✓

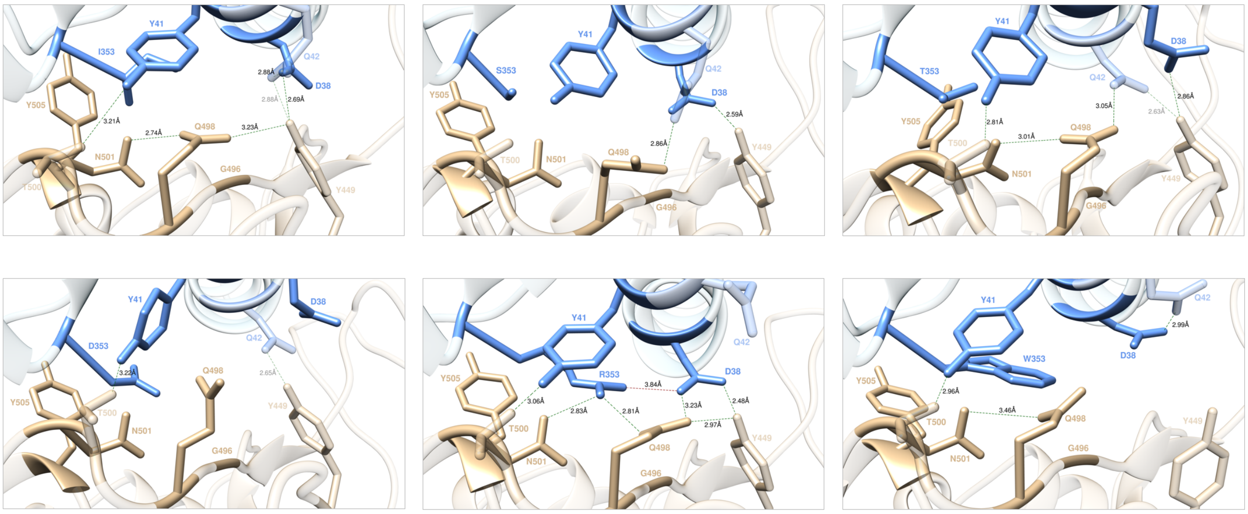

Figure S11. Main interactions involving the ACE2 I353 (top left), S353 (top middle), T353 (top right), D353 (bottom left), R353 (bottom middle) and W353 (bottom right) at the interface with S-RBD$_{CoV-2}$ as obtained from the corresponding equilibrated MD simulations. The wild-type K353 is presented and discussed in the main text (Figure 10A). Colors and other explanations as in Figure S1. For further details see Tables S1 and S13.

Table S13. Main intermolecular and intramolecular interactions between the wild-type ACE2 residue K353 and all considered mutants at the protein-protein interface detected during MD simulations of ACE2 in complex with the RBD of SARS-CoV-2 (COV-2). Acronyms and other explanations as in Table S3.

**HB**	**COV-2**	**ACE2**	**K353**	**I353**	**S353**	**T353**	**D353**	**R353**	**W353**
s-s	Q498	X353	✓(2.87)	✗	✗	✗	✗	✓(2.81)	✗
b-s	G496	X353	✓(2.95)	✗	✗	✗	✗	✗	✗
s-s	T500	Y41	✓(3.08)	✓(3.21)	✗(p)	✓(2.81)	✓(3.22)	✓(3.06)	✓(2.96)
s-s	N501	Y41	✓(3.23)	✗	✗(p)	✗(p)	✗	✗(p)	✗(p)
s-s	Q498	D38	✓(2.92)	✗	✗	✗	✗	✓(3.23)	✗
s-s	N501	X353	✗(p)	✗(p)	✗(p)	✗	✗(p)	✓(2.83)	✗(p)
s-s	Y449	D38	✓(2.92)	✓(2.69)	✓(2.59)	✓(2.86)	✗	✓(2.48)	✗
s-s	Y449	Q42	✓(3.03)	✓(2.88)	✗	✓(2.63)	✓(2.65)	✗	✗
s-s	Q498	Q42	✗	✗	✓(2.86)	✓(3.05)	✗	✗	✗
**SB**	**ACE2**	**ACE2**	**K353**	**I353**	**S353**	**T353**	**D353**	**R353**	**W353**
	D38	X353	✓(3.66)	✗	✗	✗	✗	✓(3.84)	✗
**HB**	**ACE2**	**ACE2**	**K353**	**I353**	**S353**	**T353**	**D353**	**R353**	**W353**
b-s	D38	Q42	✓(3.04)	✓(s-s,2.88)	✗	✗	✗(p)	✗	✓(2.99)
**HB**	**COV-2**	**COV-2**	**K353**	**I353**	**S353**	**T353**	**D353**	**R353**	**W353**
s-s	N501	Q498	✓(3.02)	✓(2.74)	✗	✓(3.01)	✗	✗	✓(3.46)
s-s	Y449	Q498	✓(3.04)	✓(3.23)	✗	✗	✗	✓(2.97)	✗
**CI**	**COV-2**	**ACE2**	**K353**	**I353**	**S353**	**T353**	**D353**	**R353**	**W353**
vdW/h	Y505	X353	✓	✓	✓	✓	✓	✓	✓
p	Q498	Q42	✓	✗	✓	✓	✓	✗	✗
**CI**	**ACE2**	**ACE2**	**K353**	**I353**	**S353**	**T353**	**D353**	**R353**	**W353**
vdW/h	Y41	X353	✓	✓	✓(p)	✓(p)	✓	✓	✓

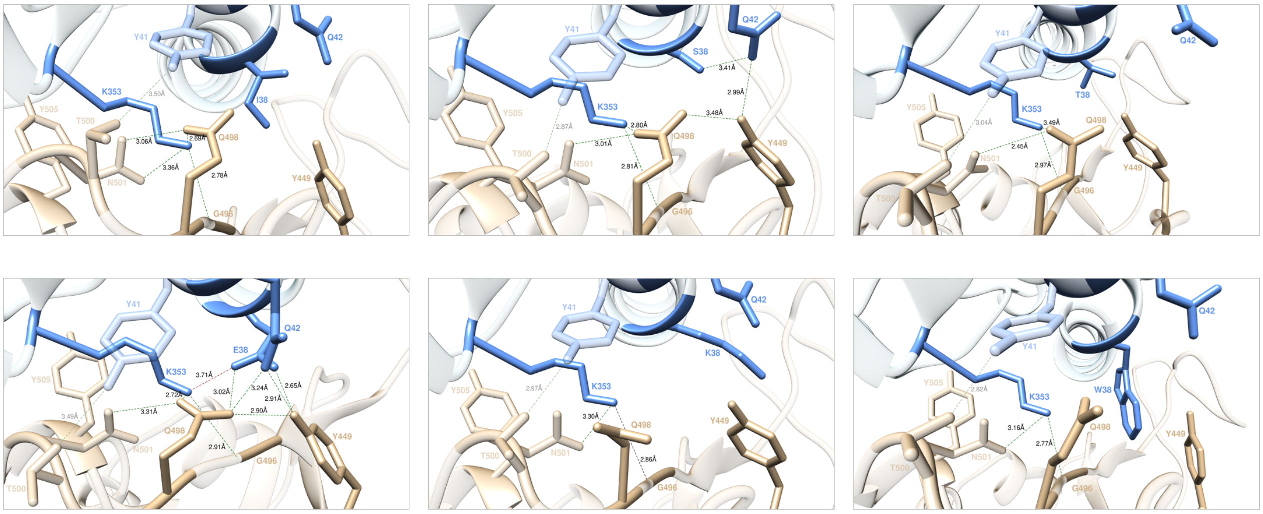

Figure S12. Main interactions involving the ACE2 I38 (top left), S38 (top middle), T38 (top right), E38 (bottom left), K38 (bottom middle) and W38 (bottom right) at the interface with S-RBD$_{CoV-2}$ as obtained from the corresponding equilibrated MD simulation s. The wild-type D38 is presented and discussed in the main text (Figure 10A). Colors and other explanations as in Figure S1. For further details see Tables S1 and S14.

Table S14. Main intermolecular and intramolecular interactions between the wild-type ACE2 residue D38 and all considered mutants at the protein-protein interface detected during MD simulations of ACE2 in complex with the RBD of SARS-CoV-2 (COV-2). Acronyms and other explanations as in Table S3.

**HB**	**COV-2**	**ACE2**	**D38**	**I38**	**S38**	**T38**	**E38**	**K38**	**W38**
s-s	Q498	K353	✓(2.87)	✓(2.69)	✓(2.80)	✓(3.49)	✓(2.72)	✗	✗
b-s	G496	K353	✓(2.95)	✓(2.78)	✓(2.81)	✓(2.97)	✓(2.91)	✓(2.86)	✓(2.77)
s-s	T500	Y41	✓(3.08)	✓(3.50)	✓(2.87)	✓(3.04)	✓(3.49)	✓(2.97)	✓(2.82)
s-s	N501	Y41	✓(3.23)	✗	✗(p)	✗(p)	✗(p)	✗(p)	✗(p)
s-s	Q498	X38	✓(2.92)	✗	✗	✗	✓(3.02)	✗	✗
s-s	N501	K353	✗(p)	✓(3.36)	✗(p)	✗(p)	✗(p)	✓(3.30)	✓(3.16)
s-s	Y449	X38	✓(2.92)	✗	✗	✗	✓(2.65)	✗	✗
s-s	Y449	Q42	✓(3.03)	✗	✓(2.99)	✗	✓(2.91)	✗	✗(p)
**SB**	**ACE2**	**ACE2**	**D38**	**I38**	**S38**	**T38**	**E38**	**K38**	**W38**
	K353	X38	✓(3.66)	✗	✗	✗	✓(3.71)	✗	✗
**HB**	**ACE2**	**ACE2**	**D38**	**I38**	**S38**	**T38**	**E38**	**K38**	**W38**
b-s	Q42	X38	✓(3.04)	✗	✓(3.41)	✗	✗(p)	✗(p)	✗
**HB**	**COV-2**	**COV-2**	**D38**	**I38**	**S38**	**T38**	**E38**	**K38**	**W38**
s-s	N501	Q498	✓(3.02)	✓(3.06)	✓(3.01)	✓(2.45)	✓(3.31)	✗	✗
s-s	Y449	Q498	✓(3.04)	✗	✓(3.48)	✗	✓(2.90)	✗	✗(p)
**CI**	**COV-2**	**ACE2**	**D38**	**I38**	**S38**	**T38**	**E38**	**K38**	**W38**
vdW/h	Q498	Y41	✓	✓	✓	✓(p)	✓	✓(p)	✗
p	Q498	Q42	✓	✗	✗	✗	✓	✗	✗

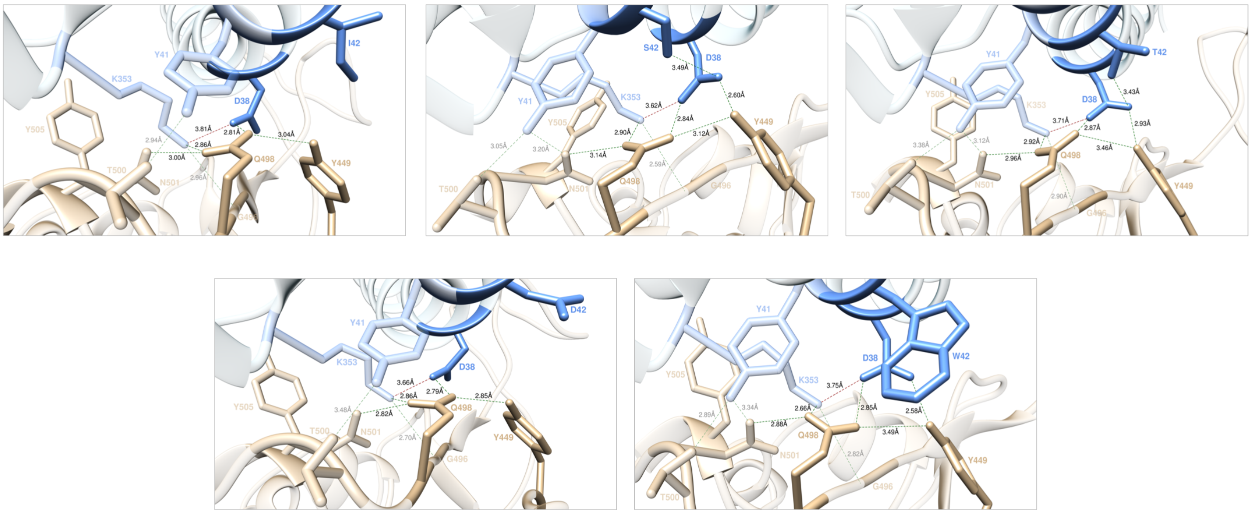

Figure S13. Main interactions involving the ACE2 I42 (top left), S42 (top middle), T42 (top right), D42 (bottom left), and W42 (bottom right) at the interface with S-RBD$_{CoV-2}$ as obtained from the corresponding equilibrated MD simulations. The wild-type Q42 and the K42 mutant are presented and discussed in the main text (Figure 10A-B). Colors and other explanations as in Figure S1. For further details see Tables S1 and S15.

Table S15. Main intermolecular and intramolecular interactions between the wild-type ACE2 residue Q42 and all considered mutants* at the protein-protein interface detected during MD simulations of ACE2 in complex with the RBD of SARS-CoV-2 (COV-2). Acronyms and other explanations as in Table S3. *Mutant K42 is discussed in detail in main text.

**HB**	**COV-2**	**ACE2**	**Q42**	**I42**	**S42**	**T42**	**D42**	**K42**	**W42**
s-s	Q498	K353	✓(2.87)	✓(2.86)	✓(2.90)	✓(2.92)	✓(2.86)	✓(2.70)	✓(2.66)
b-s	G496	K353	✓(2.95)	✓(2.96)	✓(2.59)	✓(2.90)	✓(2.70)	✓(2.84)	✓(2.82)
s-s	T500	Y41	✓(3.08)	✓(2.94)	✓(3.05)	✓(3.38)	✓(3.48)	✓(3.12)	✓(2.89)
s-s	N501	Y41	✓(3.23)	✗(p)	✓(3.20)	✓(3.12)	✗(p)	✓(3.37)	✓(3.34)
s-s	Q498	D38	✓(2.92)	✓(2.81)	✓(2.84)	✓(2.87)	✓(2.79)	✓(2.91)	✓(2.85)
s-s	Y449	D38	✓(2.92)	✗	✓(2.60)	✓(2.93)	✗	✓(3.26)	✓(2.58)
s-s	Y449	X42	✓(3.03)	✗	✗(p)	✗(p)	✗	✓(2.43)	✗
**SB**	**ACE2**	**ACE2**	**Q42**	**I42**	**S42**	**T42**	**D42**	**K42**	**W42**
	K353	D38	✓(3.66)	✓(3.81)	✓(3.62)	✓(3.71)	✓(3.66)	✓(3.73)	✓(3.75)
**HB**	**ACE2**	**ACE2**	**Q42**	**I42**	**S42**	**T42**	**D42**	**K42**	**W42**
b-s	D38	X42	✓(3.04)	✗	✓(s-s,3.49)	✓(s-s,3.43)	✗	✓(SB,3.91)	✗
**HB**	**COV-2**	**COV-2**	**Q42**	**I42**	**S42**	**T42**	**D42**	**K42**	**W42**
s-s	N501	Q498	✓(3.02)	✓(3.00)	✓(3.14)	✓(2.96)	✓(2.82)	✓(2.98)	✓(2.88)
s-s	Y449	Q498	✓(3.04)	✓(3.04)	✓(3.12)	✓(3.46)	✓(2.85)	✓(3.20)	✓(3.49)
**CI**	**COV-2**	**ACE2**	**Q42**	**I42**	**S42**	**T42**	**D42**	**K42**	**W42**
p	N501	K353	✓	✓	✓	✓	✓	✓	✓
vdW/h	Q498	Y41	✓	✓(p)	✓	✓	✓(p)	✓	✓
p	Q498	X42	✓	✗	✓	✓	✗	✓	✗

The qualitative *in silico*/experimental data comparison reported in Figure 11C (main text) for the mutagenesis of ACE Q42 into I42 shows that a stabilizing effect is reported by experiment[4] while an interface disrupting effect is anticipated *in silico* ($\Delta\Delta G_{ACE2}$(Q42I) = -2.46 ± 0.10 kcal/mol, Table S1). The analysis of the corresponding MD trajectory for the ACE2 I42 mutant in complex with the S-RBD$_{CoV-2}$ (Figure S13 (top left) and Table S15) reveals that, with respect to the wild-

type assembly, several important inter- and intramolecular interaction are no longer established in the presence of this mutant residue, that is: i) the three intermolecular HBs between the viral Y449 and ACE2 D38 and I42, and between the viral N501 and ACE2 Y41, respectively; ii) the internal HB between D38 and I42; and iii) the intermolecular CI with the viral N498. Also, the experimental results contextually show a slightly positive effect for valine while the opposite results for alanine (in agreement with our previous findings).[3] Accordingly, we retain that a polar-to-hydrophobic substitution at this receptor position ultimately exerts a mildly destabilizing effects on the relevant ACE2/S-RBD$_{CoV-2}$ complex formation.

Moreover, as it can be seen comparing the image for the wild-type Q42 (Figure 10A in main text) and those for the S42 and T42 mutants (Figure S13, top middle and right panel, respectively), according to the present MD simulations these three ACE2 residues can engage the same type and number of intra- and intermolecular interactions at the corresponding receptor/S-RBD$_{COV-2}$ interface. Thus, for both S42 and T42 a neutral effect is predicted ($\Delta\Delta G_{ACE2}$(Q42S) = -0.08 ± 0.06 kcal/mol and $\Delta\Delta G_{ACE2}$(Q42T) = +0.10 ± 0.11 kcal/mol, respectively, Figure 11C in main text and Table S1). Interestingly, *in silico* and *in vitro* data do coincide for the T42 mutant while experiment reveals a slightly interface disrupting behavior for the S42 ACE2 isoform[4] (Figure 11C). Given the uncertainty underlying both computational and experimental techniques, the arbitrariness in the quantification range of stabilizing/destabilizing effects, and the MD-based evidence supporting the fact S42 and T42 can form the same interface interactions, we retain that these two alternative residues are both equivalent to the wild-type ACE2 Q42 and, as such, have a neutral effect on the relative protein/protein interface.

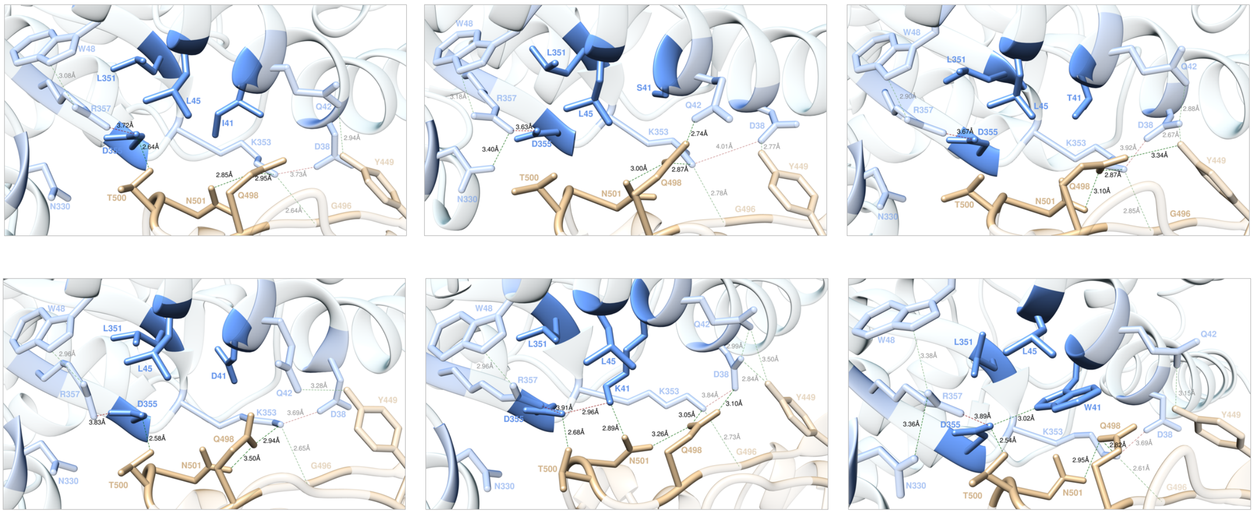

Figure S14. Main interactions involving the ACE2 I41 (top left), S41 (top middle), T41 (top right), D41 (bottom left), K41 (bottom middle) and W41 (bottom right) at the interface with S-RBD$_{CoV-2}$ as obtained from the corresponding equilibrated MD simulations. The wild-type Y41 is presented and discussed in the main text (Figure 12A). Colors and other explanations as in Figure S1. For further details see Tables S1 and S16.

Table S16. Main intermolecular and intramolecular interactions between the wild-type ACE2 residue Y41 and all considered mutants at the protein-protein interface detected during MD simulations of ACE2 in complex with the RBD of SARS-CoV-2 (COV-2). Acronyms and other explanations as in Table S3.

HB	COV-2	ACE2	Y41	I41	S41	T41	D41	K41	W41
s-s	T500	X41	✓(3.08)	✗	✗	✗	✗	✗	✗
s-s	N501	X41	✓(3.23)	✗	✗	✗	✗	✓(2.89)	✗
s-s	T500	D355	✓(2.77)	✓(2.64)	✗(p)	✗(p)	✓(2.58)	✓(2.68)	✓(2.54)
s-s	Q498	K353	✓(2.87)	✓(2.95)	✓(2.87)	✓(2.87)	✓(2.94)	✓(3.05)	✓(2.82)
b-s	G496	K353	✓(2.95)	✓(2.64)	✓(2.78)	✓(2.85)	✓(2.65)	✓(2.73)	✓(2.61)
s-s	Q498	D38	✓(2.92)	✗(p)	✗	✗(p)	✗	✓(3.10)	✗(p)
s-s	Y449	D38	✓(2.92)	✗	✓(2.77)	✓(2.67)	✗(p)	✓(2.84)	✗(p)
s-s	Y449	Q42	✓(3.03)	✓(2.94)	✗(p)	✗(p)	✓(3.28)	✓(3.50)	✓(3.15)
**SB**	**ACE2**	**ACE2**	**Y41**	**I41**	**S41**	**T41**	**D41**	**K41**	**W41**
	R357	D355	✓(3.68)	✓(3.72)	✓(3.63)	✓(3.67)	✓(3.83)	✓(3.91)	✓(3.89)
	K353	D38	✓(3.66)	✓(3.73)	✓(4.01)	✓(3.92)	✓(3.69)	✓(3.84)	✓(3.69)
**HB**	**ACE2**	**ACE2**	**Y41**	**I41**	**S41**	**T41**	**D41**	**K41**	**W41**
s-s	D355	X41	✓(2.78)	✗	✗	✗	✗	✓(2.96)	✓(3.02)
b-s	D38	Q42	✓(3.04)	✗	✗(p)	✓(s-s,2.88)	✗(p)	✓(s-s,2.99)	✗
s-s	W48	R357	✓(2.61)	✓(3.08)	✓(3.18)	✓(2.90)	✓(2.96)	✓(2.96)	✓(3.38)
**HB**	**COV-2**	**COV-2**	**Y41**	**I41**	**S41**	**T41**	**D41**	**K41**	**W41**
s-s	N501	Q498	✓(3.02)	✓(2.85)	✓(3.00)	✓(3.10)	✓(3.50)	✓(3.26)	✓(2.95)
s-s	Y449	Q498	✓(3.04)	✗(p)	✗(p)	✓(3.34)	✗	✗(p)	✗(p)
**CI**	**COV-2**	**ACE2**	**Y41**	**I41**	**S41**	**T41**	**D41**	**K41**	**W41**
vdW/h	Q498	X41	✓	✓	✗	✓	✓	✓	✓
p	N501	K353	✓	✓	✓	✓	✓	✓	✓

p	Q498	Q42	✓	✗	✓(HB,2.74)	✗	✓	✗	✗
p	T500	R357	✓	✓	✓	✓	✓	✗	✓
vdw/h	T500	N330	✓	✓	✓	✓	✓	✓	✓
**CI**	**ACE2**	**ACE2**	**Y41**	**I41**	**S41**	**T41**	**D41**	**K41**	**W41**
vdW/h	K353	X41	✓	✓	✗	✓	✓	✓	✓
vdW/h	L45	X41	✓	✓	✗	✓	✓	✓	✓
vdW/h	L351	X41	✓	✗	✗	✗	✗	✓	✓
vdW/h	L351	R357	✓	✓	✓	✓	✓	✓	✓
vdW/h	N330	R357	✓	✓	✓(HB,3.40)	✗	✓	✓	✓(HB,3.36)

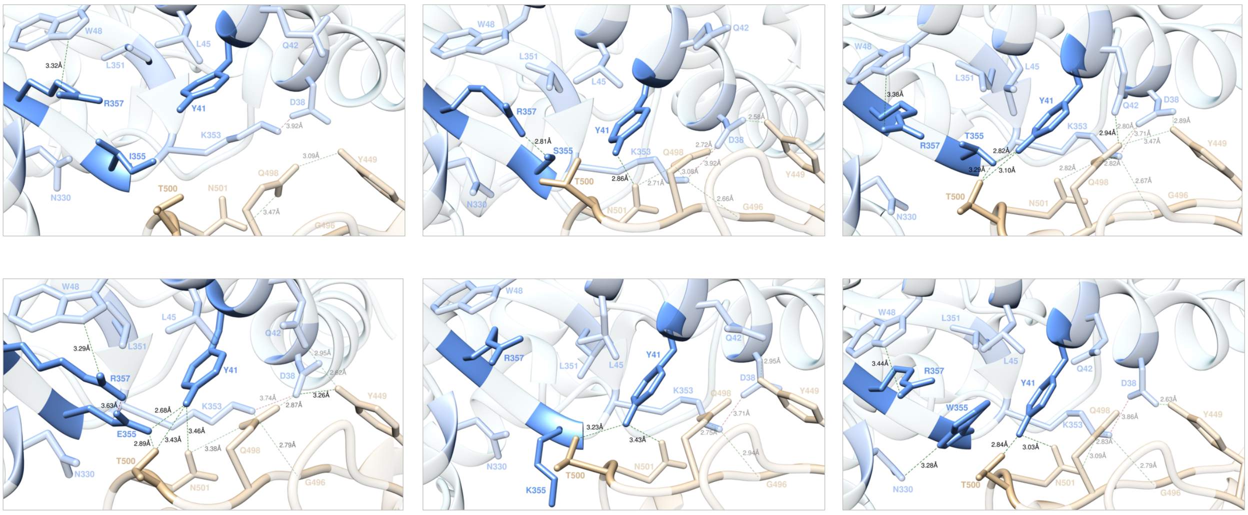

Figure S15. Main interactions involving the ACE2 I355 (top left), S355 (top middle), T355 (top right), E355 (bottom left), K355 (bottom middle) and W355 (bottom right) at the interface with S-RBD$_{CoV-2}$ as obtained from the corresponding equilibrated MD simulations. The wild-type D355 is presented and discussed in the main text (Figure 12A). Colors and other explanations as in Figure S1. For further details see Tables S1 and S17.

Table S17. Main intermolecular and intramolecular interactions between the wild-type ACE2 residue D355 and all considered mutants at the protein-protein interface detected during MD simulations of ACE2 in complex with the RBD of SARS-CoV-2 (COV-2). Acronyms and other explanations as in Table S3.

**HB**	**COV-2**	**ACE2**	**D355**	**I355**	**S355**	**T355**	**E355**	**K355**	**W355**
s-s	T500	Y41	✓(3.08)	✗	✗	✓(3.10)	✓(3.43)	✓(3.23)	✓(2.84)
s-s	N501	Y41	✓(3.23)	✗	✓(2.86)	✗	✓(3.46)	✓(3.43)	✓(3.03)
s-s	T500	X355	✓(2.77)	✗	✗(p)	✓(3.29)	✓(2.89)	✗	✗
s-s	Q498	K353	✓(2.87)	✗(p)	✓(3.08)	✓(2.82)	✗(p)	✓(2.75)	✓(2.83)
b-s	G496	K353	✓(2.95)	✗	✓(2.66)	✓(2.67)	✓(2.79)	✓(2.94)	✓(2.79)
s-s	Q498	D38	✓(2.92)	✗(p)	✓(2.72)	✓(2.80)	✓(2.87)	✗(p)	✗(p)
s-s	Y449	D38	✓(2.92)	✗(p)	✓(2.58)	✓(2.89)	✓(3.26,2.62)	✗(p)	✓(2.63)
s-s	Y449	Q42	✓(3.03)	✗	✗	✗(p)	✗(p)	✓(2.95)	✗
**SB**	**ACE2**	**ACE2**	**D355**	**I355**	**S355**	**T355**	**E355**	**K355**	**W355**
	R357	X355	✓(3.68)	✗	✓(HB,2.81)	✗	✓(3.63)	✗	✗
	K353	D38	✓(3.66)	✓(3.92)	✓(3.92)	✓(3.71)	✓(3.74)	✓(3.71)	✓(3.86)
**HB**	**ACE2**	**ACE2**	**D355**	**I355**	**S355**	**T355**	**E355**	**K355**	**W355**
s-s	Y41	X355	✓(2.78)	✗	✗	✓(2.82)	✓(2.68)	✗	✗
b-s	D38	Q42	✓(3.04)	✗	✗	✗(p)	✓(2.95)	✗	✗
s-s	W48	R357	✓(2.61)	✓(3.32)	✗(p)	✓(3.38)	✓(3.29)	✗	✓(3.44)
s-s	N330	X355	✗	✗	✗	✗	✗	✗	✓(3.28)
**HB**	**COV-2**	**COV-2**	**D355**	**I355**	**S355**	**T355**	**E355**	**K355**	**W355**
s-s	N501	Q498	✓(3.02)	✓(3.47)	✓(2.71)	✓(2.82)	✓(3.38)	✗(p)	✓(3.09)
s-s	Y449	Q498	✓(3.04)	✓(3.09)	✗(p)	✓(3.47)	✗(p)	✗(p)	✗(p)
**CI**	**COV-2**	**ACE2**	**D355**	**I355**	**S355**	**T355**	**E355**	**K355**	**W355**
vdW/h	Q498	Y41	✓	✗	✓	✓	✓	✓	✓

p	N501	K353	✓	✗	✓	✓	✓	✗	✓
p	Q498	Q42	✓	✗	✗	✓(HB,2.94)	✓	✓	✗
p	T500	R357	✓	✗	✓	✗	✓	✗	✗
vdw/h	T500	N330	✓	✗	✗	✓	✓	✓	✓
**CI**	**ACE2**	**ACE2**	**D355**	**I355**	**S355**	**T355**	**E355**	**K355**	**W355**
vdW/h	L351	R357	✓	✓	✓	✗	✓	✓	✓
vdW/h	N330	R357	✓	✓(p)	✓	✓	✓	✗	✗

At variance with experiment[4] for which a negative effect is foreseen for the ACE2 D355E mutation (Figure 13B, main text), our calculations predict a neutral effect corresponding to a $\Delta\Delta G_{ACE2}$(D355E) value of -0.06 ± 0.11 kcal/mol (Table S1). In line with this, the inspection of the bottom left panel in Figure S15 and the list of interactions reported in Table S17 reveals that all main intermolecular contacts across the protein-protein binding interface seen in the wild-type complex are preserved, the double HB between D38 and Y449 in the mutant isoform compensating for the weaker polar interaction between the receptor K353 and the viral protein Q498. Accordingly, we retain that the conservative D355E substitution has no significant effect on the affinity of the mutant ACE2 for the receptor binding domain of the viral S-protein.

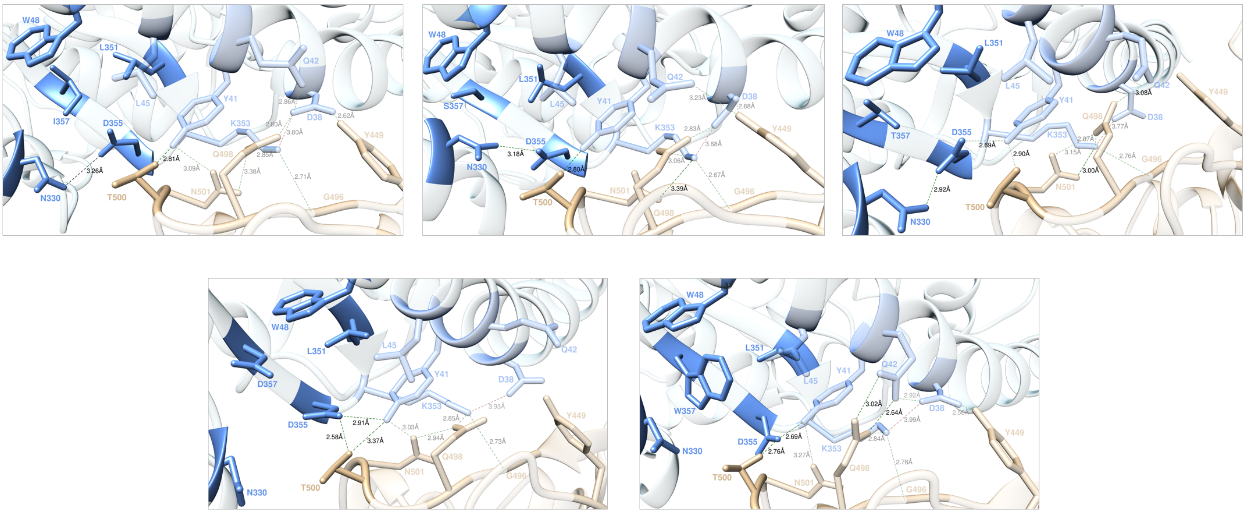

Figure S16. Main interactions involving the ACE2 I357 (top left), S357 (top middle), T357 (top right), D357 (bottom left), and W357 (bottom right) at the interface with S-RBD$_{CoV-2}$ as obtained from the corresponding equilibrated MD simulations. The wild-type R357 and the K357 mutant are presented and discussed in the main text (Figure 12A-B). Colors and other explanations as in Figure S1. For further details see Tables S1 and S18.

Table S18. Main intermolecular and intramolecular interactions between the wild-type ACE2 residue R357 and all considered mutants* at the protein-protein interface detected during MD simulations of ACE2 in complex with the RBD of SARS-CoV-2 (COV-2). Acronyms and other explanations as in Table S3. *Mutant K357 is discussed in detail in main text.

HB	COV-2	ACE2	R357	I357	S357	T357	D357	K357	W357
s-s	T500	Y41	✓(3.08)	✓(2.81)	✓(2.80)	✓(2.90)	✓(3.37)	✓(3.36)	✗
s-s	N501	Y41	✓(3.23)	✓(3.09)	✗(p)	✗(p)	✓(3.03)	✓(2.81)	✓(3.27)
s-s	T500	D355	✓(2.77)	✗(p)	✗(p)	✗	✓(2.58)	✓(2.73)	✓(2.76)
s-s	Q498	K353	✓(2.87)	✓(2.85)	✓(3.06)	✓(2.87)	✓(2.85)	✓(2.71)	✓(2.84)
b-s	G496	K353	✓(2.95)	✓(2.71)	✓(2.67)	✓(2.76)	✓(2.73)	✓(2.80)	✓(2.76)
s-s	Q498	D38	✓(2.92)	✓(2.80)	✓(2.83)	✗(p)	✗(p)	✓(3.37)	✗(p)
s-s	Y449	D38	✓(2.92)	✓(2.62)	✓(2.68)	✗(p)	✗(p)	✓(3.10)	✓(2.56)
s-s	Y449	Q42	✓(3.03)	✗(p)	✓(3.23)	✗(p)	✗(p)	✓(2.98)	✗
**SB**	**ACE2**	**ACE2**	**R357**	**I357**	**S357**	**T357**	**D357**	**K357**	**W357**
	D355	X357	✓(3.68)	✗	✗	✗	✗	✓(3.63,3.77)	✗
	K353	D38	✓(3.66)	✓(3.80)	✓(3.68)	✓(3.77)	✓(3.93)	✓(3.82)	✓(3.99)
**HB**	**ACE2**	**ACE2**	**R357**	**I357**	**S357**	**T357**	**D357**	**K357**	**W357**
s-s	Y41	D355	✓(2.78)	✗	✗(p)	✓(2.69)	✓(2.91)	✓(2.89)	✓(2.69)
b-s	D38	Q42	✓(3.04)	✓(s-s,2.86)	✗(p)	✗(p)	✗(p)	✓(s-s,2.90)	✓(s-s,2.92)
s-s	W48	X357	✓(2.61)	✗	✗(p)	✗	✗	✗(p)	✗(π/π)
s-s	N330	D355	✗	✓(3.26)	✓(3.18)	✓(2.92)	✗	✗	✗
**HB**	**COV-2**	**COV-2**	**R357**	**I357**	**S357**	**T357**	**D357**	**K357**	**W357**
s-s	N501	Q498	✓(3.02)	✓(3.38)	✗(p)	✓(3.15)	✓(2.94)	✗(p)	✗
s-s	Y449	Q498	✓(3.04)	✗(p)	✗(p)	✗(p)	✗(p)	✓(3.40)	✗
**CI**	**COV-2**	**ACE2**	**R357**	**I357**	**S357**	**T357**	**D357**	**K357**	**W357**
vdW/h	Q498	Y41	✓	✓	✓	✓	✓	✓	✓

p	N501	K353	✓	✓	✓(HB,3.39)	✓(HB,3.00)	✓	✗	✓
p	Q498	Q42	✓	✓	✓	✓(HB,3.08)	✓	✓	✓(HB,2.64,3.02)
p	T500	X357	✓	✗	✗	✗	✗	✓	✗
vdw/h	T500	N330	✓	✓	✓	✓	✗	✓	✓
**CI**	**ACE2**	**ACE2**	**R357**	**I357**	**S357**	**T357**	**D357**	**K357**	**W357**
vdW/h	L351	X357	✓	✗	✗	✗	✗	✓	✓
vdW/h	N330	X357	✓	✗	✓	✓	✗	✗	✓

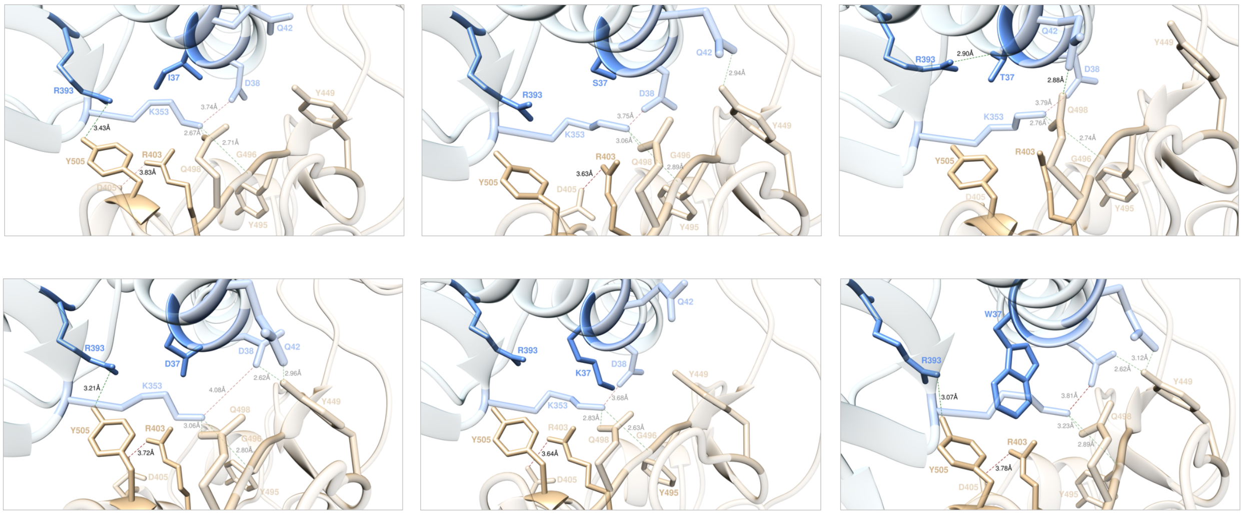

Figure S17. Main interactions involving the ACE2 I37 (top left), S37 (top middle), T37 (top right), D37 (bottom left), K37 (bottom middle) and W37 (bottom right) at the interface with S-RBD$_{CoV-2}$ as obtained from the corresponding equilibrated MD simulations. The wild-type E37 is presented and discussed in the main text (Figure 14A). Colors and other explanations as in Figure S1. For further details see Tables S1 and S19.

Table S19. Main intermolecular and intramolecular interactions between the wild-type ACE2 residue E37 and all considered mutants at the protein-protein interface detected during MD simulations of ACE2 in complex with the RBD of SARS-CoV-2 (COV-2). Acronyms and other explanations as in Table S3.

HB	COV-2	ACE2	E37	I37	S37	T37	D37	K37	W37
s-s	Y505	X37	✓(3.15)	✗	✗	✗	✗	✗	✗
s-s	Q498	K353	✓(2.87)	✓(2.67)	✓(3.06)	✓(2.76)	✓(3.06)	✓(2.83)	✓(3.23)
b-s	G496	K353	✓(2.95)	✓(2.71)	✓(2.89)	✓(2.74)	✓(2.80)	✓(2.63)	✓(2.89)
s-s	Q498	D38	✓(2.92)	✗(p)	✗(p)	✗(p)	✗(p)	✗(p)	✗(p)
s-s	Y449	D38	✓(2.92)	✗	✗	✗	✓(2.62)	✗	✓(2.62)
s-s	Y449	Q42	✓(3.03)	✗	✓(2.94)	✗	✓(2.96)	✗	✓(3.12)
**SB**	**COV2**	**ACE2**	**E37**	**I37**	**S37**	**T37**	**D37**	**K37**	**W37**
	R403	X37	✓(3.62)	✗	✗	✗	✗	✗	✗
**SB**	**ACE2**	**ACE2**	**E37**	**I37**	**S37**	**T37**	**D37**	**K37**	**W37**
	R393	X37	✓(3.93,3.63)	✗	✗	✗(HB,2.90)	✗	✗	✗
	K353	D38	✓(3.66)	✓(3.74)	✓(3.75)	✓(3.79)	✓(4.08)	✓(3.68)	✓(3.81)
**SB**	**COV2**	**COV2**	**E37**	**I37**	**S37**	**T37**	**D37**	**K37**	**W37**
	D405	R403	✓(3.95)	✓(3.83)	✓(3.63)	✗	✓(3.72)	✓(3.64)	✓(3.78)
**HB**	**ACE2**	**ACE2**	**E37**	**I37**	**S37**	**T37**	**D37**	**K37**	**W37**
b-s	D38	Q42	✓(3.04)	✗	✗	✗(p)	✗(p)	✗	✗(p)
**HB**	**COV-2**	**COV-2**	**E37**	**I37**	**S37**	**T37**	**D37**	**K37**	**W37**
s-s	Y449	Q498	✓(3.04)	✗	✗	✗	✗(p)	✗(p)	✗(p)
**CI**	**COV-2**	**ACE2**	**E37**	**I37**	**S37**	**T37**	**D37**	**K37**	**W37**
p	Y505	R393	✓	✓(HB,3.43)	✗	✗	✓(HB,3.21)	✗	✓(HB,3.07)
p	Q498	Q42	✓	✗	✗	✓(HB,2.88)	✓	✗	✓
**CI**	**COV2**	**COV2**	**E37**	**I37**	**S37**	**T37**	**D37**	**K37**	**W37**
π/c	Y505	R403	✓	✗	✗	✗	✓	✓	✗

vdW/h	Y495	R403	✓	✓	✓	✓	✓	✗	✓

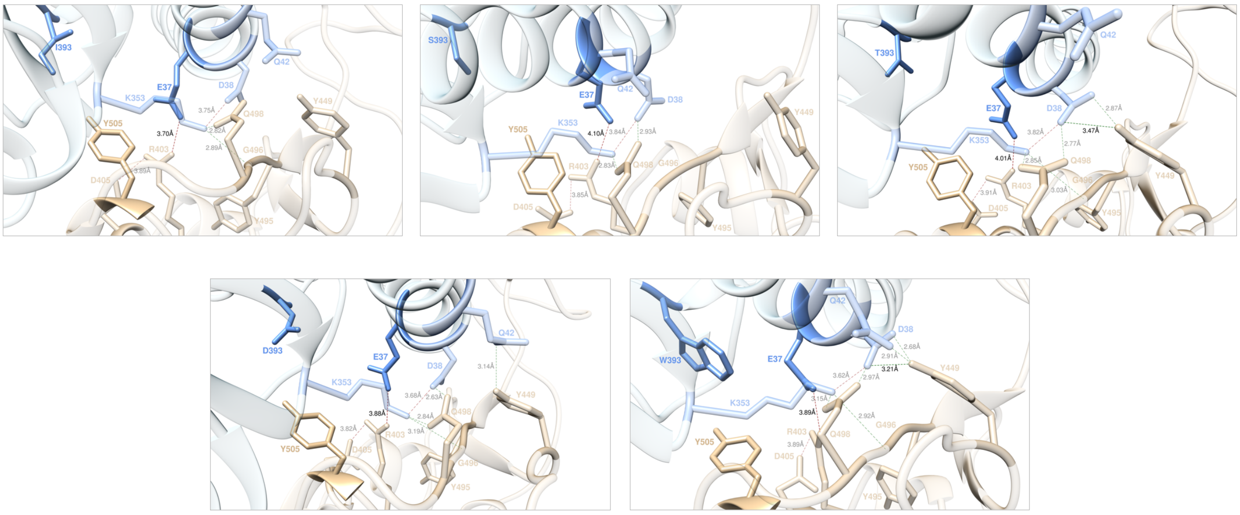

Figure S18. Main interactions involving the ACE2 I393 (top left), S393 (top middle), T393 (top right), D393 (bottom left), and W393 (bottom right) at the interface with S-RBD$_{CoV-2}$ as obtained from the corresponding equilibrated MD simulations. The wild-type R393 and the R393K mutant are presented and discussed in the main text (Figure 14A-B). Colors and other explanations as in Figure S1. For further details see Tables S1 and S20.

Table S20. Main intermolecular and intramolecular interactions between the wild-type ACE2 residue R393 and all considered mutants* at the protein-protein interface detected during MD simulations of ACE2 in complex with the RBD of SARS-CoV-2 (COV-2). Acronyms and other explanations as in Table S3. *Mutant K393 is discussed in detail in main text.

HB	COV-2	ACE2	R393	I393	S393	T393	D393	K393	W393
s-s	Y505	E37	✓(3.15)	✗	✗	✗	✗	✓(2.84)	✗
s-s	Q498	K353	✓(2.87)	✓(2.82)	✓(2.83)	✓(2.85)	✓(2.84)	✓(2.75)	✓(3.15)
b-s	G496	K353	✓(2.95)	✓(2.89)	✗	✓(3.03)	✓(3.19)	✓(2.78)	✓(2.92)
s-s	Q498	D38	✓(2.92)	✗(p)	✓(2.93)	✓(2.77)	✓(2.63)	✓(2.77)	✓(2.97)
s-s	Y449	D38	✓(2.92)	✗	✗	✓(2.87,3.47)	✗	✓(2.83)	✓(2.68,3.21)
s-s	Y449	Q42	✓(3.03)	✗(p)	✗	✗	✓(3.14)	✓(3.17)	✓(2.91)
**SB**	**COV2**	**ACE2**	**R393**	**I393**	**S393**	**T393**	**D393**	**K393**	**W393**
	R403	E37	✓(3.62)	✓(3.70)	✓(4.10)	✓(4.01)	✓(3.88)	✓(3.84)	✓(3.89)
**SB**	**ACE2**	**ACE2**	**R393**	**I393**	**S393**	**T393**	**D393**	**K393**	**W393**
	E37	X393	✓(3.93,3.63)	✗	✗	✗	✗	✓(3.73)	✗
	K353	D38	✓(3.66)	✓(3.75)	✓(3.75)	✓(3.82)	✓(3.68)	✓(3.63)	✓(3.62)
**SB**	**COV2**	**COV2**	**R393**	**I393**	**S393**	**T393**	**D393**	**K393**	**W393**
	D405	R403	✓(3.95)	✓(3.89)	✓(3.84)	✓(3.91)	✓(3.82)	✓(3.74)	✓(3.89)
**HB**	**ACE2**	**ACE2**	**R393**	**I393**	**S393**	**T393**	**D393**	**K393**	**W393**
b-s	D38	Q42	✓(3.04)	✗	✗(p)	✗	✗	✓(s-s,2.80)	✗(p)
**HB**	**COV-2**	**COV-2**	**R393**	**I393**	**S393**	**T393**	**D393**	**K393**	**W393**
s-s	Y449	Q498	✓(3.04)	✗	✗	✗	✗	✓(3.29)	✗(p)
**CI**	**COV-2**	**ACE2**	**R393**	**I393**	**S393**	**T393**	**D393**	**K393**	**W393**
p	Y505	X393	✓	✗	✗	✗	✗	✓(HB,2.84)	✗
p	Q498	Q42	✓	✗(p)	✗	✗	✗	✗	✓
**CI**	**COV2**	**COV2**	**R393**	**I393**	**S393**	**T393**	**D393**	**K393**	**W393**

π/c	Y505	R403	✓	✓	✓	✓	✗	✓	✗
vdW/h	Y495	R403	✓	✓	✓	✗	✓	✓	✓

According to our calculations, when the ACE2 wild-type R393 is replaced by a threonine, a moderate destabilizing effect at the corresponding protein/protein binding interface is observed ($\Delta\Delta G_{ACE2}$(R393T) = -1.98 ± 0.08 kcal/mol, Figure 15B in main text and Table S1), whilst a neutral effect is experimentally reported.[4] As seen from the interaction list in Table S20, the T393 mutant receptor fails to engage the side chains of the two S-RBD_{CoV-2} residues Y505 and Y449 in the two intermolecular HBs with ACE2 E37 and Q42 seen in the wild-type complex, respectively, alongside with some missing inter- and intramolecular CIs (Figures 14A and S18 (top right panel)).

References

1. Shang, J.; Ye, G.; Shi, K.; Wan, Y.; Luo, C.; Aihara, H.; Geng, Q.; Auerbach, A.; Li, F., Structural Basis of Receptor Recognition by SARS-CoV-2. *Nature* **2020,** *581*, 221-224.

2. Lan, J.; Ge, J.; Yu, J.; Shan, S.; Zhou, H.; Fan, S.; Zhang, Q.; Shi, X.; Wang, Q.; Zhang, L.; Wang, X., Structure of the SARS-CoV-2 Spike Receptor-Binding Domain Bound to the ACE2 Receptor. *Nature* **2020,** *581*, 215-220.

3. Laurini, E.; Marson, D.; Aulic, S.; Fermeglia, M.; Pricl, S., Computational Alanine Scanning and Structural Analysis of the SARS-CoV-2 Spike Protein/Angiotensin-Converting Enzyme 2 Complex. *ACS Nano* **2020**, *14*, 11821-11830.

4. Chan, K. K.; Dorosky, D.; Sharma, P.; Abbasi, S. A.; Dye, J. M.; Kranz, D. M.; Herbert, A. S.; Procko, E., Engineering Human ACE2 to Optimize Binding to the Spike Protein of SARS Coronavirus 2. *Science* **2020,** *369*, 1261-1265.
