# Supplementary material for: Computational Mutagenesis at the SARS-CoV-2 Spike Protein/Angiotensin-Converting Enzyme 2 Binding Interface: Comparison with Experimental Evidence
Source: ACS Nano. 2021 Mar 18;15(4):6929–48. doi: 10.1021/acsnano.0c10833 (PMC8009103; doi:10.1021/acsnano.0c10833)
Supplement: Supplementary file 2 — nn0c10833_si_002.pdf [file nn0c10833_si_002.pdf]

Supporting information – Part 2 for the paper:

Computational Mutagenesis at the SARS-CoV-2 Spike Protein/Angiotensin-Converting Enzyme 2 Binding Interface: Comparison with Experimental Evidences

Erik Laurini[1,‡], Domenico Marson[1,‡], Suzana Aulic[1], Alice Fermeglia[1], Sabrina Pricl[1,2*]

[1]Molecular Biology and Nanotechnology Laboratory (MolBNL@UniTS), DEA, University of Trieste, 34127 Trieste, Italy
[2]Department of General Biophysics, Faculty of Biology and Environmental Protection, University of Lodz, 90-136 Lodz, Poland

Table S2. Relative binding free energy and its components calculated by the combined computational mutagenesis for the S-RBD of SARS-CoV-2 residues effectively involved in the binding interface with the ACE2 (see the SI Materials and Methods section for details). IE = interaction entropy. $\Delta\Delta G = \Delta G_{WT} - \Delta G_{MUT}$ (see text for details).

	**R403I**	**R403S**	**R403T**	**R403D**	**R403K**	**R403W**		**K417I**	**K417S**	**K417T**	**K417D**	**K417R**	**K417W**
$\Delta\Delta E_{DISP}$	-1.58	-1.15	-1.01	-1.87	-0.17	-0.25	$\Delta\Delta E_{vdW}$	-0.43	-1.36	-1.25	-1.19	0.39	0.42
$\Delta\Delta E_{ELE}$	-0.86	-0.42	-0.45	-3.48	0.01	-1.84	$\Delta\Delta E_{ELE}$	-0.51	-0.11	-0.08	-2.01	-0.62	-0.83
$\Delta\Delta H$	-2.44	-1.57	-1.46	-5.35	-0.16	-2.09	$\Delta\Delta H$	-0.94	-1.47	-1.33	-3.20	-0.23	-0.41
$\Delta\Delta IE$	-1.25	-0.53	-0.47	-0.98	-0.05	-0.82	$\Delta\Delta IE$	-0.25	-0.27	-0.2	-0.79	-0.02	0.07
**$\Delta\Delta G_{ACE2}$**	**-3.69**	**-2.10**	**-1.93**	**-6.33**	**-0.21**	**-2.91**	**$\Delta\Delta G_{ACE2}$**	**-1.19**	**-1.74**	**-1.53**	**-3.99**	**-0.25**	**-0.34**
	**(0.16)**	**(0.08)**	**(0.13)**	**(0.15)**	**(0.03)**	**(0.19)**		**(0.17)**	**(0.08)**	**(0.19)**	**(0.10)**	**(0.08)**	**(0.09)**

	**Y449I**	**Y449S**	**Y449T**	**Y449D**	**Y449K**	**Y449W**		**Y453I**	**Y453S**	**Y453T**	**Y453D**	**Y453K**	**Y453W**
$\Delta\Delta E_{DISP}$	-1.34	-1.21	-1.09	-0.79	-1.33	-1.07	$\Delta\Delta E_{DISP}$	0.29	-0.32	-0.11	-0.62	0.62	0.36
$\Delta\Delta E_{ELE}$	-1.53	-1.40	-1.07	-0.73	-0.74	-1.58	$\Delta\Delta E_{ELE}$	-1.35	-0.60	-1.11	-2.22	-0.28	-0.46
$\Delta\Delta H$	-2.87	-2.61	-2.16	-1.52	-2.07	-2.65	$\Delta\Delta H$	-1.06	-0.92	-1.22	-2.84	+0.34	-0.10
$\Delta\Delta IE$	-0.12	-0.15	-0.18	-0.03	-0.06	-0.54	$\Delta\Delta IE$	-0.11	-0.18	-0.16	-0.21	-0.15	-0.04
**$\Delta\Delta G_{ACE2}$**	**-2.99**	**-2.76**	**-2.34**	**-1.55**	**-2.13**	**-3.19**	**$\Delta\Delta G_{ACE2}$**	**-1.17**	**-1.10**	**-1.38**	**-3.05**	**0.19**	**-0.14**
	**(0.18)**	**(0.09)**	**(0.16)**	**(0.15)**	**(0.11)**	**(0.13)**		**(0.14)**	**(0.12)**	**(0.05)**	**(0.10)**	**(0.07)**	**(0.17)**

	**L455I**	**L455S**	**L455T**	**L455D**	**L455K**	**L455W**		**F456I**	**F456S**	**F456T**	**F456D**	**F456K**	**F456W**
$\Delta\Delta E_{DISP}$	0.03	0.07	-0.42	-0.94	-0.23	0.56	$\Delta\Delta E_{DISP}$	-0.97	-1.51	-1.32	-1.32	0.61	0.33
$\Delta\Delta E_{ELE}$	-0.01	0.14	0.17	2.51	-2.81	0.32	$\Delta\Delta E_{ELE}$	-0.03	0.06	0.09	-1.22	1.33	0.41
$\Delta\Delta H$	0.02	0.21	-0.25	1.57	-3.04	0.88	$\Delta\Delta H$	-1.00	-1.45	-1.23	-2.54	1.94	0.74
$\Delta\Delta IE$	0.00	-0.02	-0.03	0.12	-0.17	0.07	$\Delta\Delta IE$	0.02	-0.04	-0.02	-0.03	0.12	0.15
**$\Delta\Delta G_{ACE2}$**	**0.02**	**0.19**	**-0.28**	**1.69**	**-3.21**	**0.95**	**$\Delta\Delta G_{ACE2}$**	**-0.98**	**-1.49**	**-1.25**	**-2.57**	**2.06**	**0.89**
	**(0.05)**	**(0.06)**	**(0.09)**	**(0.06)**	**(0.08)**	**(0.19)**		**(0.06)**	**(0.19)**	**(0.05)**	**(0.16)**	**(0.17)**	**(0.18)**

	**F486I**	**F486S**	**F486T**	**F486D**	**F486K**	**F486W**		**N487I**	**N487S**	**N487T**	**N487D**	**N487K**	**N487W**
$\Delta\Delta E_{DISP}$	-0.51	-0.96	-0.85	-1.28	-0.24	0.07	$\Delta\Delta E_{DISP}$	-0.94	-0.71	-1.07	-0.35	-1.06	0.21
$\Delta\Delta E_{ELE}$	0.07	0.12	0.11	-0.46	-0.57	0.10	$\Delta\Delta E_{ELE}$	-1.34	0.25	-0.30	-0.37	-0.84	-0.31
$\Delta\Delta H$	-0.44	-0.84	-0.74	-1.74	-0.81	0.17	$\Delta\Delta H$	-2.28	-0.46	-1.37	-0.72	-1.90	-0.10
$\Delta\Delta IE$	-0.02	-0.07	-0.05	-0.11	-0.07	0.01	$\Delta\Delta IE$	-0.11	-0.05	-0.06	-0.06	-0.08	-0.09
**$\Delta\Delta G_{ACE2}$**	**-0.46**	**-0.91**	**-0.79**	**-1.85**	**-0.88**	**0.18**	**$\Delta\Delta G_{ACE2}$**	**-2.39**	**-0.51**	**-1.43**	**-0.78**	**-1.98**	**-0.19**
	**(0.07)**	**(0.09)**	**(0.07)**	**(0.08)**	**(0.18)**	**(0.13)**		**(0.11)**	**(0.06)**	**(0.09)**	**(0.07)**	**(0.06)**	**(0.09)**

	**Y489I**	**Y489S**	**Y489T**	**Y489D**	**Y489K**	**Y489W**		**Q493I**	**Q493S**	**Q493T**	**Q493D**	**Q493K**	**Q493W**
$\Delta\Delta E_{DISP}$	-1.02	-2.29	-2.07	-1.77	-1.26	-0.14	$\Delta\Delta E_{DISP}$	-1.21	-0.92	-0.96	-0.96	0.12	-1.48
$\Delta\Delta E_{ELE}$	-0.56	-0.40	-0.71	-1.48	-2.06	-0.45	$\Delta\Delta E_{ELE}$	-1.27	0.18	0.24	-0.71	0.53	-1.19
$\Delta\Delta H$	-1.58	-2.69	-2.78	-3.25	-3.32	-0.59	$\Delta\Delta H$	-2.48	-0.74	-0.72	-1.67	0.65	-2.67
$\Delta\Delta IE$	-0.43	-0.53	-0.41	-0.62	-0.69	-0.09	$\Delta\Delta IE$	-0.29	-0.15	-0.19	-0.31	0.11	-0.21
**$\Delta\Delta G_{ACE2}$**	**-2.01**	**-3.22**	**-3.19**	**-3.87**	**-4.01**	**-0.68**	**$\Delta\Delta G_{ACE2}$**	**-2.77**	**-0.89**	**-0.91**	**-1.98**	**0.76**	**-2.88**
	**(0.17)**	**(0.19)**	**(0.07)**	**(0.15)**	**(0.12)**	**(0.08)**		**(0.19)**	**(0.12)**	**(0.17)**	**(0.09)**	**(0.11)**	**(0.18)**

	**Q498I**	**Q498S**	**Q498T**	**Q498D**	**Q498K**	**Q498W**		**T500I**	**T500S**	**T500T**	**T500D**	**T500K**	**T500W**
$\Delta\Delta E_{DISP}$	-2.23	-2.94	-3.01	-2.29	-2.24	-2.05	$\Delta\Delta E_{DISP}$	-1.88	-0.12	-	-0.55	-0.22	-1.03
$\Delta\Delta E_{ELE}$	-1.29	0.18	0.09	1.19	-1.38	-1.96	$\Delta\Delta E_{ELE}$	-1.39	-0.11	-	-1.12	-1.42	-0.32
$\Delta\Delta H$	-3.52	-2.76	-2.92	-1.11	-3.62	-4.01	$\Delta\Delta H$	-3.27	-0.23	-	-1.67	-1.64	-1.35
$\Delta\Delta IE$	-0.37	-0.25	-0.27	-0.05	-0.34	-0.17	$\Delta\Delta IE$	-0.31	0.03	-	-0.14	-0.03	-0.35
**$\Delta\Delta G_{ACE2}$**	**-3.89**	**-3.01**	**-3.19**	**-1.16**	**-3.96**	**-4.18**	**$\Delta\Delta G_{ACE2}$**	**-3.58**	**-0.20**	-	**-1.81**	**-1.67**	**-1.70**
	**(0.10)**	**(0.19)**	**(0.11)**	**(0.07)**	**(0.17)**	**(0.11)**		**(0.13)**	**(0.06)**		**(0.09)**	**(0.12)**	**(0.05)**

	**N501I**	**N501S**	**N501T**	**N501D**	**N501K**	**N501W**		**Y505I**	**Y505S**	**Y505T**	**Y505D**	**Y505K**	**Y505W**
$\Delta\Delta E_{DISP}$	-0.98	-0.53	-0.22	-0.85	-0.31	-0.15	$\Delta\Delta E_{DISP}$	-1.89	-1.92	-2.03	-2.12	-1.91	0.42
$\Delta\Delta E_{ELE}$	-0.68	0.27	0.35	0.68	-6.07	-1.38	$\Delta\Delta E_{ELE}$	-1.05	-0.33	-0.57	0.09	-0.17	0.13
$\Delta\Delta H$	-1.66	-0.26	0.13	-0.17	-6.38	-1.53	$\Delta\Delta H$	-2.94	-2.25	-2.60	-2.03	-2.08	0.55
$\Delta\Delta IE$	-0.19	0.08	0.15	-0.02	-0.51	-0.08	$\Delta\Delta IE$	-0.32	-0.43	-0.38	-0.32	-0.25	0.09
**$\Delta\Delta G_{ACE2}$**	**-1.85**	**-0.18**	**0.28**	**-0.19**	**-6.89**	**-1.61**	**$\Delta\Delta G_{ACE2}$**	**-3.26**	**-2.68**	**-2.98**	**-2.35**	**-2.33**	**0.64**
	**(0.13)**	**(0.03)**	**(0.16)**	**(0.08)**	**(0.15)**	**(0.07)**		**(0.12)**	**(0.11)**	**(0.13)**	**(0.09)**	**(0.12)**	**(0.10)**

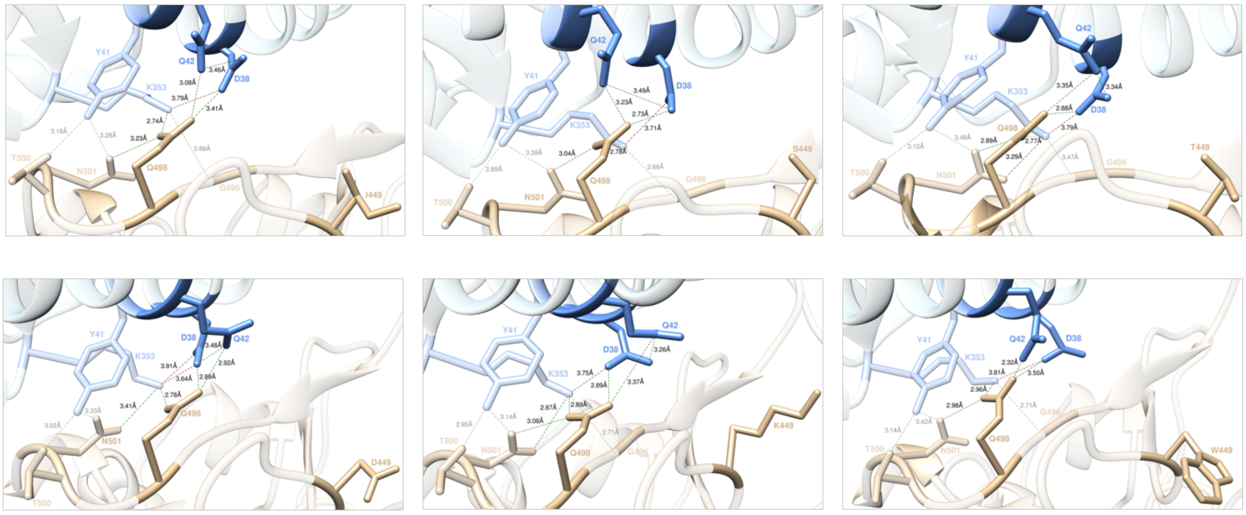

Figure S19. Main interactions involving the S-RBD$_{CoV-2}$ I449 (top left), S449 (top middle), T449 (top right), D449 (bottom left), K449 (bottom middle), and W449 (bottom right) at the interface with ACE2 as obtained from the corresponding equilibrated MD simulations. The wild-type residue Y449 is presented and discussed in the main text (Figure 10A). In this and all remaining Figures, the secondary structures of ACE2 and S-RBD$_{CoV-2}$ are portrayed as light blue and light sienna ribbons, respectively. Each protein residue under discussion and all other residues directly interacting with them are highlighted in dark matching-colored sticks and labelled; further residues/interactions related to the residue under investigation are evidenced in light matching-colored sticks and labelled in light gray. Hydrogen bonds and salt bridges are represented as dark green and dark red broken lines, respectively, and the relevant average distances are reported (in black) accordingly; new HBs and SBs detected in each mutant complex are also indicated using dark green/red broken lines and black labels. For further details see Tables S2 and S21.

Table S21. Main intermolecular and intramolecular interactions between the wild-type S-RBD$_{CoV-2}$ residue Y449 and all considered mutants at the protein-protein interface detected during MD simulations of ACE2 in complex with the RBD of SARS-CoV-2 (COV-2). HB = hydrogen bond; SB = salt bridge; CI = contact interactions, including van der Waals/hydrophobic (vdW/h), polar (p), π/π and π/cation (π/c) interactions. In the HB column, s-s indicates side chain-side chain interactions while s-b (or b-s) and b-b indicate side chain-backbone and backbone-backbone interactions, respectively. Preserved/new or lost interactions are marked with the symbols ✓ and ✗, respectively. Relevant changes in the type/nature of the interactions are indicated in parenthesis. For HBs and SBs, the relevant average lengths (in Å) are also reported (their standard deviations, all within 10%, are not shown for clarity). Charges eventually not involved in SBs at the protein/protein interface are also indicated.

**HB**	**COV-2**	**ACE2**	**Y449**	**I449**	**S449**	**T449**	**D449**	**K449**	**W449**
s-s	Q498	K353	✓(2.87)	✓(2.74)	✓(2.78)	✓(2.77)	✓(2.78)	✓(2.89)	✓(2.96)
s-s	Q498	D38	✓(2.92)	✓(3.41)	✓(2.73)	✓(2.88)	✓(2.89)	✓(2.89)	✓(3.50)
s-s	X449	D38	✓(2.92)	✗	✗	✗	✗	✗	✗
s-s	X449	Q42	✓(3.03)	✗	✗	✗	✗	✗	✗
s-s	N501	Y41	✓(3.23)	✓(3.28)	✓(3.39)	✓(3.48)	✓(3.33)	✓(3.14)	✓(3.42)
s-s	Q498	Q42	✗	✓(3.08)	✓(3.23)	✓(3.35)	✓(2.92)	✓(3.37)	✓(2.32)
s-s	T500	Y41	✓(3.08)	✓(3.18)	✓(2.88)	✓(3.12)	✓(3.02)	✓(2.95)	✓(3.14)
s-s	G496	K353	✓(2.95)	✓(2.86)	✓(2.66)	✓(3.47)	✗	✓(2.71)	✓(2.71)
**SB**	**ACE2**	**ACE2**	**Y449**	**I449**	**S449**	**T449**	**D449**	**K449**	**W449**
	K353	D38	✓(3.66)	✓(3.79)	✓(3.71)	✓(3.79)	✓(3.64,3.91)	✓(3.75)	✓(3.81)
**HB**	**ACE2**	**ACE2**	**Y449**	**I449**	**S449**	**T449**	**D449**	**K449**	**W449**
s-s	D38	Q42	✓(3.04)	✓(3.46)	✓(3.49)	✓(3.34)	✓(3.48)	✓(3.26)	✗(p)
**HB**	**COV-2**	**COV-2**	**Y449**	**I449**	**S449**	**T449**	**D449**	**K449**	**W449**

s-s	Q498	N501	✓(3.02)	✓(3.23)	✓(3.04)	✓(2.89)	✗(p)	✓(3.08)	✓(2.98)
s-s	X449	Q498	✓(3.04)	✗	✗	✗	✗	✗	✗
**CI**	**COV-2**	**ACE2**	**Y449**	**I449**	**S449**	**T449**	**D449**	**K449**	**W449**
p	N501	K353	✓	✓	✓	✓(HB,3.29)	✓(HB,3.41)	✓(HB,2.87)	✓
vdW/h	X449	D38	✓	✗	✗	✗	✗	✗	✗

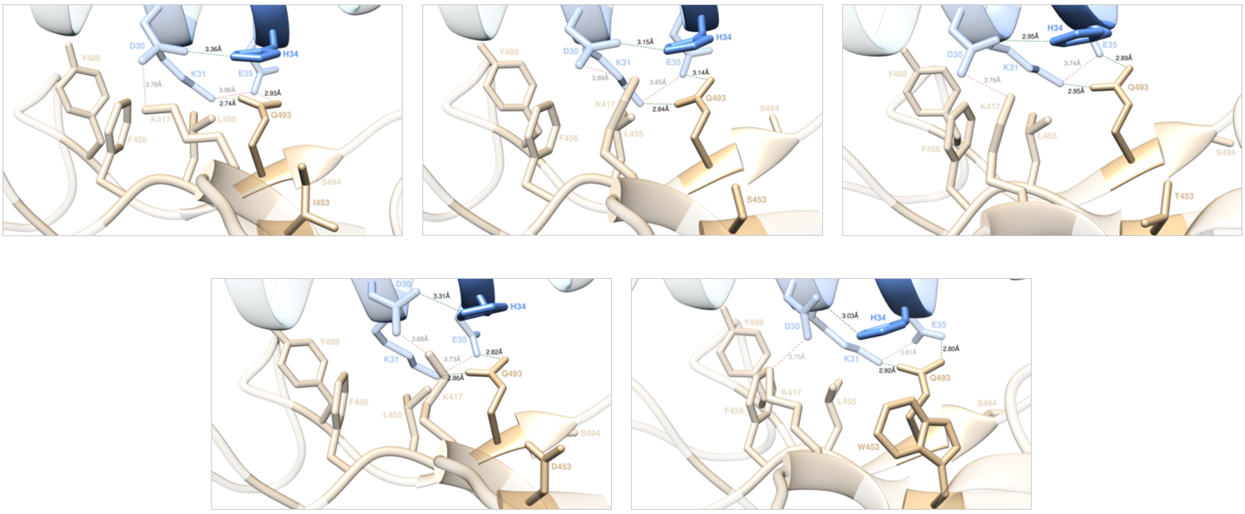

Figure S20. Main interactions involving the S-RBD$_{CoV-2}$ I453 (top left), S453 (top middle), T453 (top right), D453 (bottom left), and W453 (bottom right) at the interface with ACE2 as obtained from the corresponding equilibrated MD simulations. The wild-type residue Y453 and the K453 mutant are presented and discussed in the main text (Figure 4, panels A and D). Colors and other explanations as in Figure S19. For further details see Tables S2 and S22.

Table S22. Main intermolecular and intramolecular interactions between the wild-type S-RBD$_{CoV-2}$ residue Y453 and all considered mutants* at the protein-protein interface detected during MD simulations of ACE2 in complex with the RBD of SARS-CoV-2 (COV-2). Acronyms and other explanations as in Table 19. *Mutant K453 is discussed in detail in main text.

SB	COV-2	ACE2	Y453	I453	S453	T453	D453	K453	W453
	K417	D30	✓(3.85)	✓(3.76)	✓(3.89)	✓(3.76)	✓(3.88)	✓(3.69)	✓(3.75)
**HB**	**COV-2**	**ACE2**	**Y453**	**I453**	**S453**	**T453**	**D453**	**K453**	**W453**
s-s	Q493	K31	✓(3.04)	✓(2.74)	✓(2.84)	✓(2.95)	✓(2.86)	✓(2.81)	✓(2.92)
s-s	Q493	E35	✓(2.94)	✓(2.93)	✓(3.14)	✓(2.89)	✓(2.82)	✓(2.95)	✓(2.80)
**SB**	**ACE2**	**ACE2**	**Y453**	**I453**	**S453**	**T453**	**D453**	**K453**	**W453**
	K31	E35	✓(3.94)	✓(3.96)	✓(3.85)	✓(3.74)	✓(3.73)	✓(3.87)	✓(3.81)
**HB**	**ACE2**	**ACE2**	**Y453**	**I453**	**S453**	**T453**	**D453**	**K453**	**W453**
s-s	D30	H34	✓(3.31)	✓(3.36)	✓(3.15)	✓(2.95)	✓(3.31)	✓(2.96)	✓(3.03)
**HB**	**COV-2**	**COV-2**	**Y453**	**I453**	**S453**	**T453**	**D453**	**K453**	**W453**
s-s	Q493	S494	✓(3.24)	✗	✗(p)	✗	✗	✗(p)	✗
**CI**	**COV-2**	**ACE2**	**Y453**	**I453**	**S453**	**T453**	**D453**	**K453**	**W453**
p	X453	H34	✓	✗	✗	✗	✗	✓	✗
**CI**	**COV-2**	**COV-2**	**Y453**	**I453**	**S453**	**T453**	**D453**	**K453**	**W453**
vdW/h	X453	Q493	✓	✗	✗	✗	✗	✓(p)	✓

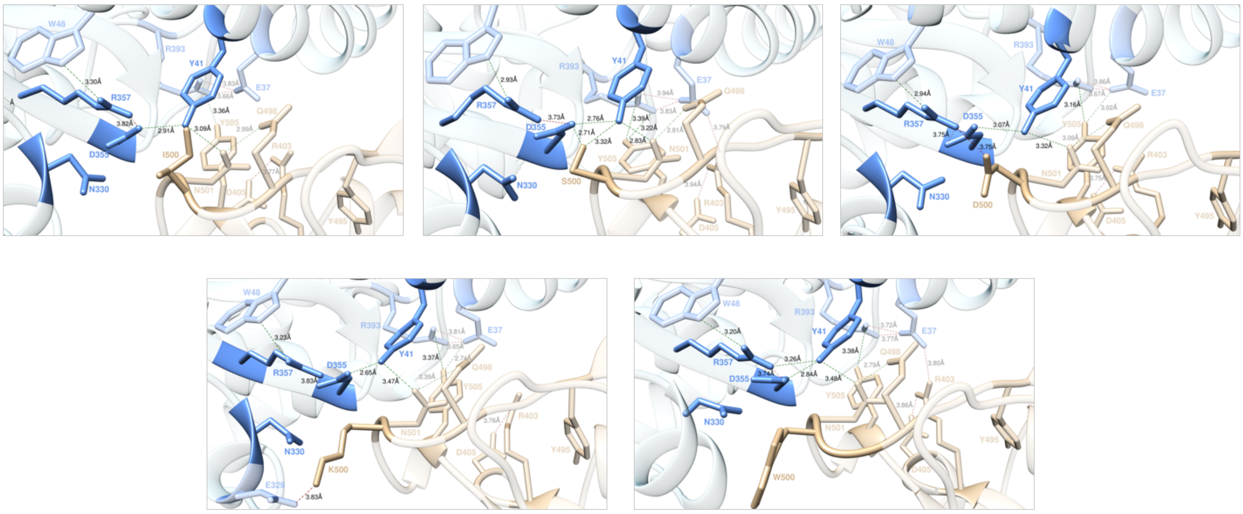

Figure S21. Main interactions involving the S-RBD$_{CoV-2}$ I500 (top left), S500 (top middle), D500 (top right), K500 (bottom left), and W500 (bottom right) at the interface with ACE2 as obtained from the corresponding equilibrated MD simulations. The wild-type residue T500 is presented and discussed in the main text (Figure 12A). Colors and other explanations as in Figure S19. For further details see Tables S2 and S23.

Table S23. Main intermolecular and intramolecular interactions between the wild-type S-RBD$_{CoV-2}$ residue T500 and all considered mutants at the protein-protein interface detected during MD simulations of ACE2 in complex with the RBD of SARS-CoV-2 (COV-2). Acronyms and other explanations as in Table 19.

**SB**	**COV-2**	**ACE2**	**T500**	**I500**	**S500**	**D500**	**K500**	**W500**
	R403	E37	✓(3.62)	✗(p)	✓(3.79)	✗(p)	✗(p)	✓(3.80)
	X500	R357	✗(p)	✗	✗(p)	✓(3.75)	✗	✗
	X500	E329	✗	✗	✗	✗	✓(3.83)	✗
**HB**	**COV-2**	**ACE2**	**T500**	**I500**	**S500**	**D500**	**K500**	**W500**
s-s	X500	Y41	✓(3.08)	✗	✓(3.32)	✗	✗	✗
s-s	X500	D355	✓(2.77)	✗	✓(2.71)	✗(p)	✗	✗
s-s	N501	Y41	✓(3.23)	✓(3.09)	✓(2.83)	✓(3.32)	✓(3.47)	✓(3.48)
s-s	Y505	E37	✓(3.15)	✗(p)	✗	✓(3.02)	✓(2.74)	✗(p)
s-s	Y505	R393	✗(p)	✓(3.36)	✓(3.22,3.39)	✓(3.16)	✓(3.37)	✓(3.38)
**SB**	**ACE2**	**ACE2**	**T500**	**I500**	**S500**	**D500**	**K500**	**W500**
	E37	R393	✓(3.69,3.93)	✓(3.66,3.83)	✓(3.83,3.94)	✓(3.67,3.86)	✓(3.65,3.81)	✓(3.72,3.77)
	R357	D355	✓(3.68)	✓(3.82)	✓(3.73)	✓(3.75)	✓(3.83)	✓(3.74)
**SB**	**COV-2**	**COV-2**	**T500**	**I500**	**S500**	**D500**	**K500**	**W500**
	R403	D405	✓(3.95)	✓(3.77)	✓(3.94)	✓(3.75)	✓(3.76)	✓(3.86)
**HB**	**ACE2**	**ACE2**	**T500**	**I500**	**S500**	**D500**	**K500**	**W500**
s-s	Y41	D355	✓(2.78)	✓(2.91)	✓(2.76)	✓(3.07)	✓(2.65)	✓(2.84)
s-s	W48	R357	✓(2.61)	✓(3.30)	✓(2.93)	✓(2.94)	✓(3.23)	✓(3.20)
s-s	Y41	R357	✗	✗	✗	✗	✗	✓(3.26)
**HB**	**COV-2**	**COV-2**	**T500**	**I500**	**S500**	**D500**	**K500**	**W500**
s-s	N501	Q498	✓(3.02)	✓(2.99)	✓(2.81)	✓(3.09)	✓(3.39)	✓(2.79)
**CI**	**COV-2**	**ACE2**	**T500**	**I500**	**S500**	**D500**	**K500**	**W500**
p	X500	N330	✓	✗	✗	✓	✓	✓

CI	COV-2	COV-2	T500	I500	S500	D500	K500	W500
π/c	R403	Y505	✓	✗	✗	✓	✗	✗(vdW/h)
vdW/h	R403	Y495	✓	✓	✗	✓	✓	✓

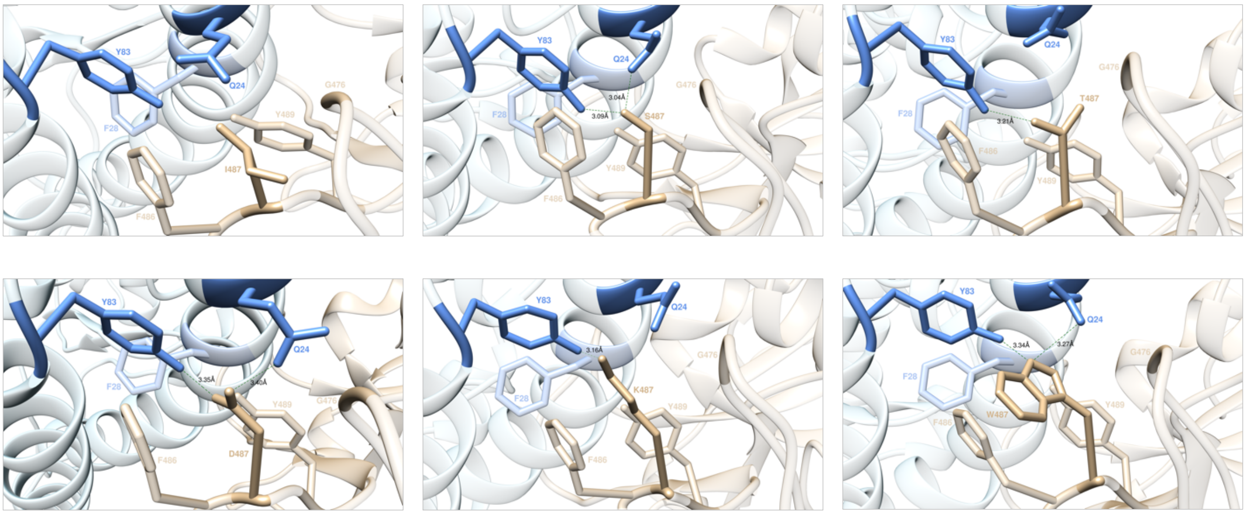

Figure S22. Main interactions involving the S-RBD$_{CoV-2}$ I487 (top left), S487 (top middle), T487 (top right), D487 (bottom left), K487 (bottom middle), and W487 (bottom right) at the interface with ACE2 as obtained from the corresponding equilibrated MD simulations. The wild-type residue N487 is presented and discussed in the main text (Figures 2A and 6A). Colors and other explanations as in Figure S19. For further details see Tables S2 and S24.

Table S24. Main intermolecular and intramolecular interactions between the wild-type S-RBD$_{CoV-2}$ residue N487 and all considered mutants at the protein-protein interface detected during MD simulations of ACE2 in complex with the RBD of SARS-CoV-2 (COV-2). Acronyms and other explanations as in Table 19.

**HB**	**COV-2**	**ACE2**	**N487**	**I487**	**S487**	**T487**	**D487**	**K487**	**W487**
s-s	X487	Y83	✓(3.03)	✗	✓(3.09)	✓(3.21)	✓(3.35)	✓(3.16)	✓(3.34)
s-s	X487	Q24	✓(2.88)	✗	✓(3.04)	✗(p)	✓(3.40)	✗(p)	✓(3.27)
**CI**	**COV-2**	**ACE2**	**N487**	**I487**	**S487**	**T487**	**D487**	**K487**	**W487**
p	Y489	Y83	✓	✓	✓	✓	✓	✗	✓
vdW/h	F486	Y83	✓	✗	✓	✓	✓	✗	✗
vdW/h	G476	Q24	✓	✓	✓	✗	✓	✓	✓
**CI**	**ACE2**	**ACE2**	**N487**	**I487**	**S487**	**T487**	**D487**	**K487**	**W487**
vdW/h	F28	Y83	✓	✓	✓	✓	✓	✓	✓

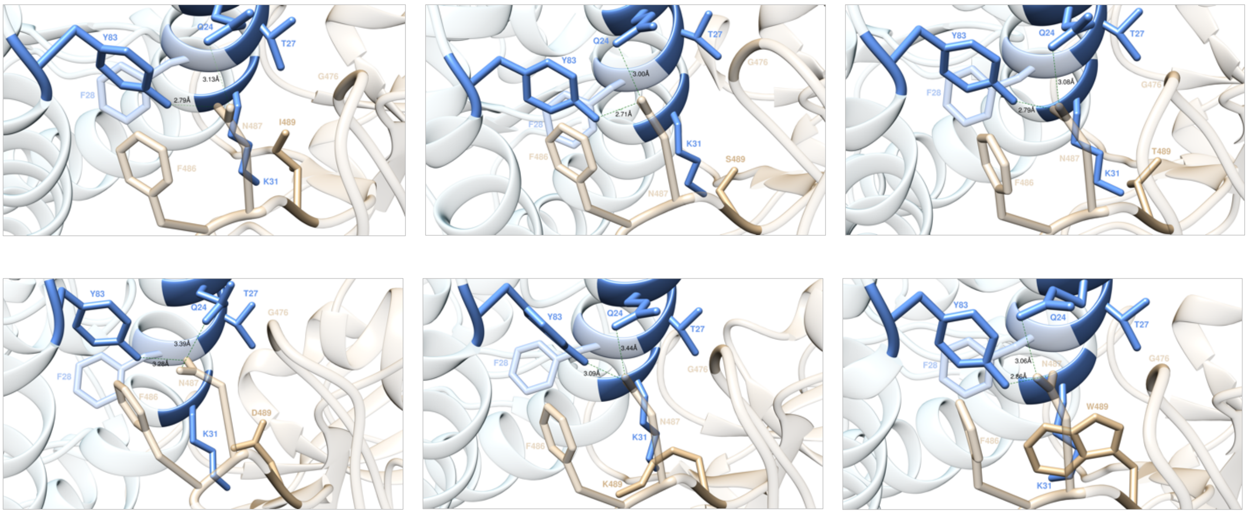

Figure S23. Main interactions involving the S-RBD$_{CoV-2}$ I489 (top left), S489 (top middle), T489 (top right), D489 (bottom left), K489 (bottom middle), and W489 (bottom right) at the interface with ACE2 as obtained from the corresponding equilibrated MD simulations. The wild-type residue Y489 is presented and discussed in the main text (Figure 2A,C). Colors and other explanations as in Figure S19. For further details see Tables S2 and S25.

Table S25. Main intermolecular and intramolecular interactions between the wild-type S-RBD$_{CoV-2}$ residue Y489 and all considered mutants at the protein-protein interface detected during MD simulations of ACE2 in complex with the RBD of SARS-CoV-2 (COV-2). Acronyms and other explanations as in Table 19.

**HB**	**COV-2**	**ACE2**	**Y489**	**I489**	**S489**	**T489**	**D489**	**K489**	**W489**
s-s	N487	Y83	✓(3.03)	✓(2.79)	✓(2.71)	✓(2.79)	✓(3.28)	✓(3.09)	✓(2.86)
s-s	N487	Q24	✓(2.88)	✓(3.13)	✓(3.00)	✓(3.08)	✓(3.39)	✓(3.44)	✓(3.06)
**CI**	**COV-2**	**ACE2**	**Y489**	**I489**	**S489**	**T489**	**D489**	**K489**	**W489**
p	X489	Y83	✓	✗	✗	✗	✗	✗	✓
vdW/h	X489	Q24	✓	✗	✗	✗	✗	✗	✗
vdW/h	X489	T27	✓	✓	✗	✓	✗	✗	✗
vdW/h	X489	K31	✓	✓	✓	✓	✓(p)	✗	✓
vdW/h	X489	Y83	✓	✗	✗	✗	✗	✗	✓
vdW/h	F486	Y83	✓	✓	✓	✓	✓	✓	✓
vdW/h	G476	Q24	✓	✗	✗	✗	✓	✓	✓
**CI**	**ACE2**	**ACE2**	**Y489**	**I489**	**S489**	**T489**	**D489**	**K489**	**W489**
vdW/h	F28	Y83	✓	✓	✓	✓	✓	✓	✓

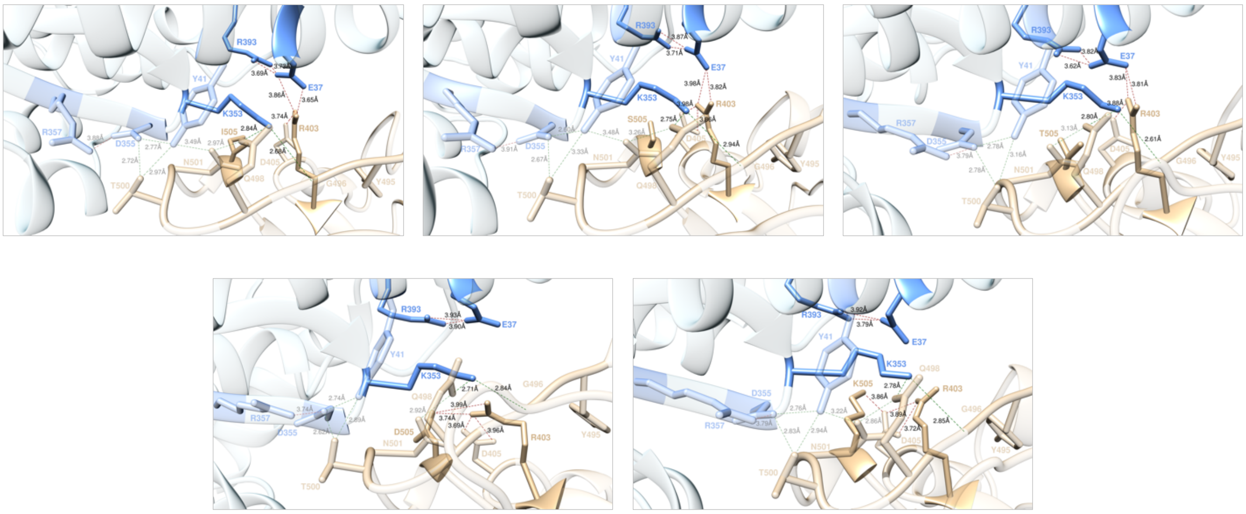

Figure S24. Main interactions involving the S-RBD$_{CoV-2}$ I505 (top left), S505 (top middle), T505 (top right), D505 (bottom left), and K505 (bottom right) at the interface with ACE2 as obtained from the corresponding equilibrated MD simulations. The wild-type residue Y505 and the W505 mutant are presented and discussed in the main text (Figures 14A and 17D). Colors and other explanations as in Figure S19. For further details see Tables S2 and S26.

Table S26. Main intermolecular and intramolecular interactions between the wild-type S-RBD$_{CoV-2}$ residue Y505 and all considered mutants* at the protein-protein interface detected during MD simulations of ACE2 in complex with the RBD of SARS-CoV-2 (COV-2). Acronyms and other explanations as in Table 19. *Mutant W505 is discussed in detail in main text.

**SB**	**COV-2**	**ACE2**	**Y505**	**I505**	**S505**	**T505**	**D505**	**K505**	**W505**
	R403	E37	✓(3.62)	✓(3.65,3.86)	✓(3.82,3.98)	✓(3.81,3.83)	✗	✗(p)	✓(3.63,3.87)
**HB**	**COV-2**	**ACE2**	**Y505**	**I505**	**S505**	**T505**	**D505**	**K505**	**W505**
s-s	X505	E37	✓(3.15)	✗	✗	✗	✗	✗	✓(2.84)
s-s	T500	Y41	✓(3.08)	✓(2.97)	✓(3.33)	✓(3.16)	✓(2.89)	✓(2.94)	✓(3.21)
s-s	T500	D355	✓(2.77)	✓(2.72)	✓(2.67)	✓(2.78)	✓(2.62)	✓(2.83)	✓(2.75)
s-s	N501	Y41	✓(3.23)	✓(3.49)	✓(3.48)	✗(p)	✗(p)	✓(3.22)	✓(3.49)
s-s	Q498	K353	✓(2.78)	✓(2.84)	✓(2.75)	✓(2.80)	✓(2.71)	✓(2.78)	✓(2.82)
s-s	G496	K353	✓(2.95)	✓(2.68)	✓(2.94)	✓(2.61)	✓(2.84)	✓(2.85)	✓(2.76)
**SB**	**ACE2**	**ACE2**	**Y505**	**I505**	**S505**	**T505**	**D505**	**K505**	**W505**
	E37	R393	✓(3.69,3.93)	✓(3.69,3.73)	✓(3.71,3.87)	✓(3.62,3.82)	✓(3.90,3.93)	✓(3.79,3.92)	✓(3.76,3.82)
	R357	D355	✓(3.68)	✓(3.88)	✓(3.91)	✓(3.79)	✓(3.74)	✓(3.79)	✓(3.81)
**SB**	**COV-2**	**COV-2**	**Y505**	**I505**	**S505**	**T505**	**D505**	**K505**	**W505**
	R403	D405	✓(3.95)	✓(3.74)	✓(3.86,3.98)	✓(3.88)	✓(3.69,3.96)	✓(3.72,3.89)	✓(3.75,4.02)
	D405	X505	✗	✗	✗	✗	✗	✓(3.86)	✗
**HB**	**ACE2**	**ACE2**	**Y505**	**I505**	**S505**	**T505**	**D505**	**K505**	**W505**
s-s	Y41	D355	✓(2.78)	✓(2.77)	✓(2.60)	✓(2.78)	✓(2.74)	✓(2.76)	✓(2.71)
**HB**	**COV-2**	**COV-2**	**Y505**	**I505**	**S505**	**T505**	**D505**	**K505**	**W505**
s-s	N501	Q498	✓(3.02)	✓(2.97)	✓(3.26)	✓(3.13)	✓(2.62)	✓(2.86)	✓(2.79)
**CI**	**COV-2**	**ACE2**	**Y505**	**I505**	**S505**	**T505**	**D505**	**K505**	**W505**
p	X505	R393	✓	✗	✗	✗	✗	✗	✓(HB,3.10)
vdW/h	X505	K353	✓						

CI	COV-2	COV-2	Y505	I505	S505	T505	D505	K505	W505
π/c	R403	X505	✓	✗	✗	✗	✓(SB,3.74,3.99)	✗	✗
vdW/h	R403	Y495	✓	✗	✗	✗	✗	✓	✓

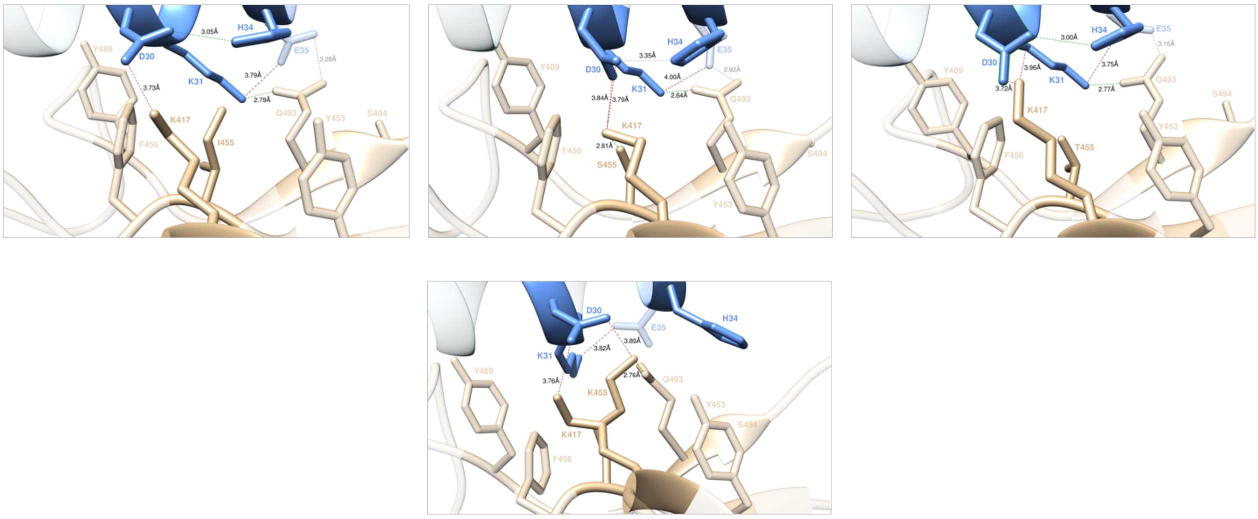

Figure S25. Main interactions involving the S-RBD$_{CoV-2}$ I455 (top left), S455 (top middle), T455 (top right), and K455 (bottom) at the interface with ACE2 as obtained from the corresponding equilibrated MD simulations. The wild-type residue L455 and the D455 and W455 mutants are presented and discussed in the main text (Figures 4A, 8A and 19A,B). Colors and other explanations as in Figure S19. For further details see Tables S2 and S27.

Table S27. Main intermolecular and intramolecular interactions between the wild-type S-RBD$_{CoV-2}$ residue L455 and all considered mutants* at the protein-protein interface detected during MD simulations of ACE2 in complex with the RBD of SARS-CoV-2 (COV-2). Acronyms and other explanations as in Table 19. *Mutants D455 and W455 are discussed in detail in main text.

**SB**	**COV-2**	**ACE2**	**L455**	**I455**	**S455**	**T455**	**D455**	**K455**	**W455**
	K417	D30	✓(3.85)	✓(3.73)	✓(3.79,3.84)	✓(3.72,3.96)	✓(3.75)	✓(3.76)	✓(3.92)
**HB**	**COV-2**	**ACE2**	**L455**	**I455**	**S455**	**T455**	**D455**	**K455**	**W455**
s-s	Q493	K31	✓(3.04)	✓(2.79)	✓(2.64)	✓(2.77)	✓(2.86)	✗(p)	✓(2.96)
s-s	Q493	E35	✓(2.94)	✓(3.28)	✓(2.92)	✓(3.16)	✓(2.87)	✗(p)	✓(2.74)
**SB**	**ACE2**	**ACE2**	**L455**	**I455**	**S455**	**T455**	**D455**	**K455**	**W455**
	K31	E35	✓(3.94)	✓(3.79)	✓(4.00)	✓(3.75)	✓(3.92)	✓(3.82)	✓(3.89)
**HB**	**ACE2**	**ACE2**	**L455**	**I455**	**S455**	**T455**	**D455**	**K455**	**W455**
s-s	D30	H34	✓(3.31)	✓(3.05)	✓(3.35)	✓(3.00)	✓(3.36)	✗	✓(3.07)
**HB**	**COV-2**	**COV-2**	**L455**	**I455**	**S455**	**T455**	**D455**	**K455**	**W455**
s-s	Q493	S494	✓(3.24)	✗(p)	✗	✗(p)	✗	✗	✓(2.88)
s-s	X455	Q493	✗	✗	✗	✗	✗	✓(2.76)	✗(p)
**CI**	**COV-2**	**ACE2**	**L455**	**I455**	**S455**	**T455**	**D455**	**K455**	**W455**
vdW/h	X455	D30	✓	✗	✗	✗	✗	✓(SB,3.89)	✓(HB,2.94)
vdW/h	X455	K31	✓	✓	✗	✗	✓(p)	✗	✓(p)
vdW/h	X455	H34	✓	✓	✗	✗	✓(HB,2.81)	✗	✓
p	Y453	H34	✓	✓	✓	✓	✓(HB,3.44)	✓	✓(HB,3.21)
vdW/h	Y489	K31	✓	✓	✓	✓	✗	✓	✓
**CI**	**COV-2**	**COV-2**	**L455**	**I455**	**S455**	**T455**	**D455**	**K455**	**W455**
vdW/h	X455	K417	✓	✓	✓(HB,2.81)	✓	✓(SB,3.74)	✗	✓
π/c	F456	K417	✓	✓	✓	✗	✓	✗	✗

According to the relevant MD simulations, mutating L455 into serine allows all wild-type interactions to be preserved across the relevant binding interface (Figure 4A in main text and Figure S25, top middle panel). Moreover, in the presence of S455 a stable, bifurcated salt-bridge (3.79 ± 0.15 Å and 3.84 ± 0.18 Å) instead of an isolated one is detected between K147 on S-RBD$_{CoV-2}$ and D30 on ACE2 (Figure 4A in main text and Figure S25, top middle panel. As discussed during the analysis of the ACE mutants (see Supporting Information-Part1.pdf), neutralizing the charges of the lysines is a key factor in the binding of coronavirus RBDs to ACE2.[1] Moreover, as reported by Lan *et al.*[2] and further verified in our previous work,[3] K417 is the only residue on the S-RBD$_{CoV-2}$ able to form an intermolecular SB with ACE D30. In addition, S455 itself further engages K147 in an internal HB (2.81 ± 0.21 Å). Therefore, the loss of some intermolecular CIs coupled with the more efficient lysine charge switch-off in the formation of the ACE2/S-RBD$_{CoV-2}$ complex in our opinion support the neutral effect of the S455 substitution ($\Delta\Delta G_{CoV-2}$(L455S) = +0.19 ± 0.06, Table S2).

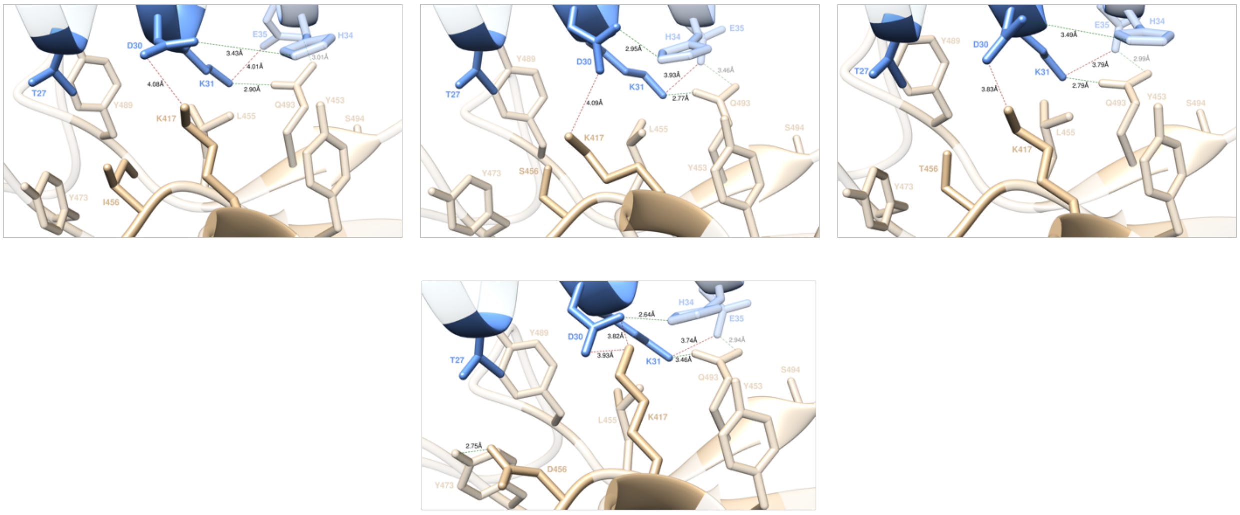

Figure S26. Main interactions involving the S-RBD$_{CoV-2}$ I456 (top left), S456 (top middle), T456 (top right), and D455 (bottom) at the interface with ACE2 as obtained from the corresponding equilibrated MD simulations. The wild-type residue F456 and the K456 and W456 mutants are presented and discussed in the main text (Figures 4A, 8A, and 19C,D). Colors and other explanations as in Figure S19. For further details see Tables S2 and S28.

Table S28. Main intermolecular and intramolecular interactions between the wild-type S-RBD$_{CoV-2}$ residue F456 and all considered mutants* at the protein-protein interface detected during MD simulations of ACE2 in complex with the RBD of SARS-CoV-2 (COV-2). Acronyms and other explanations as in Table 19. *Mutants K456 and W456 are discussed in detail in main text.

**SB**	**COV-2**	**ACE2**	**F456**	**I456**	**S456**	**T456**	**D456**	**K456**	**W456**
	K417	D30	✓(3.85)	✓(4.08)	✓(4.09)	✓(3.83)	✓(3.82,3.93)	✓(3.86)	✓(3.79)
	X456	E23	✗	✗	✗	✗	✗	✓(4.00)	✗
**HB**	**COV-2**	**ACE2**	**F456**	**I456**	**S456**	**T456**	**D456**	**K456**	**W456**
s-s	Q493	K31	✓(3.04)	✓(2.90)	✓(2.77)	✓(2.79)	✓(3.46)	✓(2.69)	✓(2.78)
s-s	Q493	E35	✓(2.94)	✓(3.01)	✓(3.46)	✓(2.99)	✓(2.94)	✓(2.78)	✓(2.87)
**SB**	**ACE2**	**ACE2**	**F456**	**I456**	**S456**	**T456**	**D456**	**K456**	**W456**
	K31	E35	✓(3.94)	✓(4.01)	✓(3.93)	✓(3.79)	✓(3.74)	✓(3.72)	✓(3.89)
**HB**	**ACE2**	**ACE2**	**F456**	**I456**	**S456**	**T456**	**D456**	**K456**	**W456**
s-s	D30	H34	✓(3.31)	✓(3.43)	✓(2.95)	✓(3.49)	✓(2.64)	✓(3.36)	✓(2.96)
**HB**	**COV-2**	**COV-2**	**F456**	**I456**	**S456**	**T456**	**D456**	**K456**	**W456**
s-s	Q493	S494	✓(3.24)	✗(p)	✗(p)	✗(p)	✗(p)	✗(p)	✗(p)
**CI**	**COV-2**	**ACE2**	**F456**	**I456**	**S456**	**T456**	**D456**	**K456**	**W456**
p	Y453	H34	✓	✓	✓	✓	✗	✓	✓
vdW/h	X456	T27	✓	✓	✓	✓	✓(p)	✓(HB,2.86)	✓
vdW/h	X456	D30	✓	✗	✗	✓(p)	✗	✗	✓(HB,3.16)
vdW/h	X456	K31	✓	✗	✗	✗	✗	✗	✓
**CI**	**COV-2**	**COV-2**	**F456**	**I456**	**S456**	**T456**	**D456**	**K456**	**W456**
π/c	X456	K417	✓	✗	✗	✗	✗	✗	✓
vdW/h	X456	Y473	✗	✓	✗	✓	✓(HB,2.75)	✓(HB,3.24)	✓

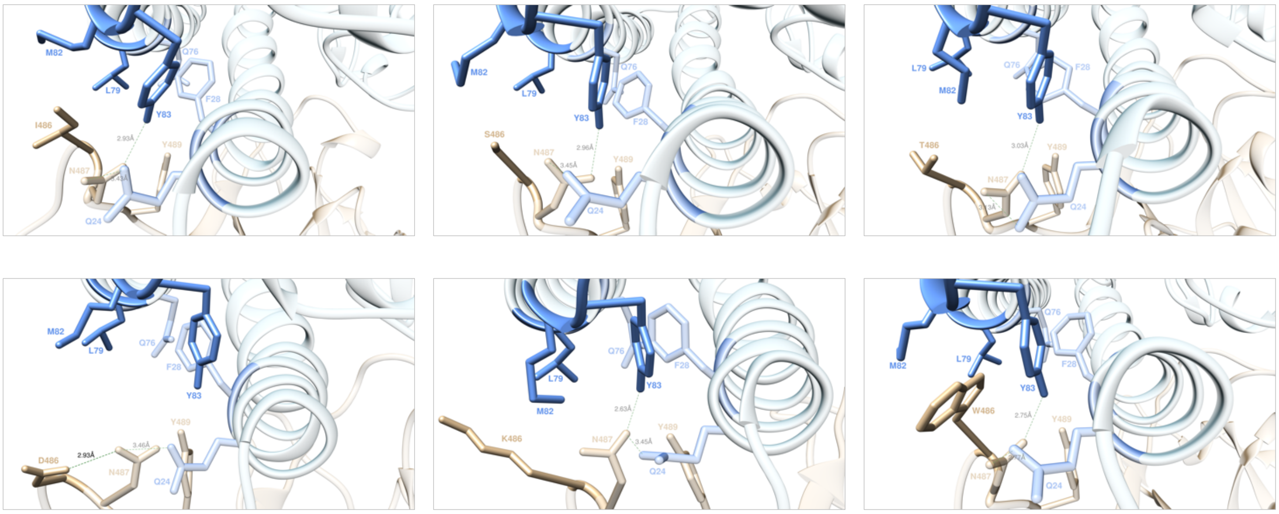

Figure S27. Main interactions involving the S-RBD$_{CoV-2}$ I486 (top left), S486 (top middle), T486 (top right), D486 (bottom left), K486 (bottom middle), and W486 (bottom right) at the interface with ACE2 as obtained from the corresponding equilibrated MD simulations. The wild-type residue F486 is presented and discussed in the main text (Figure 6A). Colors and other explanations as in Figure S19. For further details see Tables S2 and S29.

Table S29. Main intermolecular and intramolecular interactions between the wild-type S-RBD$_{CoV-2}$ residue F486 and all considered mutants at the protein-protein interface detected during MD simulations of ACE2 in complex with the RBD of SARS-CoV-2 (COV-2). Acronyms and other explanations as in Table 19.

**HB**	**COV-2**	**ACE2**	**F486**	**I486**	**S486**	**T486**	**D486**	**K486**	**W486**
s-s	N487	Y83	✓(2.88)	✓(2.93)	✓(2.96)	✓(3.03)	✗(p)	✓(2.63)	✓(2.75)
s-s	N487	Q24	✓(3.03)	✓(3.43)	✓(3.45)	✓(3.13)	✓(3.46)	✓(3.45)	✓(2.77)
**HB**	**COV-2**	**COV-2**	**F486**	**I486**	**S486**	**T486**	**D486**	**K486**	**W486**
s-s	X486	N487	✗	✗	✗	✗	✓(2.93)	✗	✗
**CI**	**COV-2**	**ACE2**	**F486**	**I486**	**S486**	**T486**	**D486**	**K486**	**W486**
p	Y489	Y83	✓	✓	✓	✓	✓	✓	✓
vdW/h	X486	L79	✓	✗	✗	✗	✗	✗	✓
vdW/h	X486	M82	✓	✗	✗	✓	✗	✓	✓
vdW/h	X486	Y83	✓	✗	✗	✗	✗	✗	✓
**CI**	**ACE2**	**ACE2**	**F486**	**I486**	**S486**	**T486**	**D486**	**K486**	**W486**
vdW/h	F28	Y83	✓	✓	✓	✓	✓	✓	✓
vdW/h	F28	Q76	✓	✓	✓	✓	✓	✓	✓
vdW/h	L79	M82	✓	✓	✓	✓	✓	✓	✓

Our MD simulations show that the full network of inter- and intramolecular interactions detected for the wild-type S-RBD$_{CoV-2}$ F486 residue is conserved in the presence of the W486 mutant (bottom right panel in Figure S27 and Table S29). In line with this, the predicted affinity of the S-protein W486 mutant RBD for the human receptor is not significantly different than that of the wild-type counterpart ($\Delta\Delta G_{CoV-2}$(F486W) = +0.18 ± 0.13, Table S2).

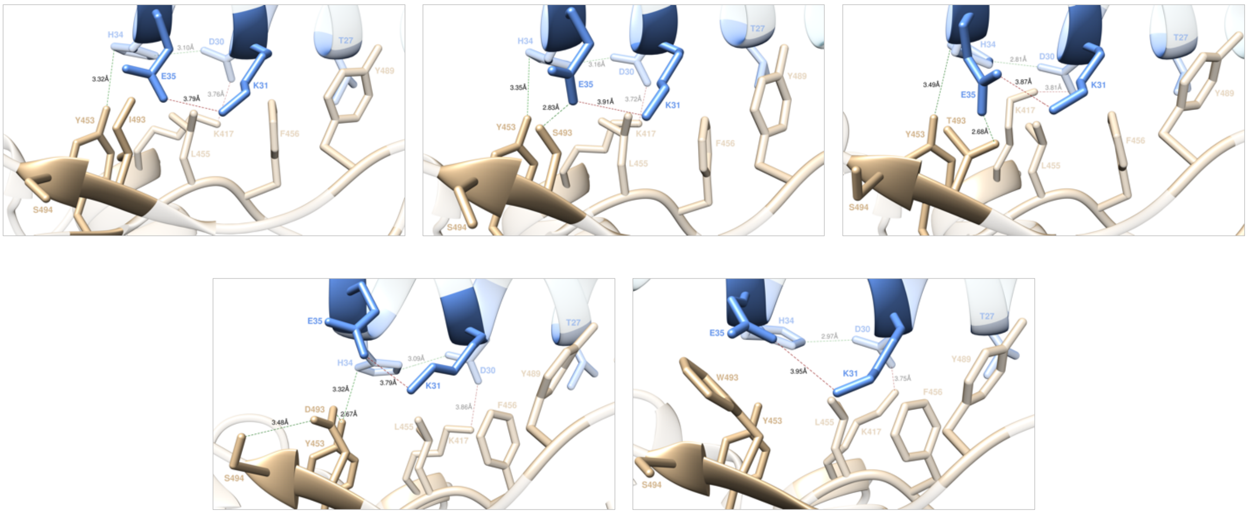

Figure S28. Main interactions involving the S-RBD$_{CoV-2}$ I493 (top left), S493 (top middle), T493 (top right), D486 (bottom left), and W493 (bottom right) at the interface with ACE2 as obtained from the corresponding equilibrated MD simulations. The wild-type residue Q493 and the K493 mutant are presented and discussed in the main text (Figures 4A/8A and Figure 20C). Colors and other explanations as in Figure S19. For further details see Tables S2 and S30.

Table S30. Main intermolecular and intramolecular interactions between the wild-type S-RBD$_{CoV-2}$ residue Q493 and all considered mutants* at the protein-protein interface detected during MD simulations of ACE2 in complex with the RBD of SARS-CoV-2 (COV-2). Acronyms and other explanations as in Table 19. *Mutant K493 is discussed in detail in main text.

SB	COV-2	ACE2	Q493	I493	S493	T493	D493	K493	W493
	K417	D30	✓(3.85)	✓(3.76)	✓(3.72)	✓(3.81)	✓(3.86)	✓(3.82)	✓(3.75)
**HB**	**COV-2**	**ACE2**	**Q493**	**I493**	**S493**	**T493**	**D493**	**K493**	**W493**
s-s	X493	K31	✓(3.04)	✗	✗(p)	✗(p)	✗(p)	✗	✗
s-s	X493	E35	✓(2.94)	✗	✓(2.83)	✓(2.68)	✗	✓(SB,3.77,3.95)	✗
**SB**	**ACE2**	**ACE2**	**Q493**	**I493**	**S493**	**T493**	**D493**	**K493**	**W493**
	K31	E35	✓(3.94)	✓(3.79)	✓(3.91)	✓(3.87)	✓(3.79)	✓(3.80)	✓(3.95)
**HB**	**ACE2**	**ACE2**	**Q493**	**I493**	**S493**	**T493**	**D493**	**K493**	**W493**
s-s	D30	H34	✓(3.31)	✓(3.10)	✓(3.16)	✓(2.81)	✓(3.09)	✓(2.89)	✓(2.97)
**HB**	**COV-2**	**COV-2**	**Q493**	**I493**	**S493**	**T493**	**D493**	**K493**	**W493**
s-s	X493	S494	✓(3.24)	✗	✗	✗	✓(3.48)	✓(3.50)	✗
**CI**	**COV-2**	**ACE2**	**Q493**	**I493**	**S493**	**T493**	**D493**	**K493**	**W493**
p	Y453	H34	✓	✓(HB,3.32)	✓(HB,3.35)	✓(HB,3.49)	✓(HB,3.32)	✓(HB,3.30)	✓
**CI**	**COV-2**	**COV-2**	**Q493**	**I493**	**S493**	**T493**	**D493**	**K493**	**W493**
vdW/h	X493	Y453	✓	✓	✓	✓	✓(HB,2.67)	✓(p)	✗

The analysis of the relevant MD trajectories reveals that, with respect to the wild-type Q493 the I493 mutant is no longer able to form two fundamental HBs across the protein/protein interface with the side chains of K31 and E35, respectively (Figures 4A/8A and S28 (top left panel), and Table S30). Also, I493 no longer engages S494 in the intramolecular HB seen for the wild-type complex (Figures 4A/8A and S28 (top left panel), and Table S30). Accordingly, the predicted affinity of the mutant viral protein for the human receptor is lower than that of the native counterpart ($\Delta\Delta G_{CoV-2}$(Q493I) = -2.77 ± 0.19, Figure 20A in main text and Table S2). For the T493 mutant, the network of interactions stably detected along the corresponding MD trajectory is essentially coincident with that observed for the S493 mutant (compare the top middle and top right panels in Figure S28 and the set of interactions listed in Table S30). In line with this, the corresponding values of $\Delta\Delta G$ are also comparable ($\Delta\Delta G_{CoV-2}$(Q493S) = -0.89 ± 0.12 and $\Delta\Delta G_{CoV-2}$(Q493T) = -0.91

± 0.17, respectively, Figure 20A in main text and Table S2). Therefore, in our case both mutations are seen to mildly interfere in the corresponding protein-protein binding, while the relevant experiments report the same effect for S493 and a neutral effect for T493.[4] Finally, for the W493 variant a neutral effect is reported from experiment[4] whereas our MD simulations predict a negative effect on binding between the mutant S-RBD$_{CoV-2}$ and the human receptor (Figure 20A and Table S2). In our case, the result is supported by the absence of the two, fundamental SBs between the side chains of W493 and ACE2 K31 and E35, respectively, and of the intramolecular HB between the mutant residue and S494 (Figure S28, bottom right panel). Thus, in keeping with the negative effect of replacing the polar, native Q493 with a hydrophobic residue such as isoleucine (see above), the replacement of Q with W at the same position leads to a decrease in binding affinity ($\Delta\Delta G_{CoV-2}$(Q493W) = -2.88 ± 0.18, Figure 20A and Table S2).

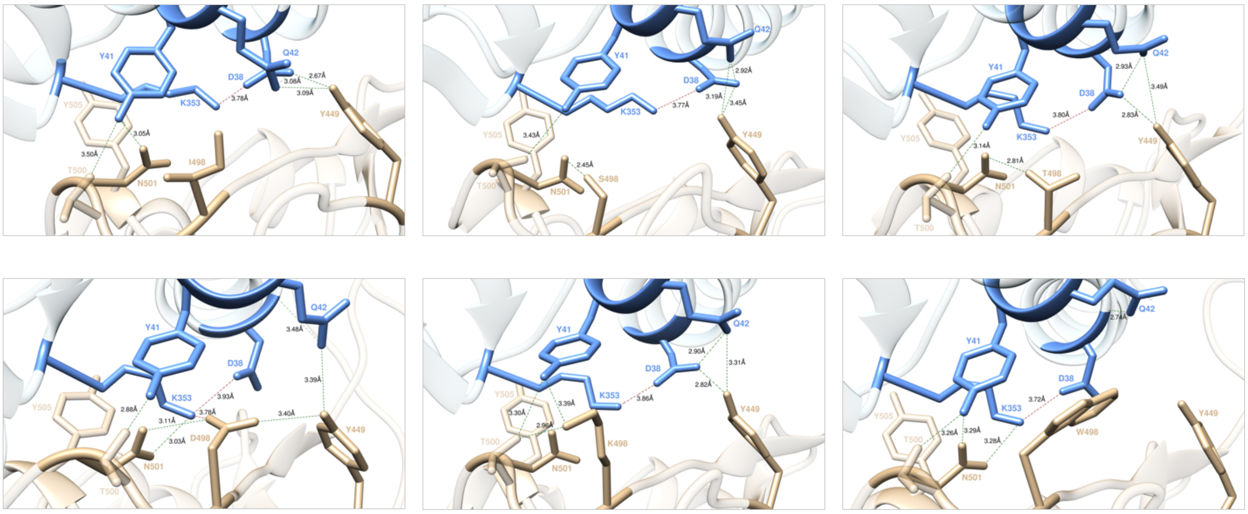

Figure S29. Main interactions involving the S-RBD$_{CoV-2}$ I498 (top left), S498 (top middle), T498 (top right), D498 (bottom left), K498 (bottom middle) and W498 (bottom right) at the interface with ACE2 as obtained from the corresponding equilibrated MD simulations. The wild-type residue Q498 is presented and discussed in the main text (Figure 12A). Colors and other explanations as in Figure S19. For further details see Tables S2 and S31.

Table S31. Main intermolecular and intramolecular interactions between the wild-type S-RBD$_{CoV-2}$ residue Q498 and all considered mutants at the protein-protein interface detected during MD simulations of ACE2 in complex with the RBD of SARS-CoV-2 (COV-2). Acronyms and other explanations as in Table 19.

**HB**	**COV-2**	**ACE2**	**Q498**	**I498**	**S498**	**T498**	**D498**	**K498**	**W498**
s-s	X498	K353	✓(2.87)	✗	✗	✗	✓(SB,3.78)	✗	✗
s-s	X498	D38	✓(2.92)	✗	✗	✗	✗(p)	✗	✗
s-s	X498	T500	✗	✗	✗	✗	✗	✓(2.96)	✗
s-s	Y449	D38	✓(2.92)	✓(2.67)	✓(3.45)	✓(2.83)	✗	✓(2.82)	✗
s-s	Y449	Q42	✓(3.03)	✓(3.09)	✓(3.19)	✓(3.49)	✓(3.39)	✓(3.31)	✗
s-s	T500	Y41	✓(3.08)	✓(3.50)	✓(3.43)	✓(3.14)	✓(2.88)	✓(3.30)	✓(3.26)
s-s	N501	Y41	✓(3.23)	✓(3.05)	✗(p)	✗(p)	✗(p)	✗(p)	✓(3.29)
**SB**	**ACE2**	**ACE2**	**Q498**	**I498**	**S498**	**T498**	**D498**	**K498**	**W498**
	K353	D38	✓(3.66)	✓(3.78)	✓(3.77)	✓(3.80)	✓(3.93)	✓(3.86)	✓(3.72)
**HB**	**ACE2**	**ACE2**	**Q498**	**I498**	**S498**	**T498**	**D498**	**K498**	**W498**
b-s	D38	Q42	✓(3.04)	✓(s-s,3.08)	✓(s-s,3.06)	✓(s-s,2.93)	✓(3.48)	✓(s-s,2.90)	✓(2.74)
**HB**	**COV-2**	**COV-2**	**Q498**	**I498**	**S498**	**T498**	**D498**	**K498**	**W498**
s-s	X498	N501	✓(3.02)	✗	✓(2.45)	✓(2.81)	✓(3.11)	✗(p)	✗
s-s	X498	Y449	✓(3.04)	✗	✗	✗	✓(3.04)	✗	✗
**CI**	**COV-2**	**ACE2**	**Q498**	**I498**	**S498**	**T498**	**D498**	**K498**	**W498**
p	N501	K353	✓	✓	✓	✓	✓(HB,3.03)	✓	✓(HB,3.28)
vdW/h	X498	Y41	✓	✓	✓(p)	✓(p)	✓(p)	✓(HB,3.39)	✓
vdW/h	X498	Q42	✓	✗	✗	✗	✗	✗	✗

In the case of the W498 mutant, the present simulations show that all three main intermolecular HBs in which the wild-type residue is involved (*i.e.*, Q498-K353, Q498-D38, and Q498-T500) cannot longer be detected in the trajectory of the mutant complex. Also, the stabilizing internal HB between Q498 and the side chain N501 is no longer formed in the presence of the W498 mutation (Figure S29, top panel and Table S31). These evidences, along with the missing CI with

ACE2 Q42, concur to lower the predicted affinity of the W498 mutant S-RBD$_{CoV-2}$ for the human receptor ($\Delta\Delta G_{CoV-2}$(Q498W) = 4.18 ± 0.11 kcal/mol, Figure 20B in main text and Table S2).

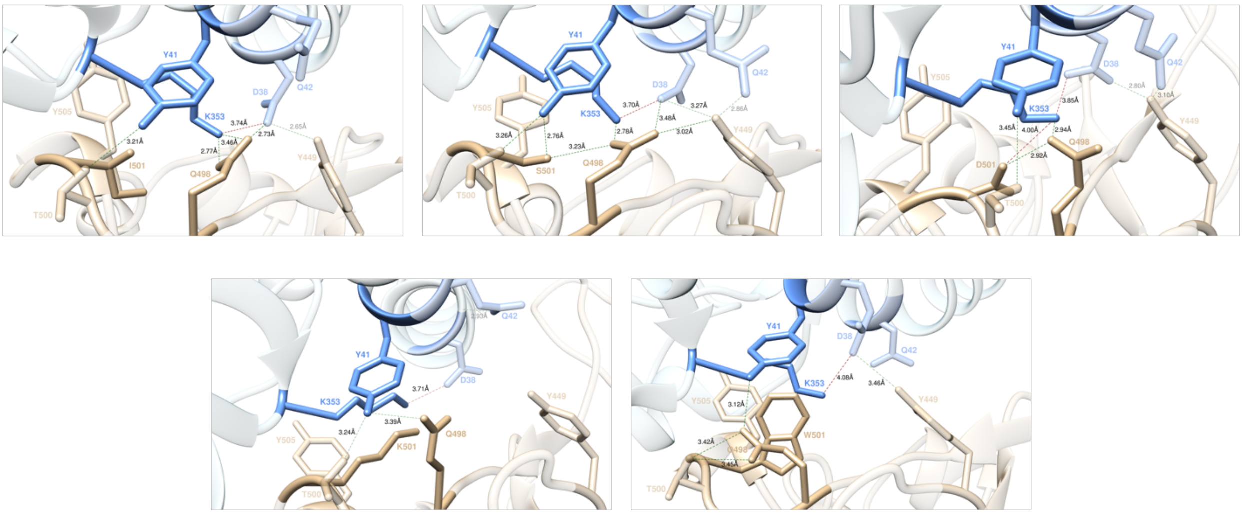

Figure S30. Main interactions involving the S-RBD$_{CoV-2}$ I501 (top left), S501 (top middle), D501 (top right), K501 (bottom left), and W501 (bottom right) at the interface with ACE2 as obtained from the corresponding equilibrated MD simulations. The wild-type residue N501 and the T501 mutant are presented and discussed in the main text (Figures 12A and 21D). Colors and other explanations as in Figure S19. For further details see Tables S2 and S32.

Table S32. Main intermolecular and intramolecular interactions between the wild-type S-RBD$_{CoV-2}$ residue N501 and all considered mutants* at the protein-protein interface detected during MD simulations of ACE2 in complex with the RBD of SARS-CoV-2 (COV-2). Acronyms and other explanations as in Table 19. *Mutant T501 is discussed in detail in main text.

**HB**	**COV-2**	**ACE2**	**N501**	**I501**	**S501**	**T501**	**D501**	**K501**	**W501**
s-s	Q498	K353	✓(2.87)	✓(2.77,3.46)	✓(2.78)	✓(2.76,3.06)	✓(2.94)	✗	✗
s-s	Q498	D38	✓(2.92)	✓(2.73)	✓(3.48)	✓(3.38)	✗	✗	✗
s-s	Y449	D38	✓(2.92)	✓(2.65)	✓(3.23)	✓(3.29)	✓(2.80)	✗(p)	✓(3.46)
s-s	Y449	Q42	✓(3.03)	✗(p)	✓(3.27)	✓(2.78)	✓(3.10)	✗	✗(p)
s-s	X501	Y41	✓(3.23)	✗	✓(2.76)	✓(3.36)	✗(p)	✗	✗(π/π)
s-s	T500	Y41	✓(3.08)	✓(3.21)	✓(3.26)	✓(2.84)	✓(3.45)	✓(3.24)	✗
**SB**	**ACE2**	**ACE2**	**N501**	**I501**	**S501**	**T501**	**D501**	**K501**	**W501**
	K353	D38	✓(3.66)	✓(3.74)	✓(3.70)	✓(3.69)	✓(3.85)	✓(3.71)	✓(4.08)
**HB**	**ACE2**	**ACE2**	**N501**	**I501**	**S501**	**T501**	**D501**	**K501**	**W501**
b-s	D38	Q42	✓(3.04)	✗	✗(p)	✗(p)	✗	✓(2.93)	✗
**HB**	**COV-2**	**COV-2**	**N501**	**I501**	**S501**	**T501**	**D501**	**K501**	**W501**
s-s	Q498	X501	✓(3.02)	✗	✓(3.23)	✓(3.48)	✓(2.92)	✗(p)	✗
s-s	Y449	Q498	✓(3.04)	✗(p)	✓(3.02)	✓(3.37)	✗	✗(p)	✗
s-s	T500	Q498	✗	✗	✗	✗	✗	✗	✓(3.42,3.45)
**CI**	**COV-2**	**ACE2**	**N501**	**I501**	**S501**	**T501**	**D501**	**K501**	**W501**
p	X501	K353	✓	✗	✓	✓	✓(SB,4.00)	✗	✓(π/c)
p	Q498	Q42	✗	✗	✗	✗	✗	✗	✗
vdW/h	Q498	Y41	✓	✗	✓	✓	✓	✓(HB,3.39)	✓(HB,3.12)

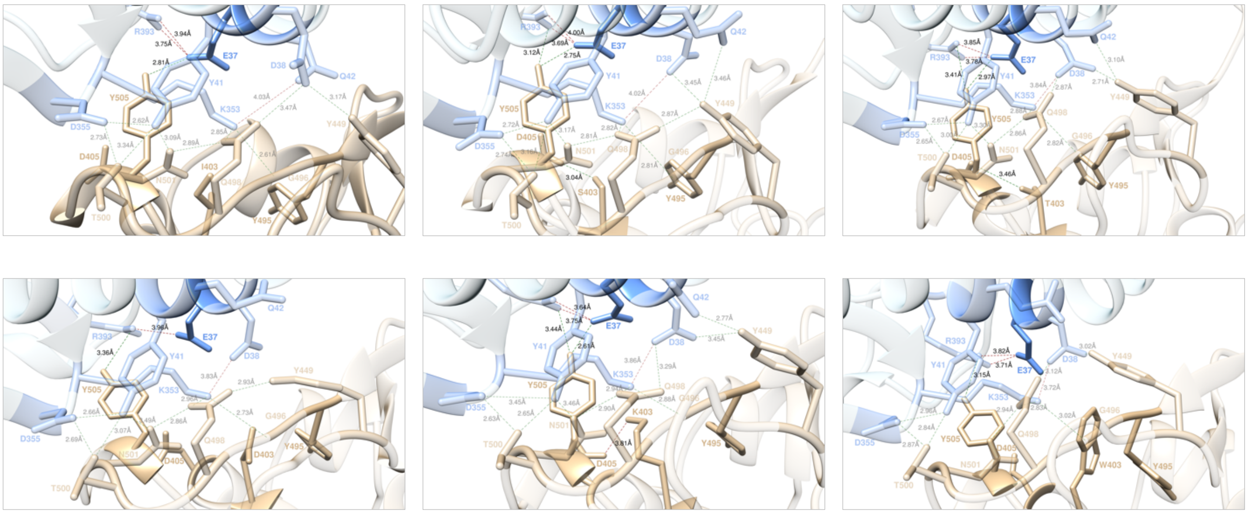

Figure S31. Main interactions involving the S-RBD$_{CoV-2}$ I403 (top left), S403 (top middle), T403 (top right), D403 (bottom left), K403 (bottom middle), and W403 (bottom right) at the interface with ACE2 as obtained from the corresponding equilibrated MD simulations. The wild-type residue R403 is presented and discussed in the main text (Figures 12 A and 14A). Colors and other explanations as in Figure S19. For further details see Tables S2 and S33.

Table S33. Main intermolecular and intramolecular interactions between the wild-type S-RBD$_{CoV-2}$ residue R403 and all considered mutants at the protein-protein interface detected during MD simulations of ACE2 in complex with the RBD of SARS-CoV-2 (COV-2). Acronyms and other explanations as in Table 19.

SB	COV-2	ACE2	R403	I403	S403	T403	D403	K403	W403
	X403	E37	✓(3.62)	✗	✗	✗	✗	✗(p)	✗
**HB**	**COV-2**	**ACE2**	**R403**	**I403**	**S403**	**T403**	**D403**	**K403**	**W403**
s-s	T500	Y41	✓(3.08)	✓(3.34)	✓(3.16)	✓(3.00)	✓(3.07)	✓(2.65)	✓(2.84)
s-s	T500	D355	✓(2.77)	✓(2.73)	✓(2.74)	✓(2.65)	✓(2.69)	✓(2.63)	✓(2.87)
s-s	N501	Y41	✓(3.23)	✓(3.09)	✓(3.17)	✓(3.30)	✓(3.49)	✓(3.46)	✗(p)
s-s	Y505	E37	✓(3.15)	✓(2.81)	✓(2.75)	✓(2.97)	✗	✓(2.61)	✗
s-s	Y505	R393	✗(p)	✗(p)	✓(3.12)	✓(3.41)	✓(3.36)	✓(3.44)	✓(3.15)
s-s	Q498	K353	✓(2.87)	✓(2.85)	✓(2.82)	✓(2.88)	✓(2.96)	✓(2.94)	✓(2.83)
s-s	Q498	D38	✓(2.92)	✓(3.47)	✗(p)	✓(2.87)	✗	✓(3.29)	✓(3.12)
s-s	Y449	D38	✓(2.92)	✓(3.17)	✓(3.45)	✓(2.71)	✗	✓(3.45)	✓(3.02)
s-s	Y449	Q42	✓(3.03)	✗	✓(3.46)	✓(3.10)	✗	✓(2.77)	✗
s-s	G496	K353	✓(2.95)	✓(2.61)	✓(2.81)	✓(2.82)	✓(2.73)	✓(2.88)	✓(3.02)
**SB**	**ACE2**	**ACE2**	**R403**	**I403**	**S403**	**T403**	**D403**	**K403**	**W403**
	E37	R393	✓(3.69,3.93)	✓(3.75,3.94)	✓(3.69,4.00)	✓(3.78,3.85)	✓(3.96)	✓(3.64,3.75)	✓(3.71,3.82)
	K353	D38	✓(3.66)	✓(4.03)	✓(4.02)	✓(3.84)	✓(3.83)	✓(3.86)	✓(3.72)
**SB**	**COV-2**	**COV-2**	**R403**	**I403**	**S403**	**T403**	**D403**	**K403**	**W403**
	X403	D405	✓(3.95)	✗	✓(HB,3.04)	✓(HB,3.46)	✗	✓(3.81)	✗
**HB**	**ACE2**	**ACE2**	**R403**	**I403**	**S403**	**T403**	**D403**	**K403**	**W403**
s-s	Y41	D355	✓(2.78)	✓(2.62)	✓(2.72)	✓(2.67)	✓(2.73)	✓(3.45)	✓(2.96)
**HB**	**COV-2**	**COV-2**	**R403**	**I403**	**S403**	**T403**	**D403**	**K403**	**W403**
s-s	Q498	Y449	✓(3.04)	✗	✓(2.87)	✗	✓(2.93)	✗	✗

s-s	N501	Q498	✓(3.02)	✓(2.89)	✓(2.81)	✓(2.86)	✓(2.86)	✓(2.90)	✓(2.94)
**CI**	**COV-2**	**ACE2**	**R403**	**I403**	**S403**	**T403**	**D403**	**K403**	**W403**
π/c	X403	Y505	✓	✗	✗	✗	✗	✗	✗
vdW/h	X403	Y495	✓	✓	✗	✓	✓	✓	✓

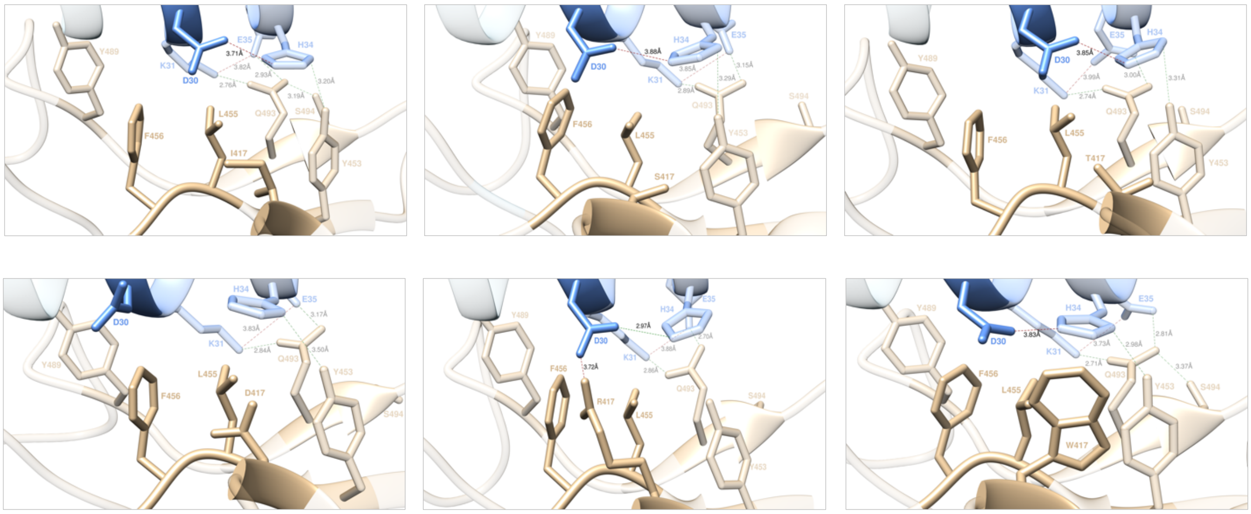

Figure S32. Main interactions involving the S-RBD$_{CoV-2}$ I417 (top left), S417 (top middle), T417 (top right), D417 (bottom left), R417 (bottom middle), and W417 (bottom right) at the interface with ACE2 as obtained from the corresponding equilibrated MD simulations. The wild-type residue K417 is presented and discussed in the main text (Figures 4A and 8A). Colors and other explanations as in Figure S19. For further details see Tables S2 and S34.

Table S34. Main intermolecular and intramolecular interactions between the wild-type S-RBD$_{CoV-2}$ residue K417 and all considered mutants at the protein-protein interface detected during MD simulations of ACE2 in complex with the RBD of SARS-CoV-2 (COV-2). Acronyms and other explanations as in Table 19.

**SB**	**COV-2**	**ACE2**	**K417**	**I417**	**S417**	**T417**	**D417**	**R417**	**W417**
	X417	D30	✓(3.85)	✗	✗	✗	✗	✓(3.72)	✗
**HB**	**COV-2**	**ACE2**	**K417**	**I417**	**S417**	**T417**	**D417**	**R417**	**W417**
s-s	Q493	K31	✓(3.04)	✓(2.76)	✓(2.89)	✓(2.74)	✓(2.84)	✓(2.86)	✓(2.71)
s-s	Q493	E35	✓(2.94)	✓(2.93)	✓(3.15)	✓(3.00)	✓(3.17)	✓(2.70)	✓(2.81)
**SB**	**ACE2**	**ACE2**	**K417**	**I417**	**S417**	**T417**	**D417**	**R417**	**W417**
	K31	E35	✓(3.94)	✓(3.82)	✓(3.85)	✓(3.99)	✓(3.83)	✓(3.88)	✓(3.73)
**HB**	**ACE2**	**ACE2**	**K417**	**I417**	**S417**	**T417**	**D417**	**R417**	**W417**
s-s	D30	H34	✓(3.31)	✓(SB,3.71)	✓(SB,3.88)	✓(SB,3.85)	✗	✓(2.97)	✓(SB,3.83)
**HB**	**COV-2**	**COV-2**	**K417**	**I417**	**S417**	**T417**	**D417**	**R417**	**W417**
s-s	Q493	S494	✓(3.24)	✓(3.19)	✗(p)	✗(p)	✗	✗	✓(3.37)
**CI**	**COV-2**	**ACE2**	**K417**	**I417**	**S417**	**T417**	**D417**	**R417**	**W417**
p	Y453	H34	✓	✓(HB,3.20)	✓(HB,3.29)	✓(HB,3.31)	✓(HB,3.50)	✓	✓(HB,2.98)
**CI**	**COV-2**	**COV-2**	**K417**	**I417**	**S417**	**T417**	**D417**	**R417**	**W417**
π/c	X417	F456	✓	✗	✗	✗	✗	✗	✗
vdW/h	X417	L455	✓	✓	✗	✗	✓	✓	✓

References

1. Shang, J.; Ye, G.; Shi, K.; Wan, Y.; Luo, C.; Aihara, H.; Geng, Q.; Auerbach, A.; Li, F., Structural Basis of Receptor Recognition by SARS-CoV-2. *Nature* **2020,** *581*, 221-224.
2. Lan, J.; Ge, J.; Yu, J.; Shan, S.; Zhou, H.; Fan, S.; Zhang, Q.; Shi, X.; Wang, Q.; Zhang, L.; Wang, X., Structure of the SARS-CoV-2 Spike Receptor-Binding Domain Bound to the ACE2 Receptor. *Nature* **2020,** *581*, 215-220.
3. Laurini, E.; Marson, D.; Aulic, S.; Fermeglia, M.; Pricl, S., Computational Alanine Scanning and Structural Analysis of the SARS-CoV-2 Spike Protein/Angiotensin-Converting Enzyme 2 Complex. *ACS Nano* **2020,** *14*, 11821-11830.
4. Starr, T. N.; Greaney, A. J.; Hilton, S. K.; Ellis, D.; Crawford, K. H. D.; Dingens, A. S.; Navarro, M. J.; Bowen, J. E.; Tortorici, M. A.; Walls, A. C.; King, N. P.; Veesler, D.; Bloom, J. D., Deep Mutational Scanning of SARS-CoV-2 Receptor Binding Domain Reveals Constraints on Folding and ACE2 Binding. *Cell* **2020,** *182*, 1295-1310.
