# Supplementary material for: Computational Mutagenesis at the SARS-CoV-2 Spike Protein/Angiotensin-Converting Enzyme 2 Binding Interface: Comparison with Experimental Evidence
Source: ACS Nano. 2021 Mar 18;15(4):6929–48. doi: 10.1021/acsnano.0c10833 (PMC8009103; doi:10.1021/acsnano.0c10833)
Supplement: Supplementary file 3 — nn0c10833_si_003.pdf [file nn0c10833_si_003.pdf]

Supporting info – part 3 for the paper:

# Computational Mutagenesis at the SARS-CoV-2 Spike Protein/Angiotensin-Converting Enzyme 2 Binding Interface: Comparison with Experimental Evidences

Erik Laurini[1,‡], Domenico Marson[1,‡], Suzana Aulic[1], Alice Fermeglia[1], Sabrina Pricl[1,2*]

[1]Molecular Biology and Nanotechnology Laboratory (MolBNL@UniTS), DEA, University of Trieste, 34127 Trieste, Italy
[2]Department of General Biophysics, Faculty of Biology and Environmental Protection, University of Lodz, 90-136 Lodz, Poland

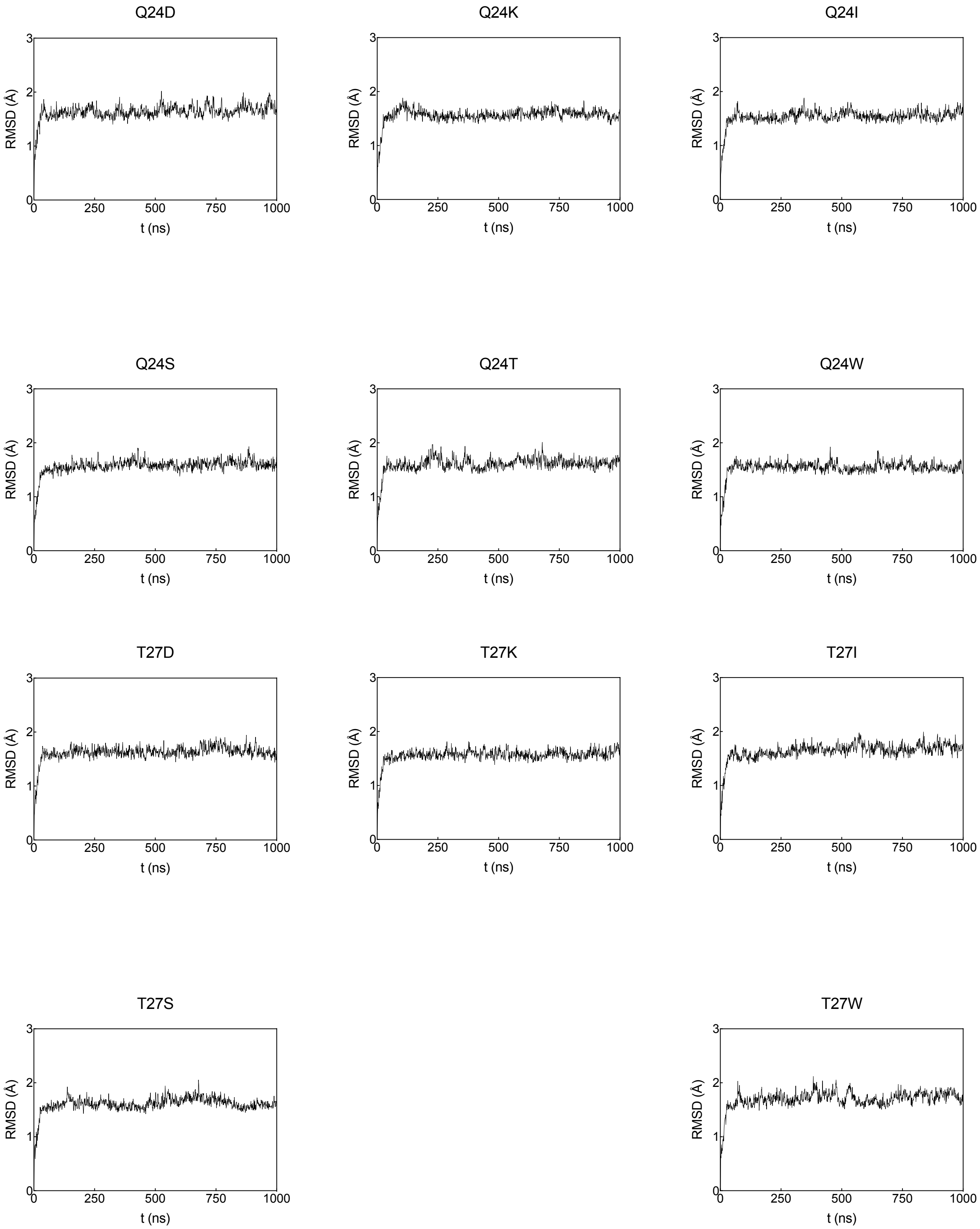
Q24D
Q24K
Q24I
Q24S
Q24T
Q24W
T27D
T27K
T27I
T27S
T27W
RMSD (Å)
t (ns)
0
1
2
3
0
250
500
750
1000

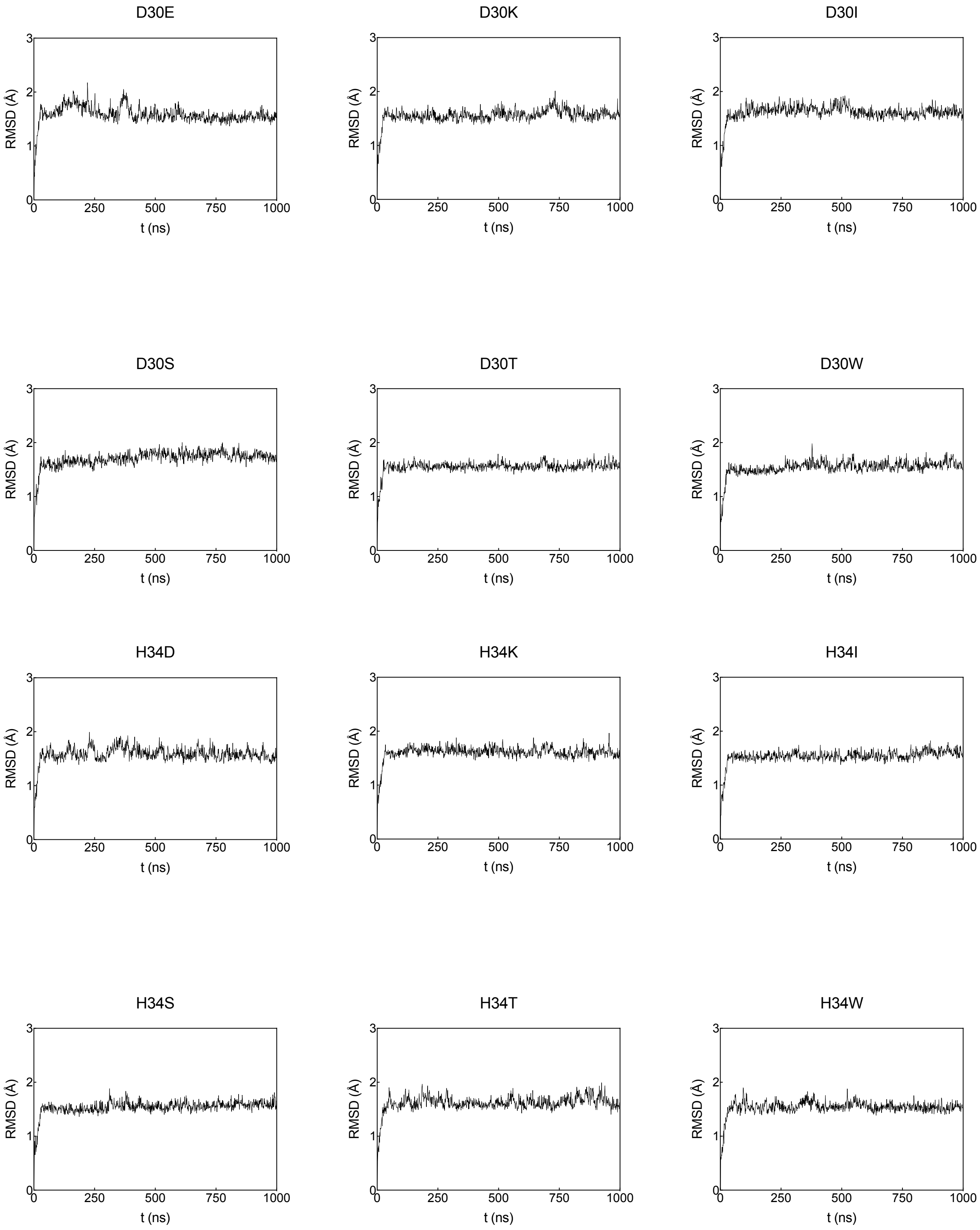

D30E
D30K
D30I
D30S
D30T
D30W
H34D
H34K
H34I
H34S
H34T
H34W
RMSD (Å)
0
1
2
3
t (ns)
0
250
500
750
1000

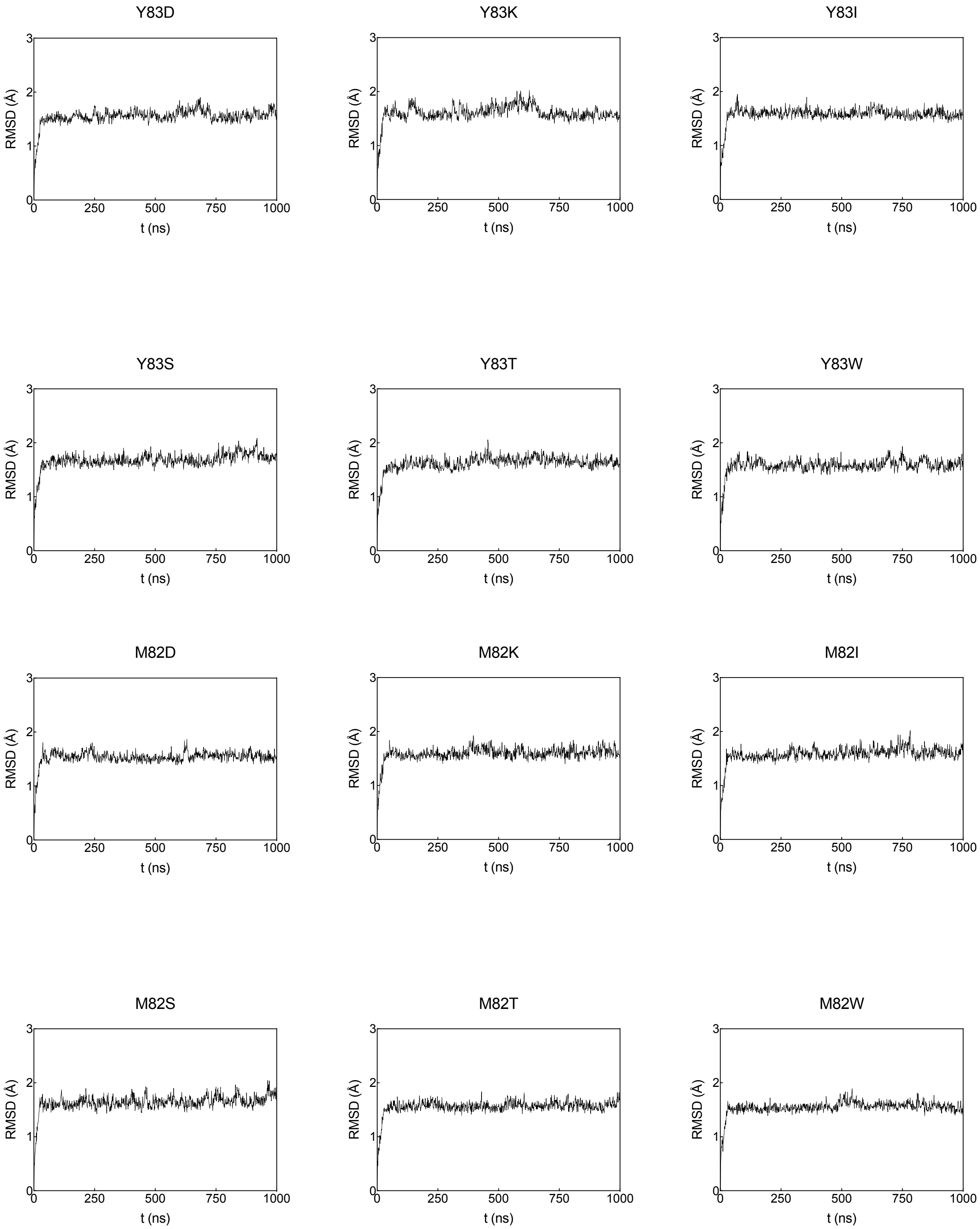
Y83D
Y83K
Y83I
Y83S
Y83T
Y83W
M82D
M82K
M82I
M82S
M82T
M82W
RMSD (Å)
t (ns)

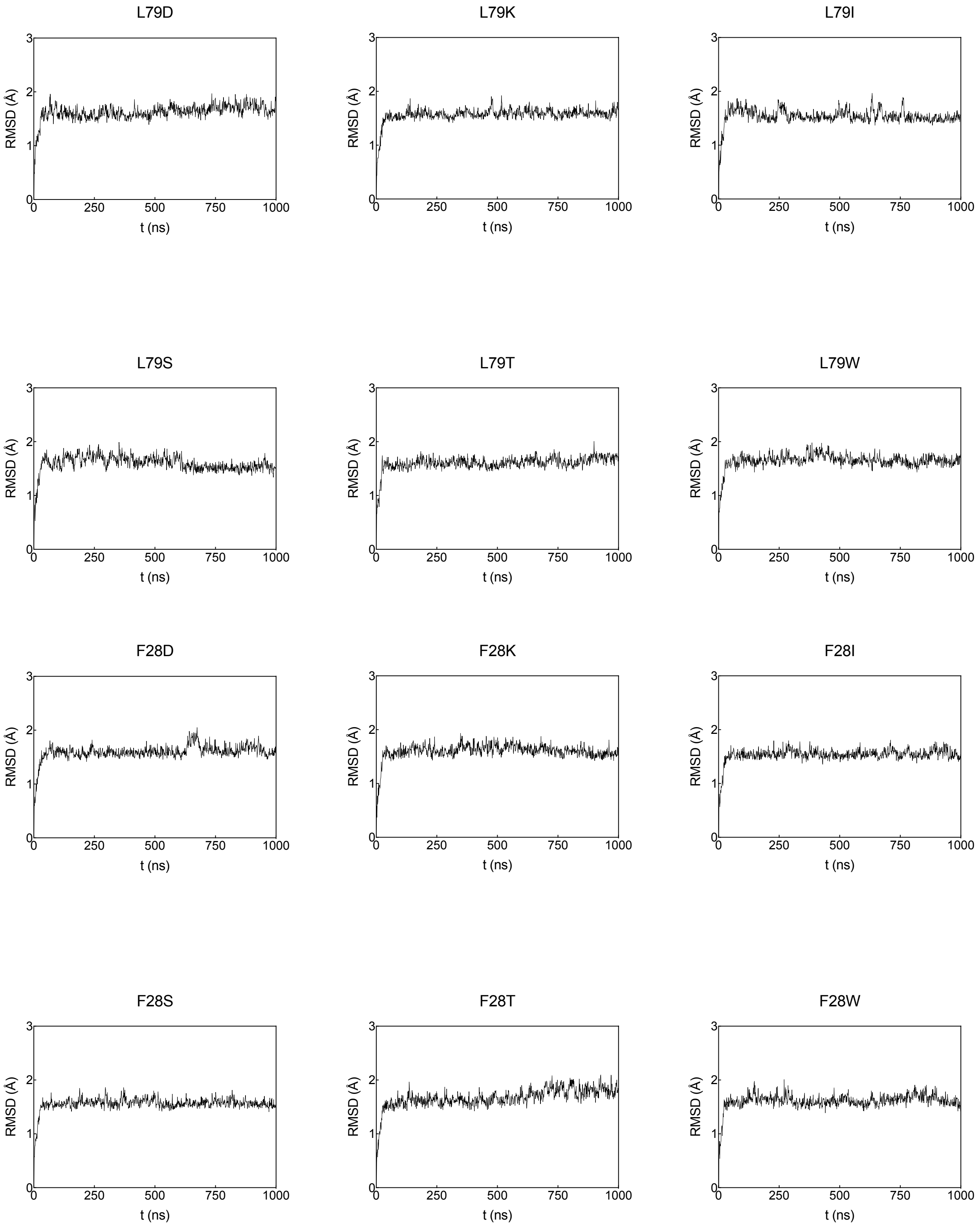
L79D
L79K
L79I
L79S
L79T
L79W
F28D
F28K
F28I
F28S
F28T
F28W
RMSD (Å)
t (ns)
0
1
2
3
250
500
750
1000

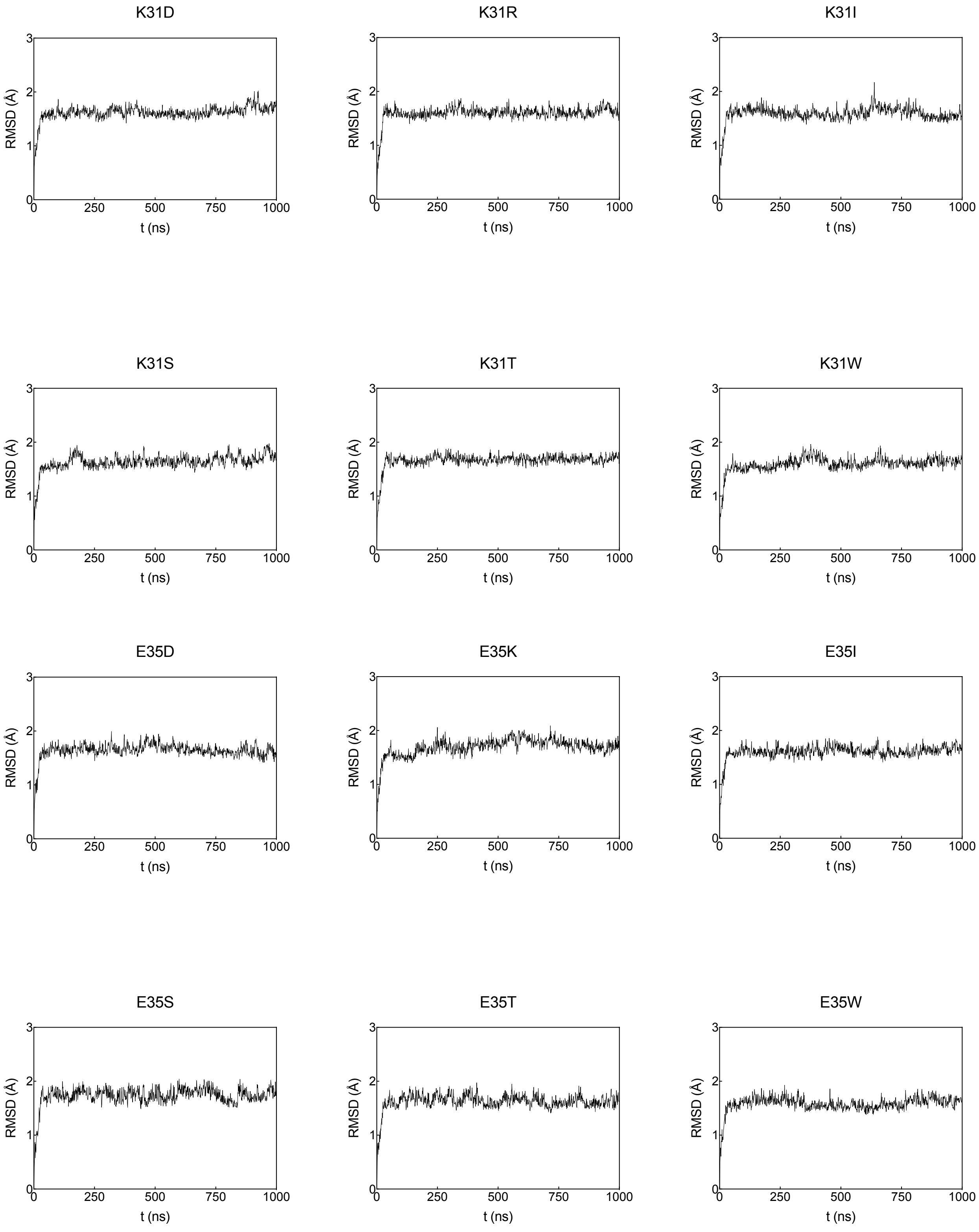
K31D
K31R
K31I
K31S
K31T
K31W
E35D
E35K
E35I
E35S
E35T
E35W
RMSD (Å)
t (ns)
0
1
2
3
250
500
750
1000

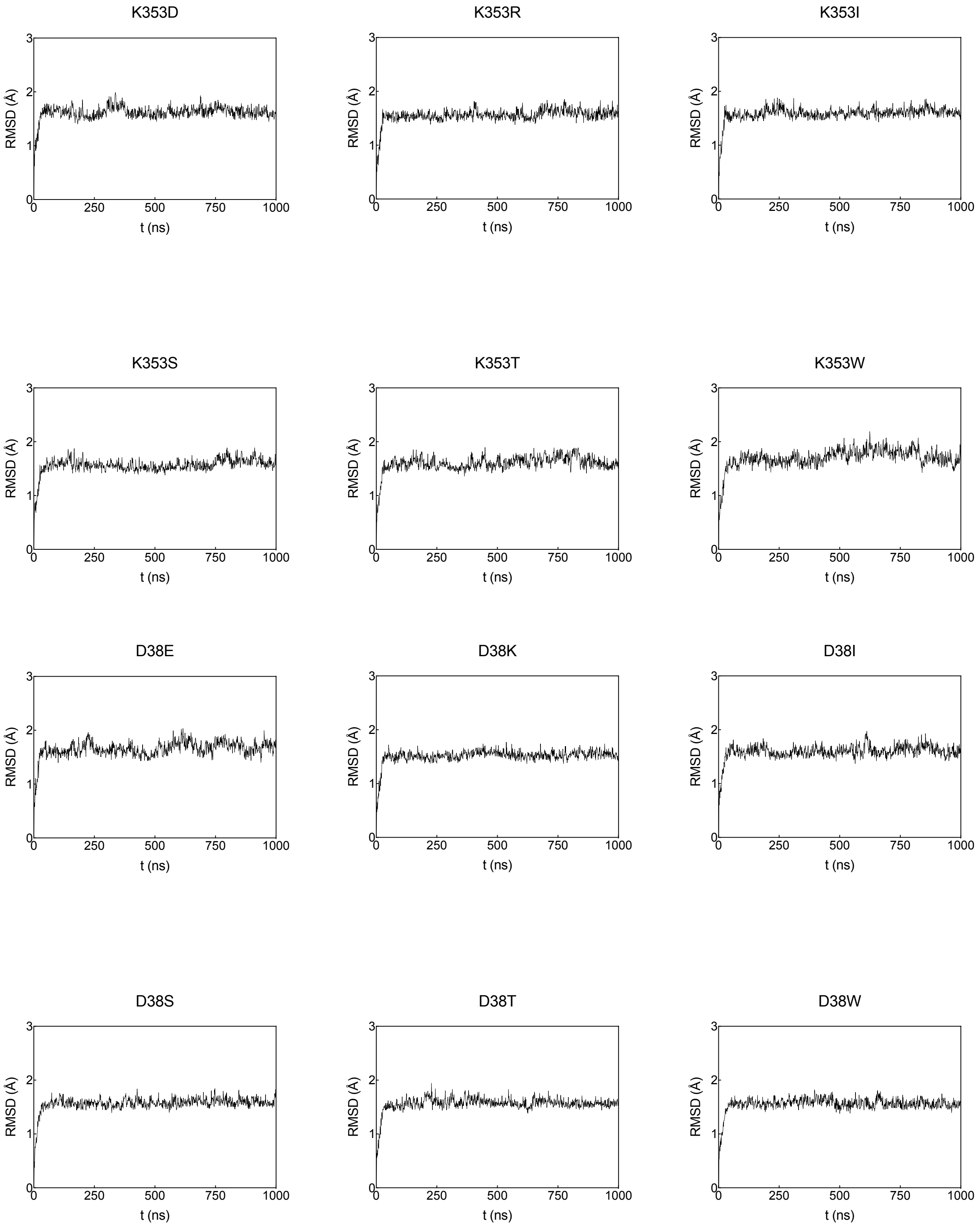
K353D
K353R
K353I
K353S
K353T
K353W
D38E
D38K
D38I
D38S
D38T
D38W
RMSD (Å)
t (ns)
0
1
2
3
0
250
500
750
1000

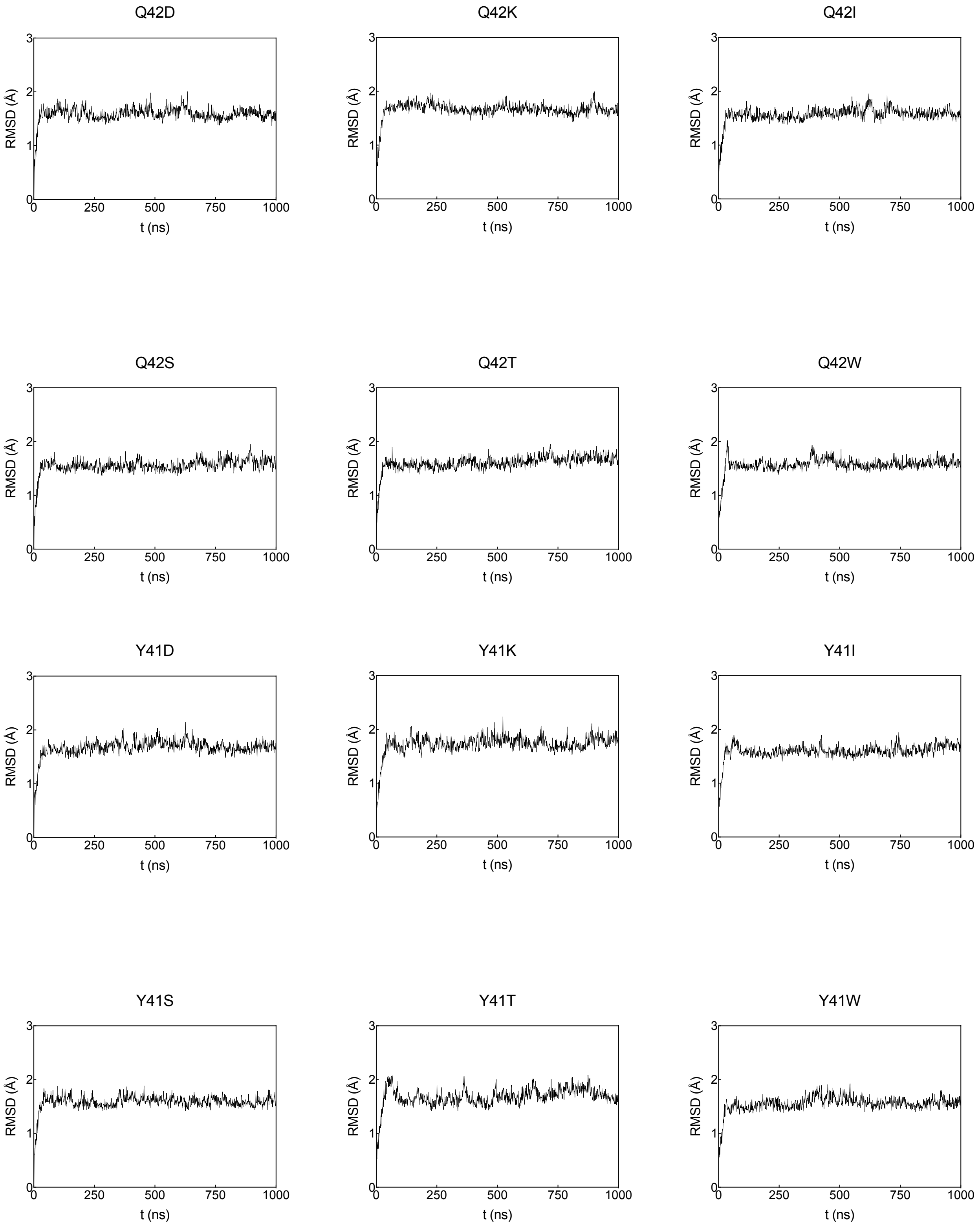
Q42D
Q42K
Q42I
Q42S
Q42T
Q42W
Y41D
Y41K
Y41I
Y41S
Y41T
Y41W
RMSD (Å)
t (ns)
0
1
2
3
250
500
750
1000

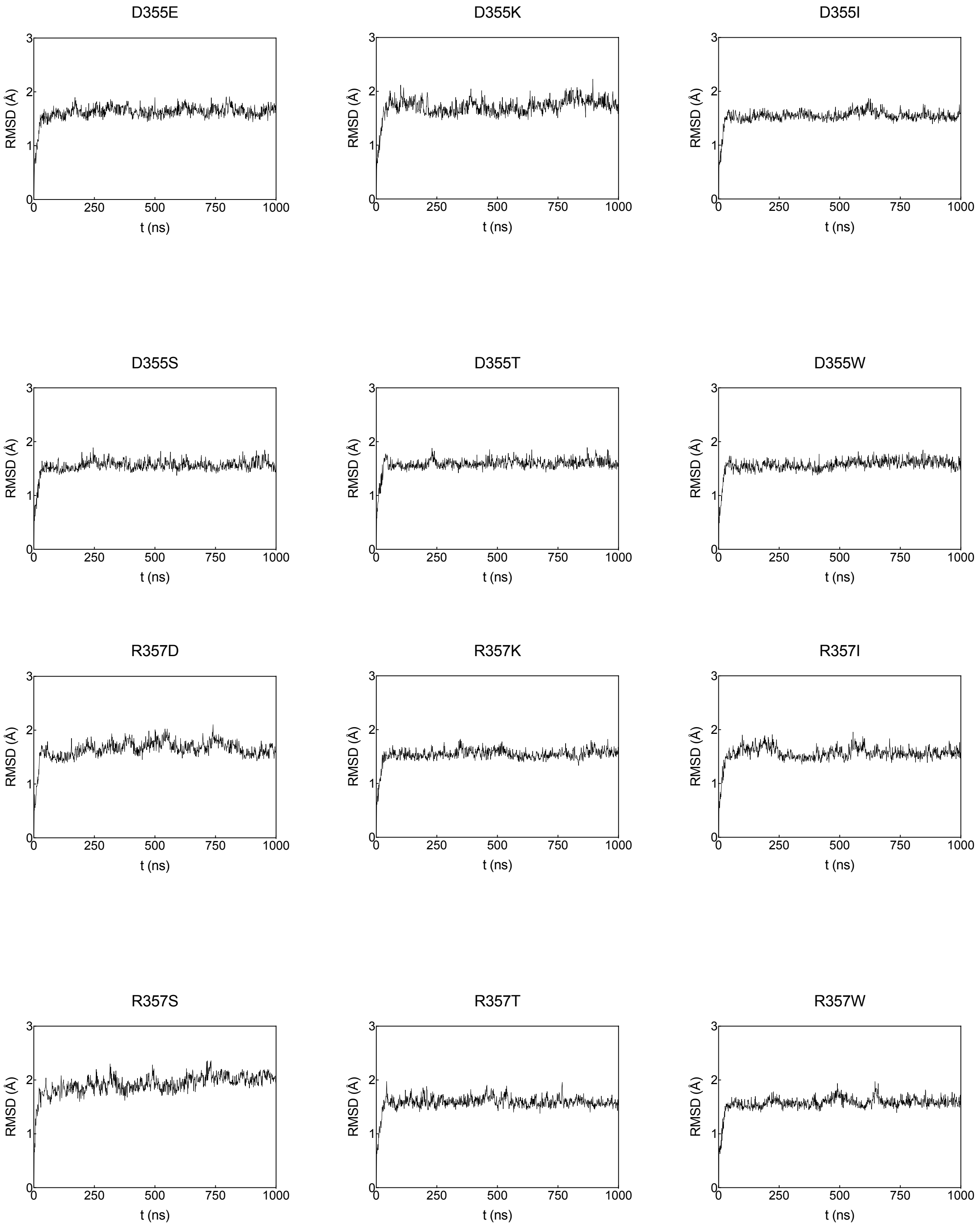
D355E
D355K
D355I
D355S
D355T
D355W
R357D
R357K
R357I
R357S
R357T
R357W
RMSD (Å)
t (ns)
0
1
2
3
250
500
750
1000

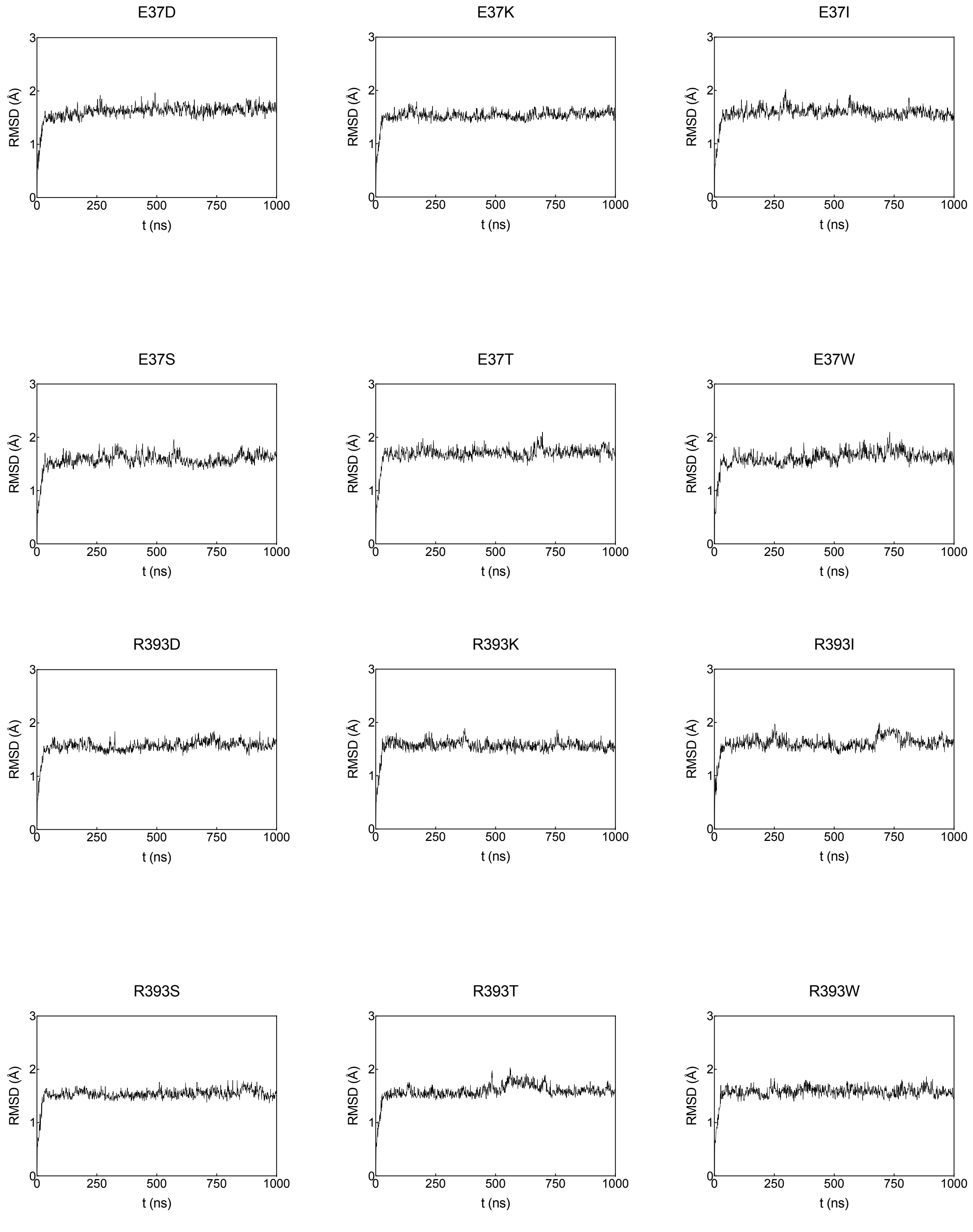


**Figure S33.** Root-mean-square deviation (RMSD) of ACE2/ S-RBD$_{CoV\text{-}2}$ protein complex backbone atoms as a function of MD simulation time for all ACE2 mutants.

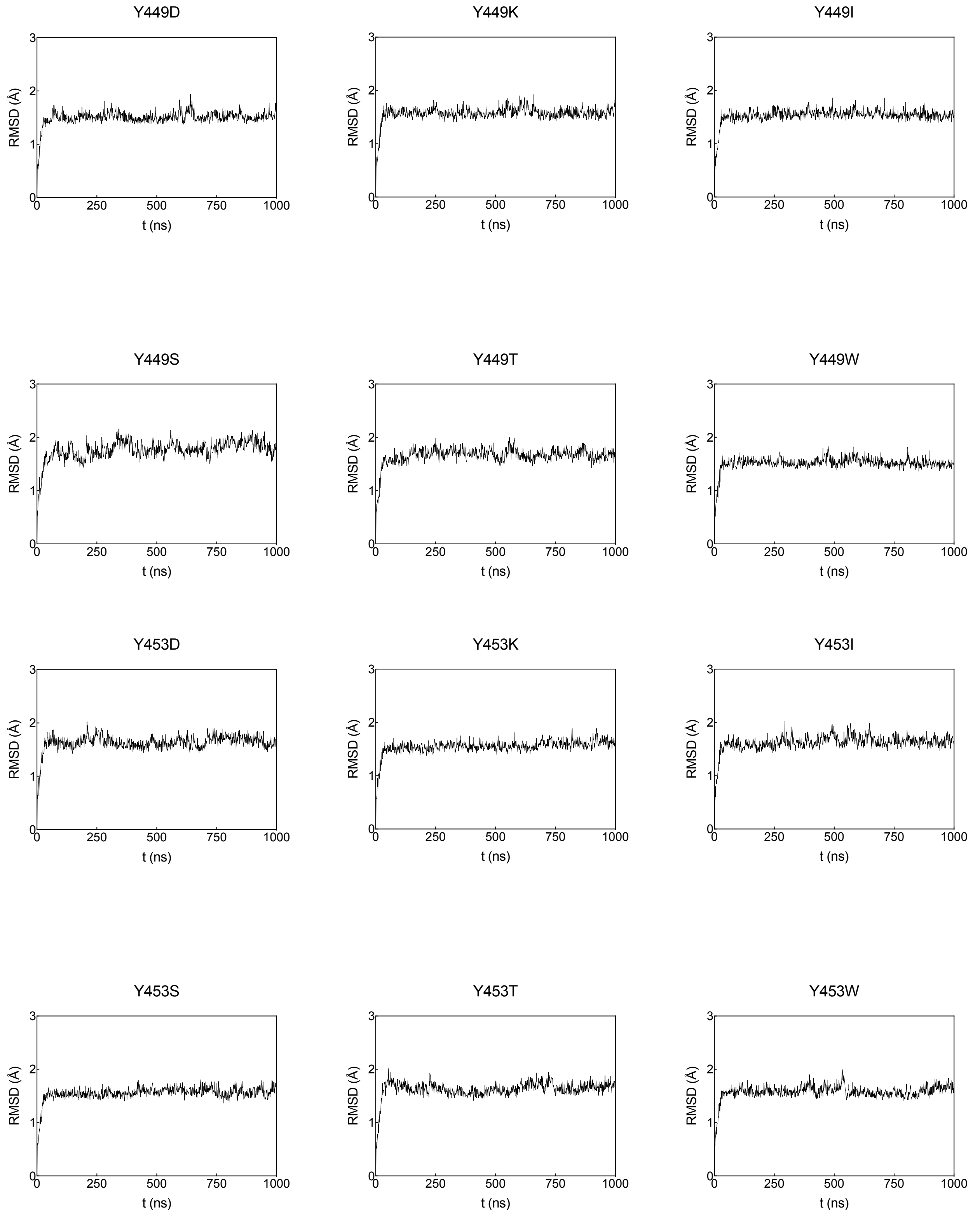
Y449D
Y449K
Y449I
Y449S
Y449T
Y449W
Y453D
Y453K
Y453I
Y453S
Y453T
Y453W
RMSD (Å)
t (ns)
0
1
2
3
250
500
750
1000

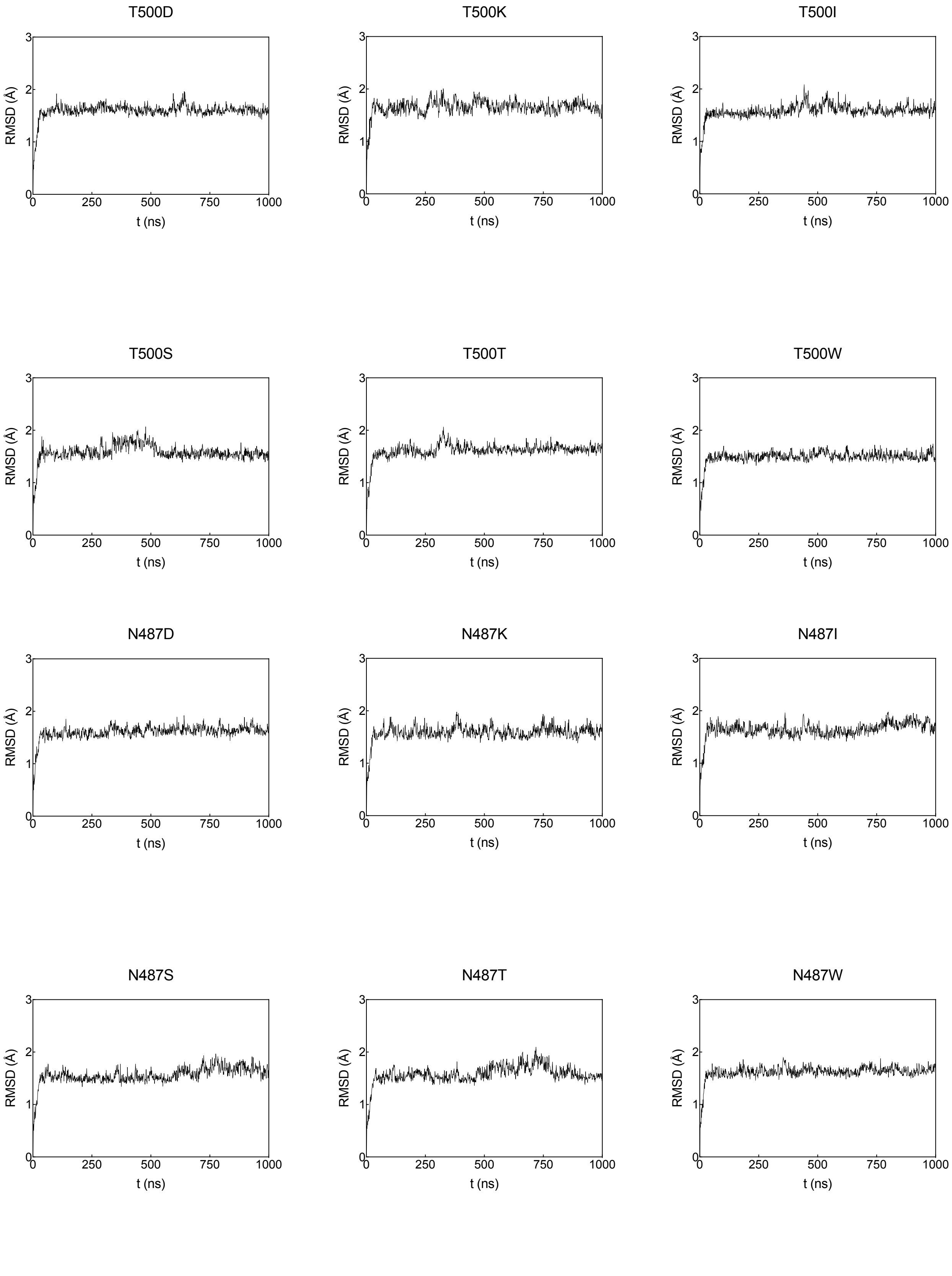
T500D
T500K
T500I
T500S
T500T
T500W
N487D
N487K
N487I
N487S
N487T
N487W
RMSD (Å)
t (ns)
0
1
2
3
0
250
500
750
1000

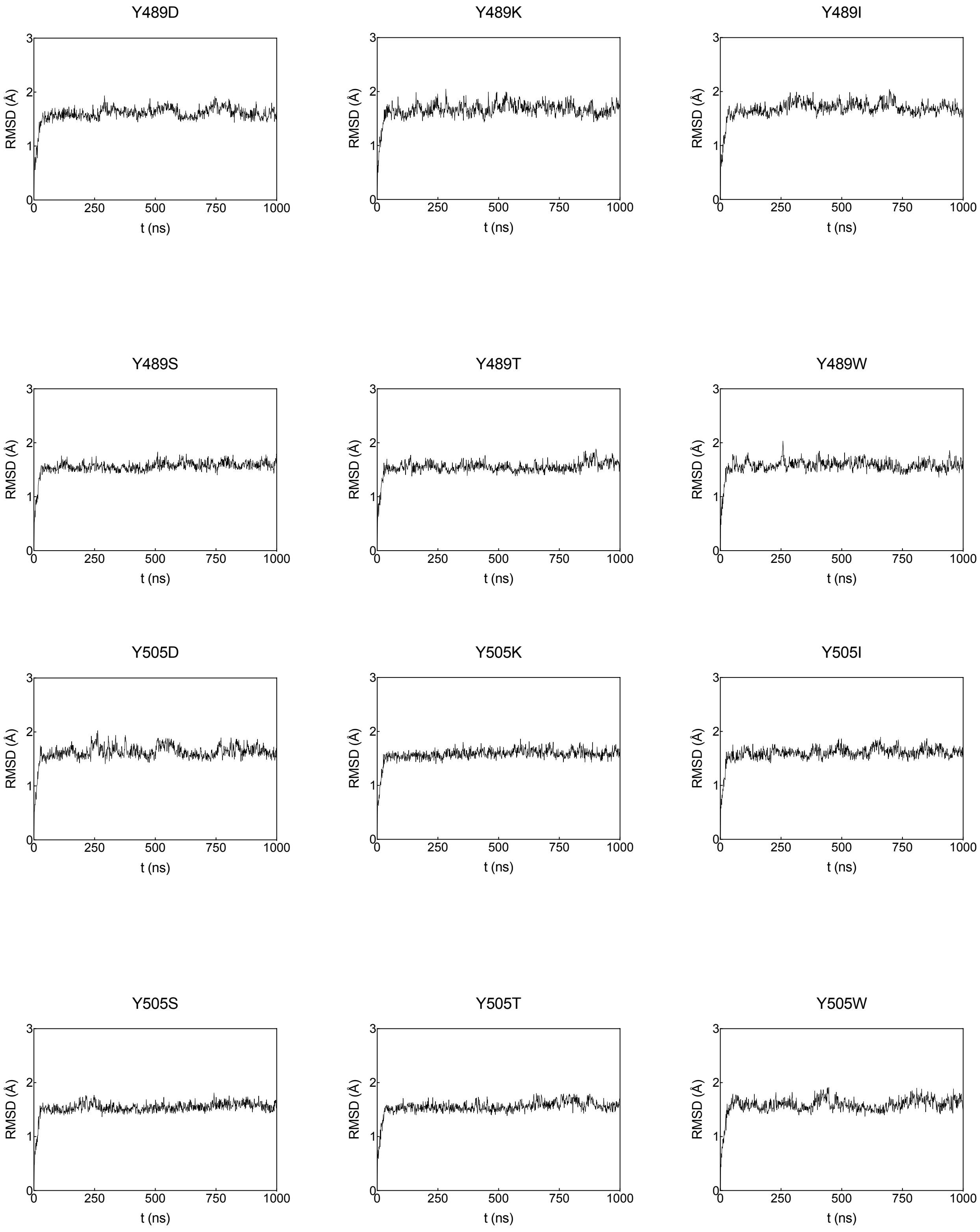
Y489D
Y489K
Y489I
Y489S
Y489T
Y489W
Y505D
Y505K
Y505I
Y505S
Y505T
Y505W
RMSD (Å)
t (ns)
0
1
2
3
250
500
750
1000

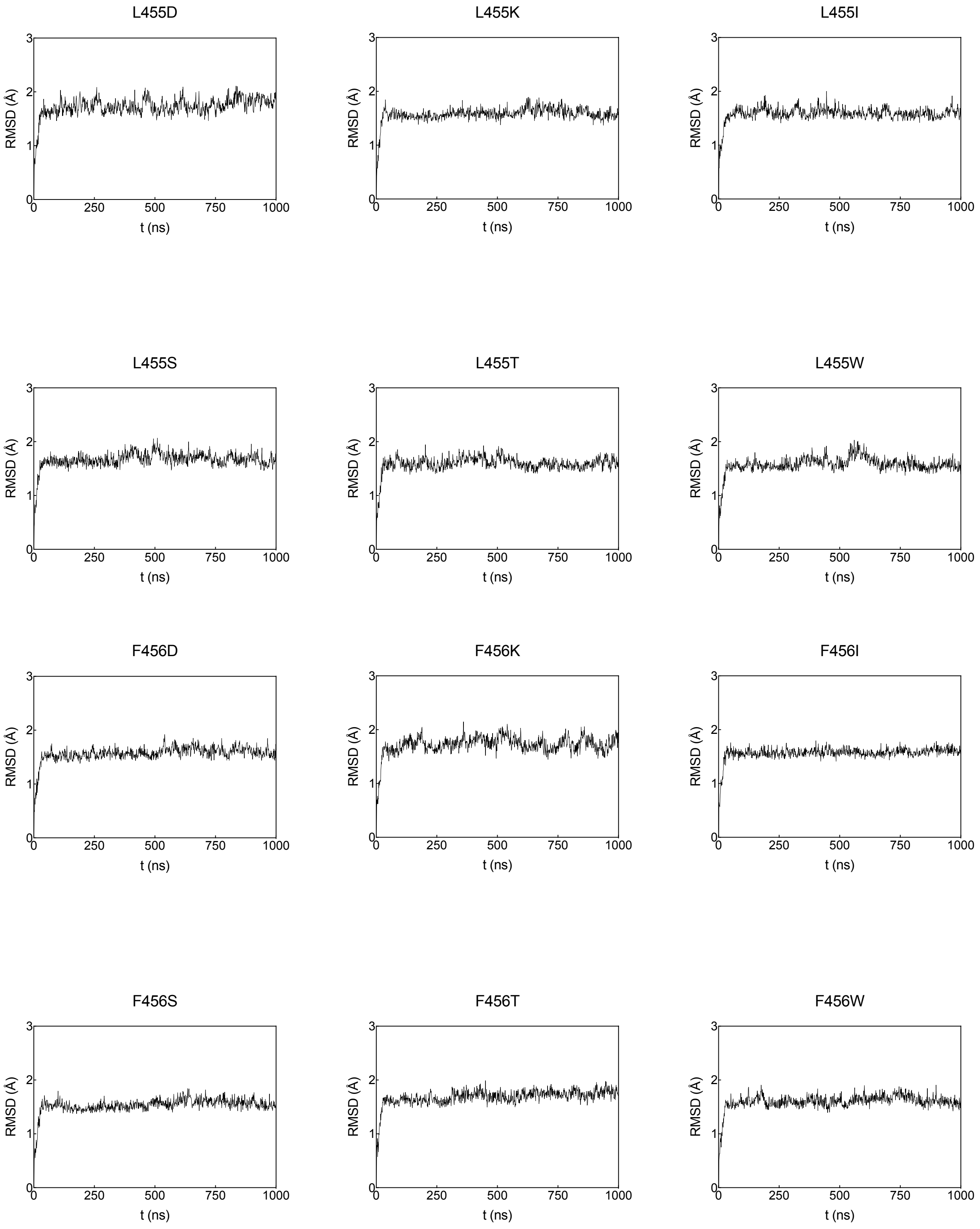
L455D
L455K
L455I
L455S
L455T
L455W
F456D
F456K
F456I
F456S
F456T
F456W
RMSD (Å)
t (ns)
0
250
500
750
1000
0
1
2
3

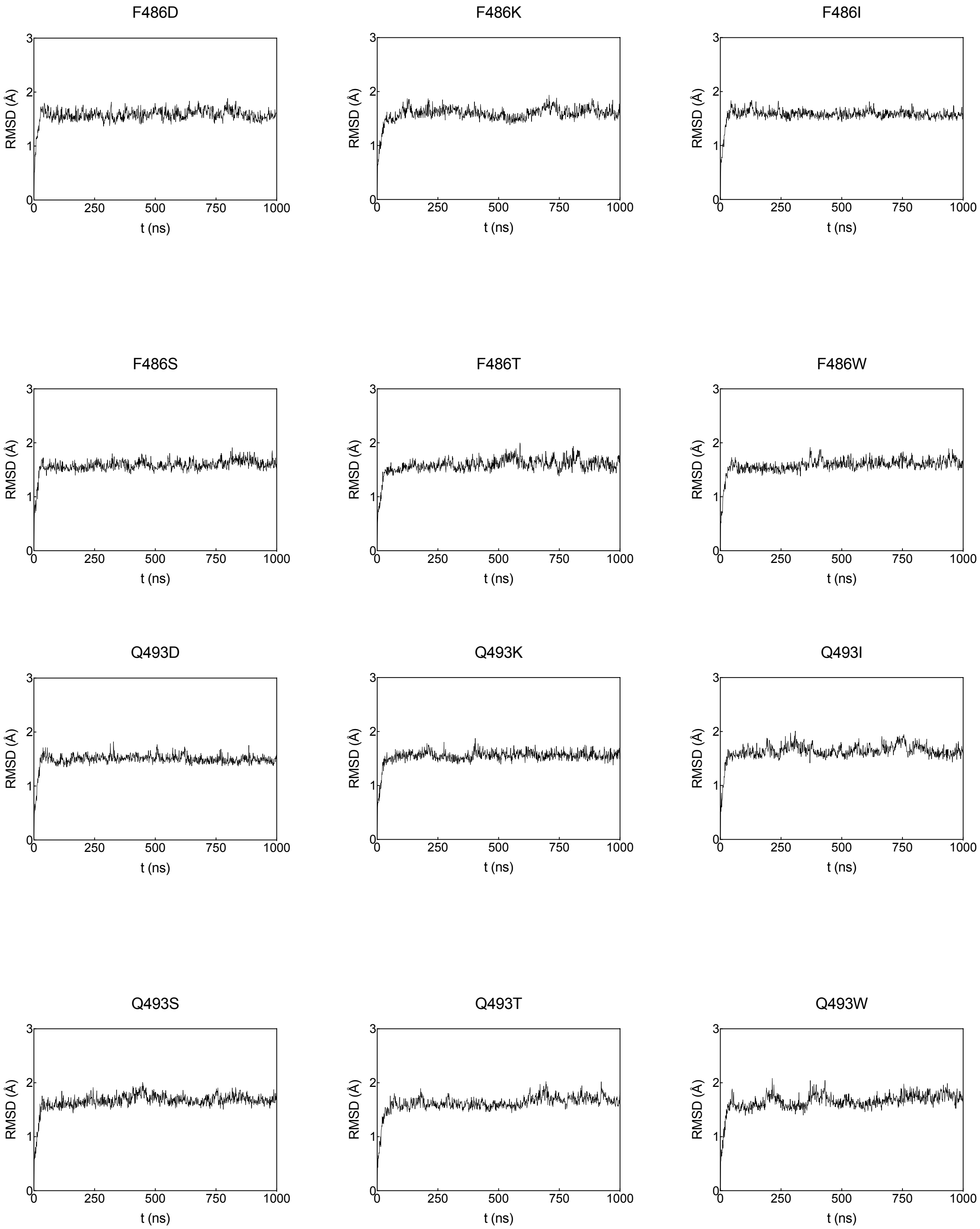
F486D
F486K
F486I
F486S
F486T
F486W
Q493D
Q493K
Q493I
Q493S
Q493T
Q493W
RMSD (Å)
t (ns)
0
1
2
3
250
500
750
1000

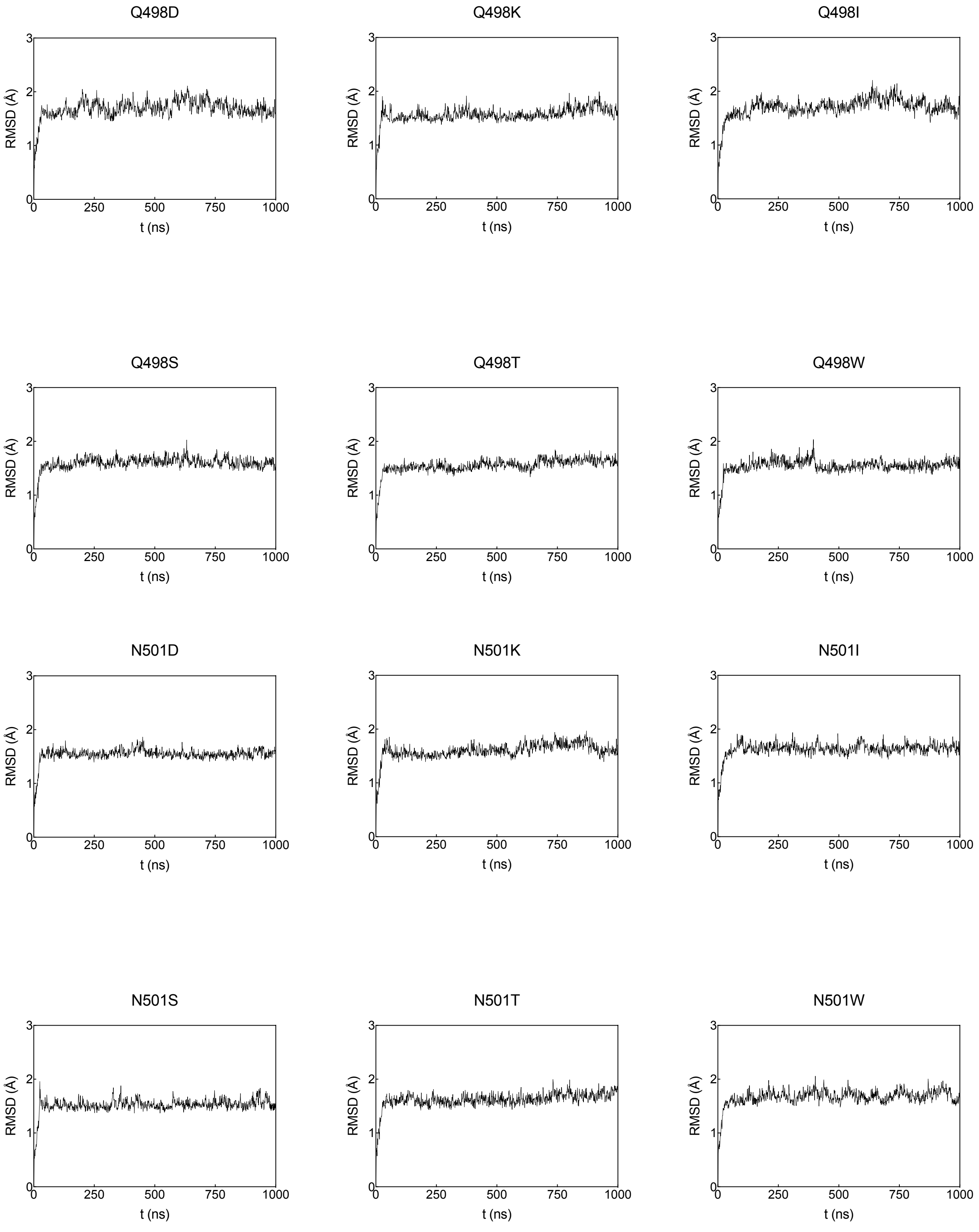
Q498D
Q498K
Q498I
Q498S
Q498T
Q498W
N501D
N501K
N501I
N501S
N501T
N501W
RMSD (Å)
t (ns)
0
1
2
3
0
250
500
750
1000

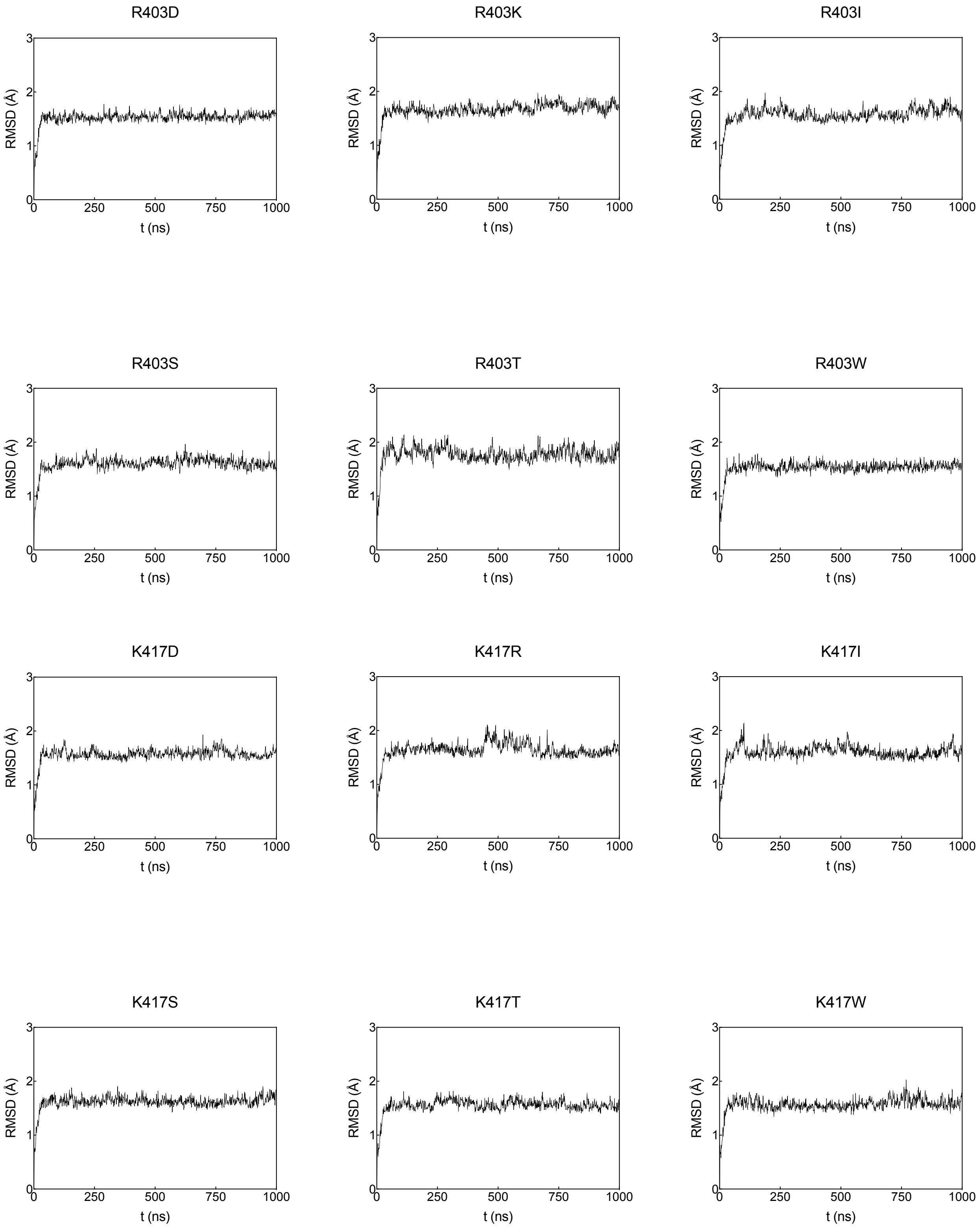


**Figure S34.** Root-mean-square deviation (RMSD) of ACE2/S-$RBD_{CoV-2}$ protein complex backbone atoms as a function of MD simulation time for all S-$RBD_{CoV-2}$ mutants.

**Extended Methods Section**

The optimized structure of the ACE2/S-RBD$_{CoV-2}$ complex (PDB ID 6M0J)[1] was taken from our previous work. The coordinates files and force field parameters for the ACE2 $Zn^{2+}$ binding site, and the input coordinate file (PDB) were already released as Supporting Information of our previous work[2] available at https://pubs.acs.org/doi/10.1021/acsnano.0c04674.

All calculations reported in this work were performed in AMBER19.[3] The *tleap* software provided within AMBER19 was used to assign the ff14SB[4] and GLYCAM06j-1[5] forcefields to the starting protein/protein structure.

All ACE2 and S-RBD$_{CoV-2}$ mutations were introduced into the wild-type structure of the protein-protein complex by swapping the mutant residue into the specific site, according to a procedure we extensively validated through the years for different protein/protein and protein/ligand complexes.[6-25]

Each resultant protein/protein assembly was next subjected to solvation and equilibration protocols, as described in details in our previous work. Briefly, each complex was immersed in a box filled with TIP3PB[26] water molecules and naturalized with the addition of a suitable number of $Na^+$ and $Cl^-$ counterions; next the ionic strength of the system was in order to mimic a physiological salt concentration (0.15 M). Each supermolecular assembly was further optimized progressively removing initial restraints *via* a sequence of: 1) energy minimization (3000 steps of steepest descent followed by 3000 steps of conjugated gradient algorithms), 2) heating to 150 K (in 10 ps of canonical ensemble (NVT) molecular dynamics (MD) followed by 50 ps MD simulation in the isothermal/isobaric ensemble (NPT, P = 1 atm, maintained by the Berendsen barostat[27]) to reach the target temperature of 300 K; 3) further 10 ns in NPT conditions (*phase 1*) and 4) MD data production runs continued up to 1 μs, during which pressure was maintained using the Monte Carlo barostat implemented in AMBER (*phase 2*).

Electrostatic interactions were computed by means of the particle mesh Ewald (PME)[28] algorithm temperature was regulated by the Langevin method[29] (collision frequency of 3 $ps^{-1}$). The SHAKE algorithm[30] was applied to allow a 2 fs integration time step. All calculations were run with the *pmemd* module of AMBER19 running on the supercomputer Marconi100 (CINECA, Bologna, Italy) and on our CPU/GPU hybrid cluster. All images were produced by the UCSF Chimera software[31] and on Prism 8 GraphPad Prism version 8.0.0 for Mac (GraphPad Software, San Diego, California USA, www.graphpad.com).

After the first 5 ns of the *phase 2* MD trajectory, 5 ns MD data were selected to calculate enthalpy and entropy contributions. Configurational sampling was preformed accordingly, with a time step of 10 fs; thus, a total of 500 000 snapshots, sufficient for the interaction entropy (IE) calculations,[32-34] were extracted from the relevant MD trajectory for the calculation of the protein/protein residue-specific interactions.

The free energy was calculated for each molecular species (protein/protein complex, ACE2, and S-RBD$_{COV-2}$) in the framework of the MM/PBSA ansatz,[35] and the protein/protein binding free energy was computed as the difference:

$$\Delta G = G_{ACE2/S\text{-}RBDCOV\text{-}2} - (G_{ACE2} + G_{S\text{-}RBDCOV\text{-}2}) = \Delta E_{vdW} + \Delta E_{ELE} + \Delta G_{SOL} - T\Delta S = \Delta H - T\Delta S \quad (1)$$

in which $\Delta E_{vdW}$ and $\Delta E_{ELE}$ represent van der Waals and electrostatic molecular mechanics energies, and $\Delta G_{sol}$ includes the solvation free energy. The internal dielectric constant was set to the values of 2, 3 and 9 for nonpolar, polar, and charged residues,[15,36] respectively. Lastly, the entropic contribution ($T\Delta S$) was explicitly computed from the MD simulation by using the Interaction Entropy (IE) method.[32-34]

The role of the protein/protein interface key residues was calculated with the MM/PBSA method. Accordingly, the difference in the binding free energy between the wild-type (WT) protein and mutant counterpart, ΔΔG, is given by:

$$\Delta\Delta G = \Delta G_{WILD\text{-}TYPE} - \Delta G_{MUTANT} \quad (2)$$

Thus, the adopted methodology allows for the estimation of the contribution of a given residue with respect to the overall protein−protein binding free energy; indeed, according to equation (5), a negative value of ΔΔG indicated a favorable contribution for the wild type residue in that position and vice versa.
At the structural level, the stability of the main protein/protein interface intermolecular and intramolecular interactions detected during the MD simulation time interval adopted for the energetic analysis was assessed along the entire duration of the MD run.

**References**


1. Berman, H. M.; Westbrook, J.; Feng, Z.; Gilliland, G.; Bhat, T. N.; Weissig, H.; Shindyalov, I. N.; Bourne, P. E., The Protein Data Bank. *Nucleic Acids Res* **2000,** *28*, 235-242.
2. Laurini, E.; Marson, D.; Aulic, S.; Fermeglia, M.; Pricl, S., Computational Alanine Scanning and Structural Analysis of the SARS-CoV-2 Spike Protein/Angiotensin-Converting Enzyme 2 Complex. *ACS Nano* **2020,** *14*, 11821-11830.
3. Case, D. A.; Ben-Shalom, I. Y.; Brozell, S. R.; Cerutti, D. S.; Cheatham, I., T.E. ; Cruzeiro, V. W. D.; Darden, T. A.; Duke, R. E.; Ghoreishi, D.; Giambasu, G.; Giese, T.; Gilson, M. K.; Gohlke, H.; Goetz, A. W.; Greene, D.; Harris, R.; Homeyer, N.; Huang, Y.; Izadi, S.; Kovalenko, A., *et al.* AMBER 2019, University of California, San Francisco, CA, USA. **2019**.
4. Maier, J. A.; Martinez, C.; Kasavajhala, K.; Wickstrom, L.; Hauser, K. E.; Simmerling, C., ff14SB: Improving the Accuracy of Protein Side Chain and Backbone Parameters from ff99SB. *J Chem Theory Comput* **2015,** *11*, 3696-3713.
5. Kirschner, K. N.; Yongye, A. B.; Tschampel, S. M.; Gonzalez-Outeirino, J.; Daniels, C. R.; Foley, B. L.; Woods, R. J., GLYCAM06: A Generalizable Biomolecular Force Field. Carbohydrates. *J Comput Chem* **2008,** *29*, 622-655.
6. Pricl, S.; Fermeglia, M.; Ferrone, M.; Tamborini, E., T315I-Mutated Bcr-Abl in Chronic Myeloid Leukemia and Imatinib: Insights from a Computational Study. *Mol Cancer Ther* **2005,** *4*, 1167-1174.
7. Ferrone, M.; Perrone, F.; Tamborini, E.; Paneni, M. S.; Fermeglia, M.; Suardi, S.; Pastore, E.; Delia, D.; Pierotti, M. A.; Pricl, S.; Pilotti, S., Functional Analysis and Molecular Modeling Show a Preserved Wild-Type Activity of p53(C238Y). *Mol Cancer Ther* **2006,** *5*, 1467-1473.
8. Tamborini, E.; Pricl, S.; Negri, T.; Lagonigro, M. S.; Miselli, F.; Greco, A.; Gronchi, A.; Casali, P. G.; Ferrone, M.; Fermeglia, M.; Carbone, A.; Pierotti, M. A.; Pilotti, S., Functional Analyses and Molecular Modeling of Two C-Kit Mutations Responsible for Imatinib Secondary Resistance in GIST Patients. *Oncogene* **2006,** *25*, 6140-6146.
9. McAuliffe, J. C.; Wang, W. L.; Pavan, G. M.; Pricl, S.; Yang, D.; Chen, S. S.; Lazar, A. J.; Pollock, R. E.; Trent, J. C., Unlucky Number 13? Differential Effects of KIT Exon 13 Mutation in Gastrointestinal Stromal Tumors. *Mol Oncol* **2008,** *2*, 161-163.
10. Conca, E.; Negri, T.; Gronchi, A.; Fumagalli, E.; Tamborini, E.; Pavan, G. M.; Fermeglia, M.; Pierotti, M. A.; Pricl, S.; Pilotti, S., Activate and Resist: L576P-KIT in GIST. *Mol Cancer Ther* **2009,** *8*, 2491-2495.

11. Negri, T.; Pavan, G. M.; Virdis, E.; Greco, A.; Fermeglia, M.; Sandri, M.; Pricl, S.; Pierotti, M. A.; Pilotti, S.; Tamborini, E., T670X KIT Mutations in Gastrointestinal Stromal Tumors: Making Sense of Missense. *J Natl Cancer Inst* **2009,** *101*, 194-204.
12. Woodman, S. E.; Trent, J. C.; Stemke-Hale, K.; Lazar, A. J.; Pricl, S.; Pavan, G. M.; Fermeglia, M.; Gopal, Y. N.; Yang, D.; Podoloff, D. A.; Ivan, D.; Kim, K. B.; Papadopoulos, N.; Hwu, P.; Mills, G. B.; Davies, M. A., Activity of Dasatinib against L576P KIT Mutant Melanoma: Molecular, Cellular, and Clinical Correlates. *Mol Cancer Ther* **2009,** *8*, 2079-2085.
13. Dileo, P.; Pricl, S.; Tamborini, E.; Negri, T.; Stacchiotti, S.; Gronchi, A.; Posocco, P.; Laurini, E.; Coco, P.; Fumagalli, E.; Casali, P. G.; Pilotti, S., Imatinib Response in Two GIST Patients Carrying Two Hitherto Functionally Uncharacterized PDGFRA Mutations: An Imaging, Biochemical and Molecular Modeling Study. *Int J Cancer* **2011,** *128*, 983-990.
14. Bozzi, F.; Conca, E.; Laurini, E.; Posocco, P.; Lo Sardo, A.; Jocollè, G.; Sanfilippo, R.; Gronchi, A.; Perrone, F.; Tamborini, E.; Pelosi, G.; Pierotti, M. A.; Maestro, R.; Pricl, S.; Pilotti, S., *In Vitro* and *in Silico* Studies of MDM2/MDMX Isoforms Predict Nutlin-3A Sensitivity in Well/De-Differentiated Liposarcomas. *Lab Invest* **2013,** *93*, 1232-1240.
15. Conca, E.; Miranda, C.; Dal Col, V.; Fumagalli, E.; Pelosi, G.; Mazzoni, M.; Fermeglia, M.; Laurini, E.; Pierotti, M. A.; Pilotti, S.; Greco, A.; Pricl, S.; Tamborini, E., Are Two Better than One? A Novel Double-Mutant KIT in GIST that Responds to Imatinib. *Mol Oncol* **2013,** *7*, 756-762.
16. Laurini, E.; Posocco, P.; Fermeglia, M.; Gibbons, D. L.; Quintás-Cardama, A.; Pricl, S., Through the Open Door: Preferential Binding of Dasatinib to the Active Form of BCR-ABL Unveiled by *in Silico* Experiments. *Mol Oncol* **2013,** *7*, 968-975.
17. Brune, S.; Schepmann, D.; Klempnauer, K. H.; Marson, D.; Dal Col, V.; Laurini, E.; Fermeglia, M.; Wünsch, B.; Pricl, S., The Sigma Enigma: *In Vitro/in Silico* Site-Directed Mutagenesis Studies Unveil σ1 Rceptor Lgand Binding. *Biochemistry* **2014,** *53*, 2993-3003.
18. Gibbons, D. L.; Pricl, S.; Posocco, P.; Laurini, E.; Fermeglia, M.; Sun, H.; Talpaz, M.; Donato, N.; Quintás-Cardama, A., Molecular Dynamics Reveal BCR-ABL1 Polymutants as a Unique Mechanism of Resistance to PAN-BCR-ABL1 Kinase Inhibitor Therapy. *Proc Natl Acad Sci U S A* **2014,** *111*, 3550-3555.
19. Brambilla, L.; Genini, D.; Laurini, E.; Merulla, J.; Perez, L.; Fermeglia, M.; Carbone, G. M.; Pricl, S.; Catapano, C. V., Hitting the Rght Spot: Mechanism of Action of OPB-31121, a Novel and Potent Inhibitor of the Signal Transducer and Activator of Transcription 3 (STAT3). *Mol Oncol* **2015,** *9*, 1194-1206.
20. Morgan, A.; Gandin, I.; Belcaro, C.; Palumbo, P.; Palumbo, O.; Biamino, E.; Dal Col, V.; Laurini, E.; Pricl, S.; Bosco, P.; Carella, M.; Ferrero, G. B.; Romano, C.; d'Adamo, A. P.; Faletra, F.; Vozzi, D., Target Sequencing Approach Intended to Discover New Mutations in Non-Syndromic Intellectual Disability. *Mutat Res* **2015,** *781*, 32-36.
21. Pricl, S.; Cortelazzi, B.; Dal Col, V.; Marson, D.; Laurini, E.; Fermeglia, M.; Licitra, L.; Pilotti, S.; Bossi, P.; Perrone, F., Smoothened (SMO) Receptor Mutations Dictate Resistance to Vismodegib in Basal Cell Carcinoma. *Mol Oncol* **2015,** *9* (2), 389-397.
22. Colombo, C.; Belfiore, A.; Paielli, N.; De Cecco, L.; Canevari, S.; Laurini, E.; Fermeglia, M.; Pricl, S.; Verderio, P.; Bottelli, S.; Fiore, M.; Stacchiotti, S.; Palassini, E.; Gronchi, A.; Pilotti, S.; Perrone, F., β-Catenin in Desmoid-Type Fibromatosis: Deep Insights into the Role of T41A and S45F Mutations on Protein Structure and Gene Expression. *Mol Oncol* **2017,** *11*, 1495-1507.
23. Genini, D.; Brambilla, L.; Laurini, E.; Merulla, J.; Civenni, G.; Pandit, S.; D'Antuono, R.; Perez, L.; Levy, D. E.; Pricl, S.; Carbone, G. M.; Catapano, C. V., Mitochondrial Dysfunction

Induced by a SH2 Domain-Targeting STAT3 Inhibitor Leads to Metabolic Synthetic Lethality in Cancer Cells. *Proc Natl Acad Sci U S A* **2017,** *114*, e4924-e4933.
24. Perfetti, V.; Laurini, E.; Aulić, S.; Fermeglia, M.; Riboni, R.; Lucioni, M.; Dallera, E.; Delfanti, S.; Pugliese, L.; Latteri, F. S.; Pietrabissa, A.; Pricl, S., Molecular and Functional Characterization of a New 3' End KIT Juxtamembrane Deletion in a Duodenal GIST Treated with Neoadjuvant Imatinib. *Oncotarget* **2017,** *8*, 56158-56167.
25. Ziouziou, H.; Andrieu, C.; Laurini, E.; Karaki, S.; Fermeglia, M.; Oueslati, R.; Taieb, D.; Camplo, M.; Siri, O.; Pricl, S.; Katsogiannou, M.; Rocchi, P., Targeting Hsp27/eIF4E Interaction with Phenazine Compound: A Promising Alternative for Castration-Resistant Prostate Cancer Treatment. *Oncotarget* **2017,** *8*, 77317-77329.
26. Jorgensen, W. L.; Chandrasekhar, J.; Madura, J. D.; Impey, R. W.; Klein, M. L., Comparison of Simple Potential Functions for Simulating Liquid Water. *J Chem Phys* **1983,** *79* (2), 926-935.
27. Berendsen, H. J. C.; Postma, J. P. M.; van Gunsteren, W. F.; DiNola, A.; Haak, J. R., Molecular Dynamics with Coupling to an External Bath. *J Chem Phys* **1984,** *81*, 3684-3690.
28. Toukmaji, A.; Sagui, C.; Board, J.; Darden, T., Efficient Particle-Mesh Ewald Based Approach to Fixed and Induced Dipolar Interactions. *J Chem Phys* **2000,** *113*, 10913–10927.
29. Loncharich, R. J.; Brooks, B. R.; Pastor, R. W., Langevin Dynamics of Peptides: The Frictional Dependence of Isomerization Rates of N-Acetylalanyl-N'-Methylamide. *Biopolymers* **1992,** *32*, 523–535.
30. Ryckaert, J.-P.; Ciccotti, G.; Berendsen, H. J. C., Numerical Integration of the Cartesian Equations of Motion of a System with Constraints: Molecular Dynamics of N-Alkanes. *J Comput Phys* **1977,** *23*, 327-341.
31. Pettersen, E. F.; Goddard, T. D.; Huang, C. C.; Couch, G. S.; Greenblatt, D. M.; Meng, E. C.; Ferrin, T. E., UCSF Chimera--A Visualization System for Exploratory Research and Analysis. *J Comput Chem* **2004,** *25*, 1605–1612.
32. Yan, Y.; Yang, M.; Ji, C. G.; Zhang, J. Z. H., Interaction Entropy for Computational Alanine Scanning. *J Chem Inf Model* **2017,** *57*, 1112-1122.
33. Sun, Z.; Yan, Y. N.; Yang, M.; Zhang, J. Z., Interaction Entropy for Protein-Protein Binding. *J Chem Phys* **2017,** *146*, 124124.
34. Liu, X.; Peng, L.; Zhou, Y.; Zhang, Y.; Zhang, J. Z. H., Computational Alanine Scanning with Interaction Entropy for Protein–Ligand Binding Free Energies. *J Chem Theory Comput* **2018,** *14*, 1772-1780.
35. Wang, E.; Sun, H.; Wang, J.; Wang, Z.; Liu, H.; Zhang, J. Z. H.; Hou, T., End-Point Binding Free Energy Calculation with MM/PBSA and MM/GBSA: Strategies and Applications in Drug Design. *Chem Rev* **2019,** *119*, 9478-9508.
36. Yan, Y.; Yang, M.; Ji, C. G.; Zhang, J. Z. H., Interaction Entropy for Computational Alanine Scanning. *J Chem Inf Model* **2017,** *57*, 1112-1122.
